# Supplementary material for: Smad4 controls signaling robustness and morphogenesis by differentially contributing to the Nodal and BMP pathways
Source: Nat Commun. 2021 Nov 4;12:6374. doi: 10.1038/s41467-021-26486-3 (PMC8569018; doi:10.1038/s41467-021-26486-3)
Supplement: Supplementary file 1 — Supplementary Information [file 41467_2021_26486_MOESM1_ESM.pdf]

Smad4 controls signaling robustness and morphogenesis by differentially contributing to the Nodal and BMP pathways

Guglielmi et al

Supplementary Tables and Figures

Supplementary Fig. 1. The mutant phenotype is specific to the loss of *smad4a.*

Supplementary Fig. 2. scRNA-seq reveals the pseudo-spatial expression pattern of BMP and Nodal components at the onset of gastrulation.

Supplementary Fig. 3. BMP target gene expression is dependent on Smad4a, but Nodal target gene expression is mostly independent of Smad4a and Smad4b.

Supplementary Fig. 4. SMAD4 is differentially required for BMP and Nodal/Activin signaling in mESCs.

Supplementary Fig. 5. Loss of BMP signaling does not affect Nodal activity in the embryo.

Supplementary Fig. 6. Loss of BMP signaling dominates effects on germ layer formation and morphogenesis in MZ*smad4a* embryos*.*

Supplementary Fig. 7. OPT images for embryos treated with different doses of DMH1.

Supplementary Fig. 8. Defining BMP morphospace.

Supplementary Table 1. List of oligonucleotides.

Supplementary Table 2. Key resources

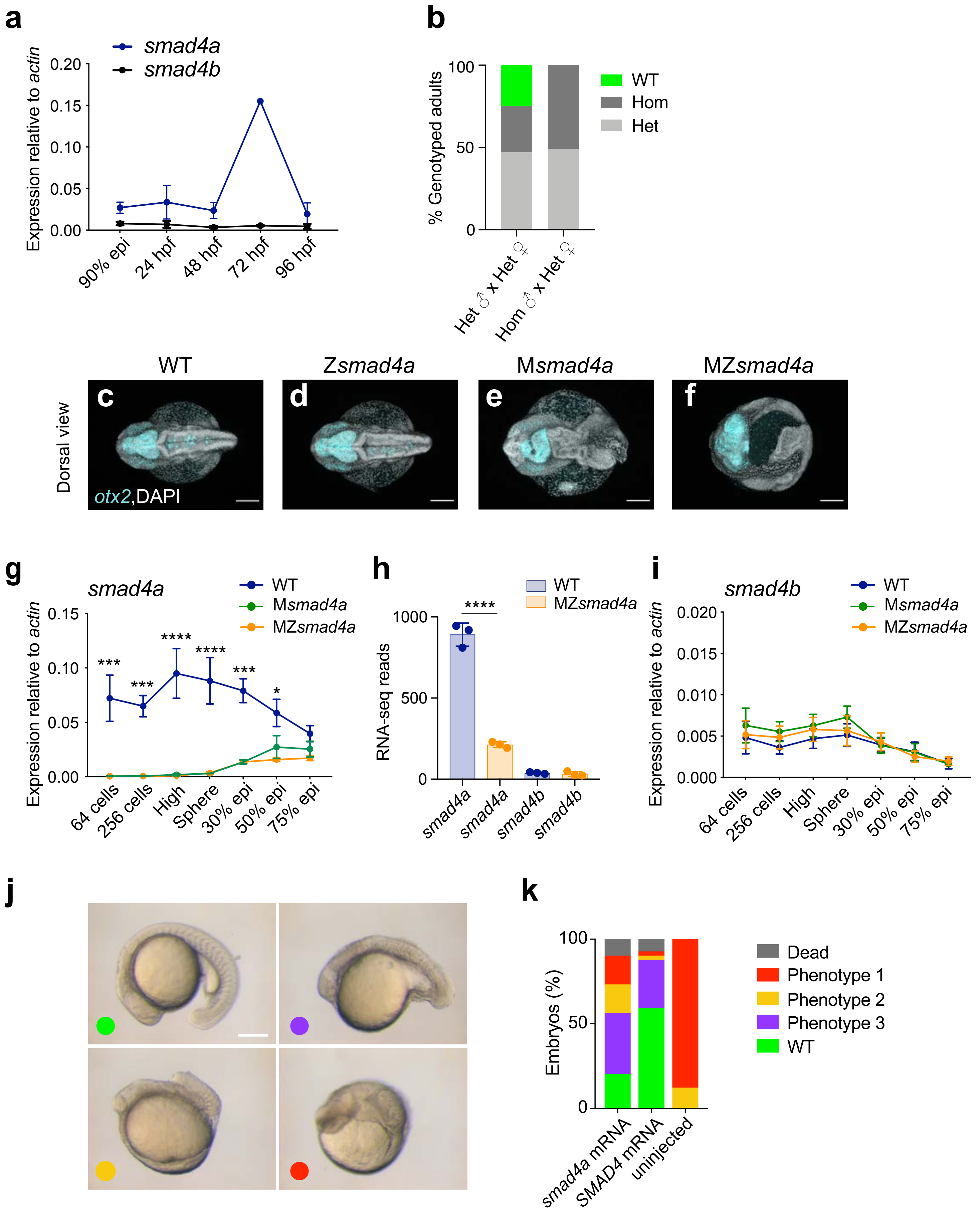

a
smad4a
smad4b
Expression relative to actin
0.20
0.15
0.10
0.05
0.00
90% epi
24 hpf
48 hpf
72 hpf
96 hpf
b
% Genotyped adults
100
50
0
WT
Hom
Het
Het ♂ x Het ♀
Hom ♂ x Het ♀
WT
Zsmad4a
Msmad4a
MZsmad4a
Dorsal view
c
d
e
f
otx2,DAPI
g
smad4a
WT
Msmad4a
MZsmad4a
Expression relative to actin
0.15
0.10
0.05
0.00
***
***
****
****
***
*
64 cells
256 cells
High
Sphere
30% epi
50% epi
75% epi
h
WT
MZsmad4a
****
RNA-seq reads
1000
500
0
smad4a
smad4a
smad4b
smad4b
i
smad4b
WT
Msmad4a
MZsmad4a
Expression relative to actin
0.020
0.015
0.010
0.005
0.000
64 cells
256 cells
High
Sphere
30% epi
50% epi
75% epi
j
k
Embryos (%)
100
50
0
Dead
Phenotype 1
Phenotype 2
Phenotype 3
WT
smad4a mRNA
SMAD4 mRNA
uninjected

Supplementary Fig. 1. The mutant phenotype is specific to the loss of *smad4a.* **a** qPCR for *smad4a* and *smad4b* mRNA in WT embryos at the stages indicated. Means ± SEM are shown for 2 biological replicates for each timepoint. **b** Quantitation of zygotic *smad4a* mutants reaching adulthood. Parental crosses and genotypes are indicated. **c–f** Anterior views of double-FISH-stained 24-hpf WT (**c**), Z*smad4a* (**d**), M*smad4a* (**e**) and MZ*smad4a* (**f**) embryos shown in Fig. 1d–g where only the *otx2* staining is shown (cyan). Nuclei are stained with DAPI (white). Scale bar corresponds to 150 µm. Images in **c–f** are representative of 25 embryos each and 4 independent experiments. **g** qPCR for *smad4a* mRNA in WT, M*smad4a* and MZ*smad4a* embryos at the stages indicated. Means ± SEM are shown for four biological replicates for each timepoint and each genotype. The WT data shown are the same as Fig. 1a for comparison. The statistics are only shown for the WT and MZ*smad4a* comparison. 64 cells: p(adj) = 3.837×10^{-4}, 256 cells: p(adj) = 9.951×10^{-4}, High: p(adj) = $6{,}377\times10^{-6}$, Sphere: p(adj) = 3.532×10^{-5}, 30% epiboly: p(adj) = $9{,}951\times10^{-4}$, 50% epiboly: p(adj) = 0.023. Unpaired multiple comparison t-test with Holm-Sidack correction. *, $p<0.05$; ***, $p<0.001$; ****, $p<0.0001$. **h** RNA-seq reads for *smad4a* and *smad4b* mRNA in WT and MZ*smad4a* embryos. Note that values for *smad4a* are the same as those shown in Fig. 1h to allow comparison. Means ± SD are shown from three biological replicates for each genotype. p(adj) = 2.132×10^{-35}. Wald test. ****, p(adj)<0.0001. **i** qPCR for *smad4b* mRNA in WT, M*smad4a* and MZ*smad4a* embryos at the stages indicated. Means ± SEM are shown for four biological replicates for each timepoint and each genotype. The WT data shown are the same as in Fig. 1a for comparison. **j** Representative images of rescued MZ*smad4a* embryos. Colored dots refer to different degrees of rescue as quantified in **k**. Scale bar corresponds to 300 µm. **k** Quantitation of the phenotypes in zebrafish embryos injected with either human *SMAD4* mRNA or zebrafish *smad4a* mRNA. Colors refer to the different phenotypes shown in **j**.

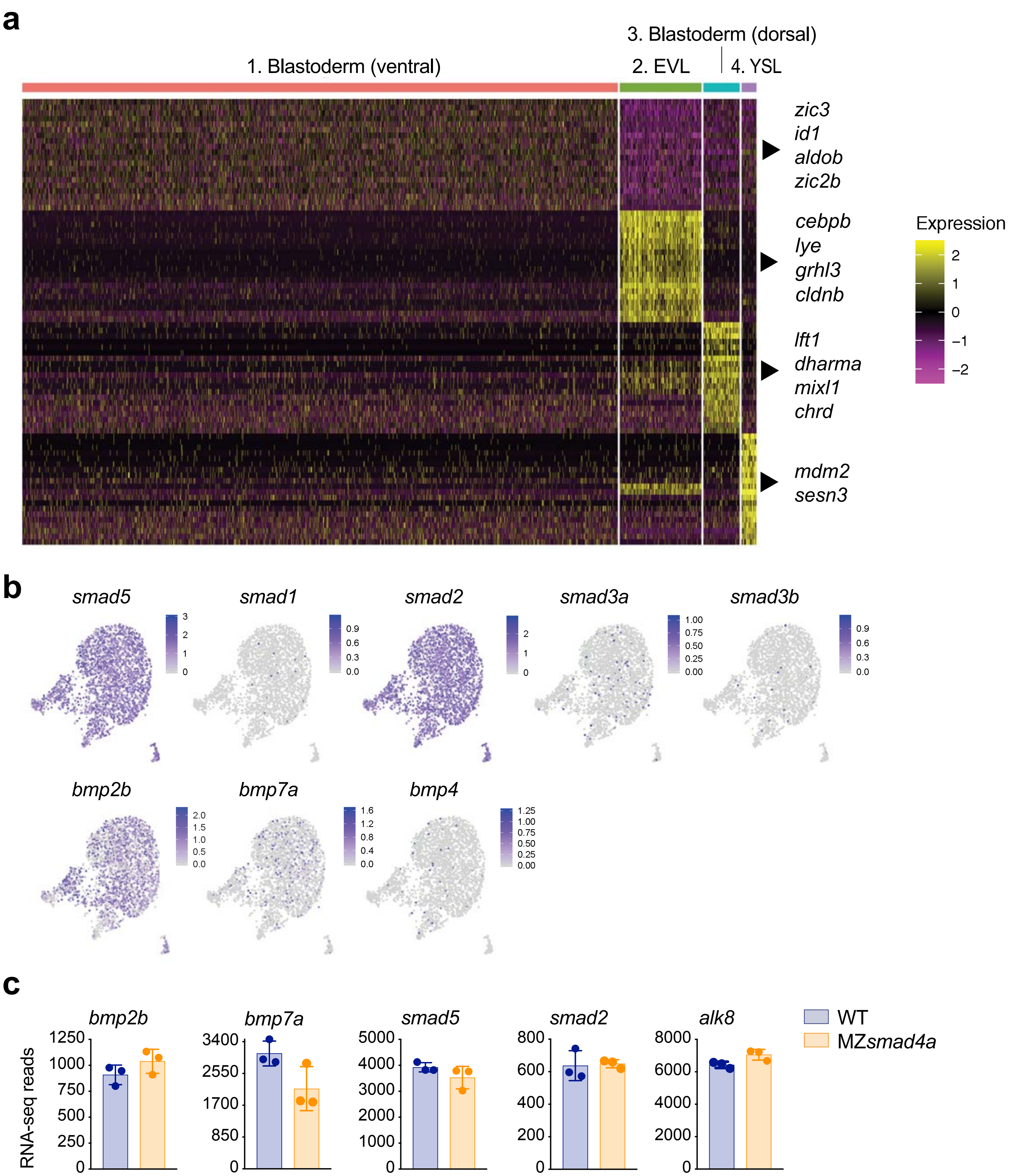

Supplementary Fig. 2

Supplementary Fig. 2. scRNA-seq reveals the pseudo-spatial expression pattern of BMP and Nodal components at the onset of gastrulation. a Heatmap representing the 4 different clusters shown in Fig. 1b. The genes used for cluster definition are indicated. **b** Uniform manifold approximation and projection (UMAP) visualization of single cells derived from sphere stage zebrafish embryos, showing expression of Nodal and BMP pathway components. Normalized counts are shown. **c** RNA-seq reads for Nodal and BMP pathway components at 50% epiboly in WT and MZ*smad4a* mutant embryos. Means ± SD are shown from 3 biological replicates for each genotype. Wald test. p(adj) > 0.01 for all markers.

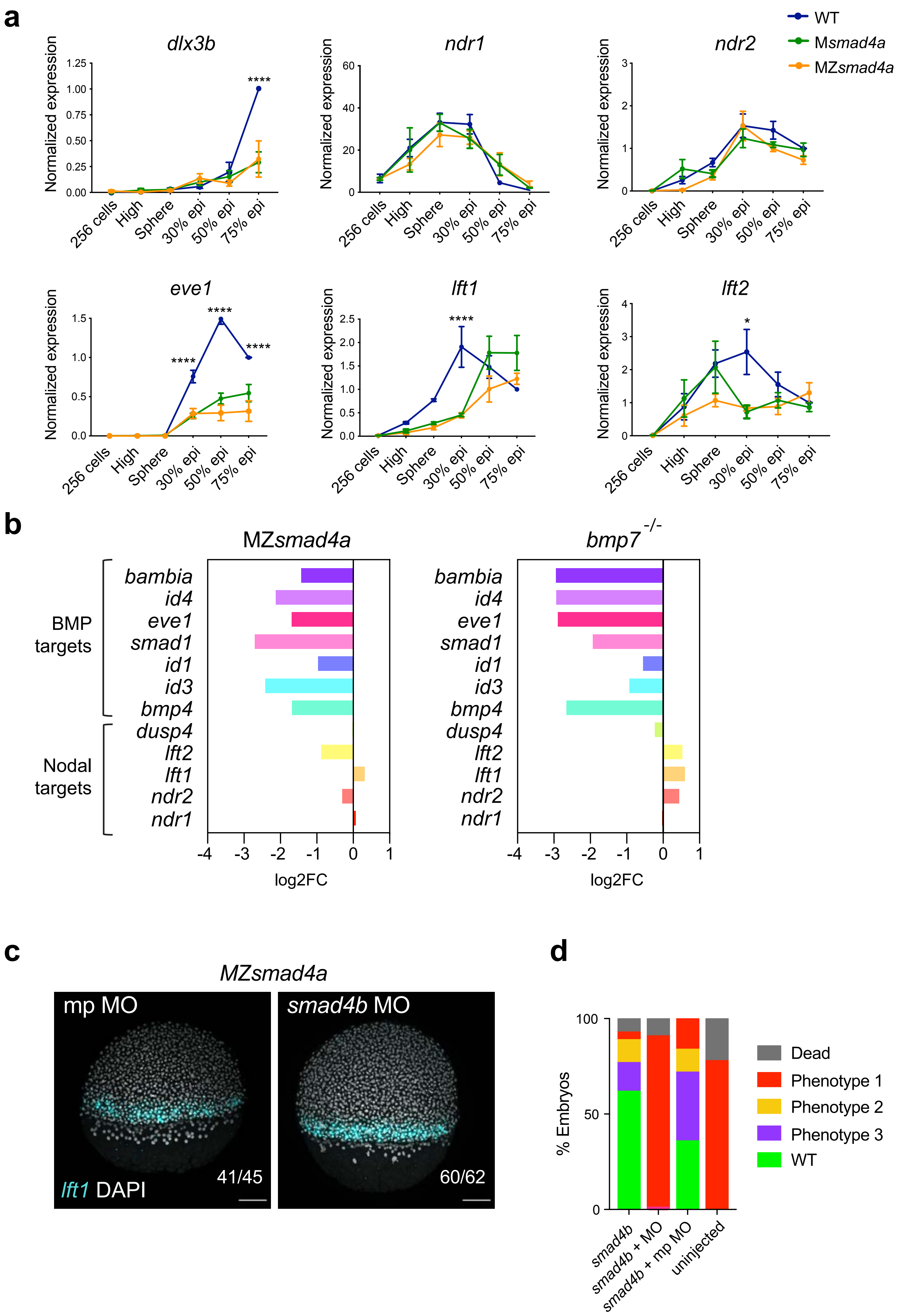

Supplementary Fig. 3

Supplementary Fig. 3. BMP target gene expression is dependent on Smad4a, but Nodal target gene expression is mostly independent of Smad4a and Smad4b. a qPCR for *dlx3b, ndr1, ndr2, eve1, lft1* and *lft2* mRNA in WT, M*smad4a* and MZ*smad4a* embryos at the stages indicated. Means ± SEM are shown. The data for *ndr1/2* are the result of 3 biological replicates while for *lft1/2* they are the result of 4 biological replicates, except for *lft1* in MZ*smad4a* at sphere stage and *lft2* in WT at 30% epiboly, which are the result of 3 biological replicates. The data for *eve1* and *dlx3b* are the result of 4 biological replicates, except for *dlx3b* in WT at 30% epiboly and *eve1* in M*smad4a* at 30% epiboly, which are the result of 3 biological replicates. Statistics are shown for the comparison between WT and M*smad4a*. *dlx3b* 75% epiboly: p(adj) = 4.207×10^{-12}, *eve1* 30% epiboly: p(adj) = 4.811×10^{-7}, *eve1* 50% epiboly: p(adj) = 1.0×10^{-15}, *eve1* 75% epiboly: p(adj) = 6.182×10^{-7}, *lft1* 30% epiboly: p(adj) = 9.746×10^{-5}, *lft2* 30% epiboly: p(adj) = 0.024. Unpaired multiple comparison T-test with Holm-Sidack correction. *, $p<0.05$; ****, $p<0.0001$. Note that for *lft1/2 and ndr1/2* the WT and MZ*smad4a* traces are the same as in Fig. 2c for comparison purposes. See Fig. 2c for the statistics for this comparison. **b** RNA-seq data for BMP and Nodal target genes in MZ*smad4a* (left panel) and *bmp7*$^{-/-}$ (right panel) embryos compared to their respective WT controls. Data are expressed as log2FC, obtained by the comparison of mutant embryos replicates with their respective WT controls. The signaling pathways are indicated, as are the specific markers. **c** ISH for *lft1* in MZ*smad4a* embryos. Embryos were injected either with *smad4b* miss-paired (mp) MO or with the *smad4b* MO as indicated. Scale bar corresponds to 100 µm. Nuclei were stained with DAPI (white). **d** Quantitation of MZ*smad4a* embryos injected with *smad4b* mRNA ± *smad4b* MO or *smad4b* mp MO or uninjected. Colors refer to phenotypes in Supplementary Fig. 1j.

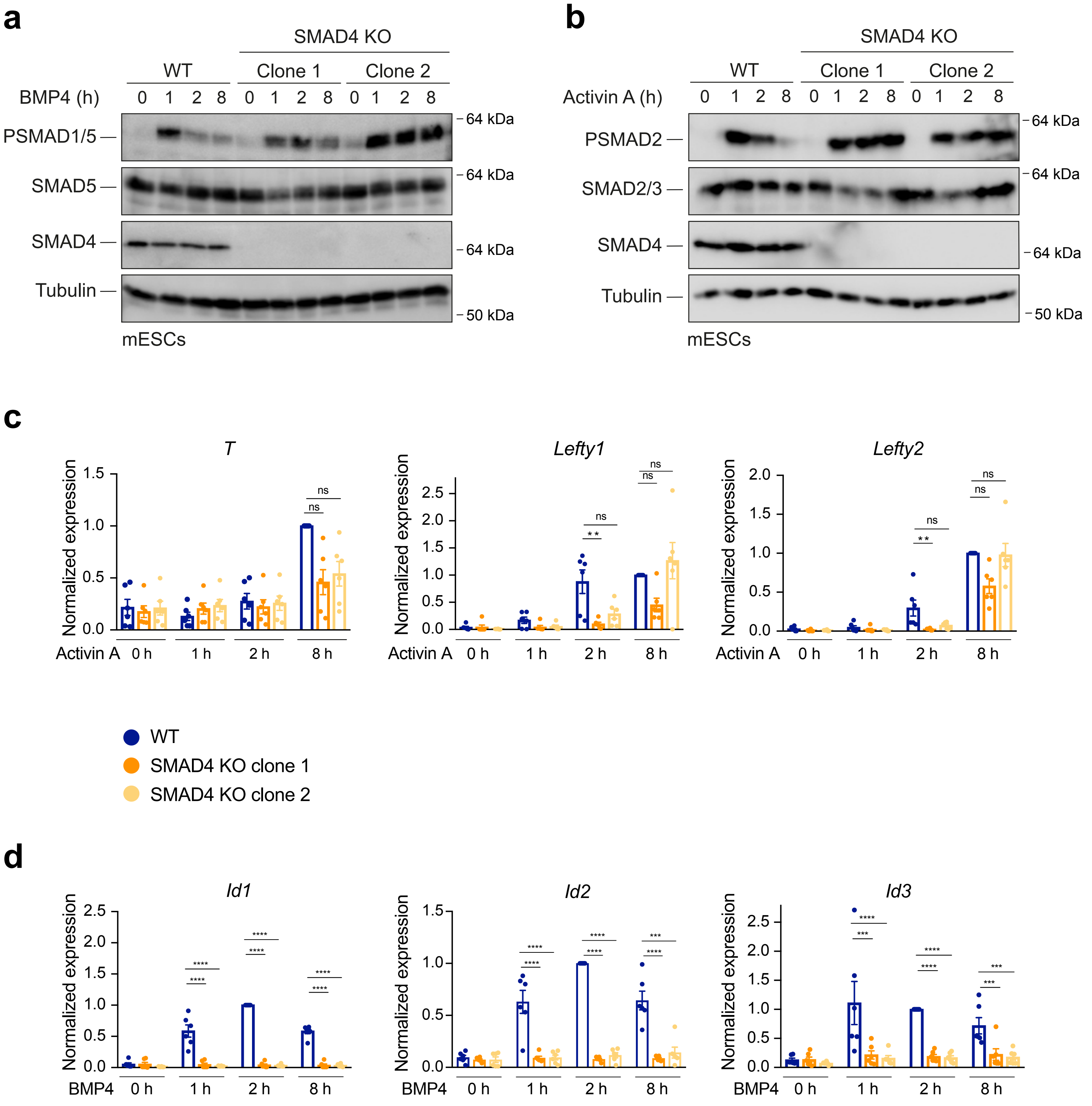

Supplementary Fig. 4

Supplementary Fig. 4. SMAD4 is differentially required for BMP and Nodal/Activin signaling in mESCs. a Immunoblot of whole cell lysates of parental and two SMAD4 KO mESC clones upon different duration of 20 ng/ml BMP4 stimulation. Antibodies used are indicated for each panel. **b** As in **a** but cells were treated for the times indicated with 20 ng/ml Activin A. Immunoblots are representative of 3 independent experiments. Molecular weight markers are given in kDa on the right of the blots. **c** qPCR for *T, Lefty2 and Lefty1* after different durations of Activin A stimulation. Means ± SEM are shown. The data for *Lefty1*, *Lefty2* and *T* are the result of 6 biological replicates. *Lefty1* WT vs. SMAD4 KO clone 1 at 2 h Activin A: p(adj) = 0.004. *Lefty2* WT vs SMAD4 KO clone 1 at 2 h Activin A: p(adj) = 0.001. Two-way Anova and Tukey post hoc test. *p<0.05; **p<0.01; ns, not significant. **d** qPCR for *Id1, Id2 and Id3* after different durations of BMP4 stimulation. Means ± SEM are shown. The data for *Id1/2/3* are the result of 6 biological replicates. p(adj) <0.0001 for all comparisons between WT vs. SMAD4 KO clone1/2 for all genes, except for *Id2* (WT vs. SMAD4 KO clone 2 at 8 h BMP4) and *Id3* (WT vs. SMAD4 KO clone 1 at 1 h BMP4, WT vs. SMAD4 KO clone 1/2 at 8 h BMP4) for which p(adj) <0.001. Two-way Anova and Tukey post hoc test. ***p<0.001; ****p<0.0001.

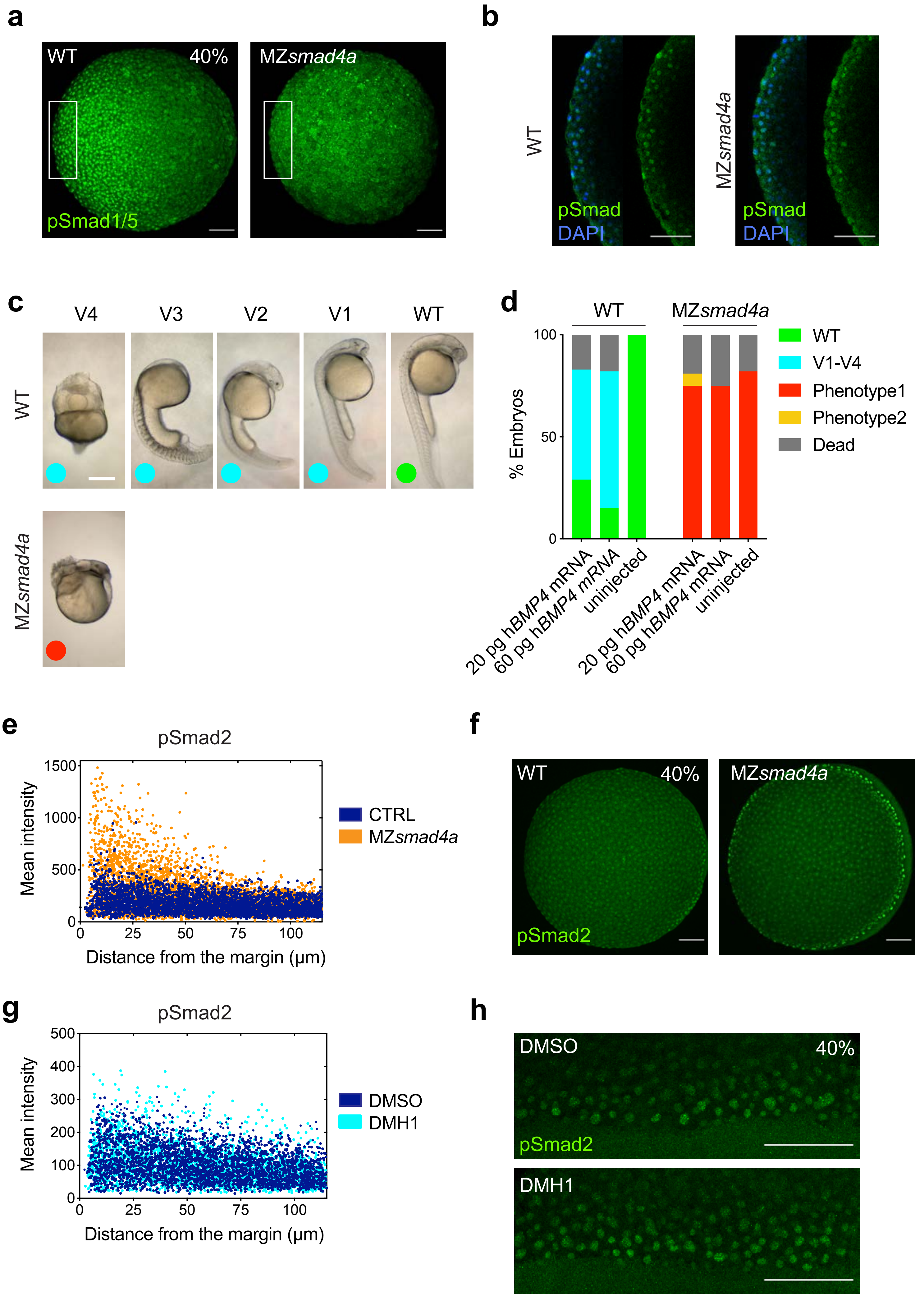

Supplementary Fig. 5

Supplementary Fig. 5. Loss of BMP signaling does not affect Nodal activity in the embryo. a MIP showing immunostaining for pSmad1/5 in WT and MZ*smad4a* embryos at 40% epiboly. Scale bars correspond to 100 µm. Immunostaining is representative of 3 embryos and 2 independent experiments **b** Enlargement of white panels in **a** for WT and MZ*smad4a* mutant embryos. Nuclei were stained with DAPI (blue). Note the pSmad1/5-positive nuclei. Scale bars correspond to 80 µm**. c** Representative images of WT and MZ*smad4a* embryos injected with h*BMP4* mRNA. Colored dots refer to the different phenotypes. Scale bar corresponds to 300 µm (**d**). **d** Quantitation of WT and MZ*smad4a* embryos injected with either 20 or 60 pg of h*BMP4* mRNA. Colors refer to phenotypes in Supplementary Fig. 1j. **e** Raw data related to Figs 4h, i; orange and dark blue dots indicate segmented cell intensities from CTRL and MZ*smad4a* embryos. n=8 embryos for each group. **f** MIP showing immunostaining for pSmad2 in WT and MZ*smad4a* embryos at 40% epiboly. Scale bars correspond to 100 µm. Immunostaining is representative of 6 embryos and 3 independent experiments**. g** Quantitation of **h** where dark blue and cyan dots show segmented cells from DMSO- and DMH1-treated embryos. n=8 embryos for each group. **h** MIP showing pSmad2 immunostaining at the margin of WT embryos treated with DMSO or 10 µM DMH1. Scale bar corresponds to 100 µm.

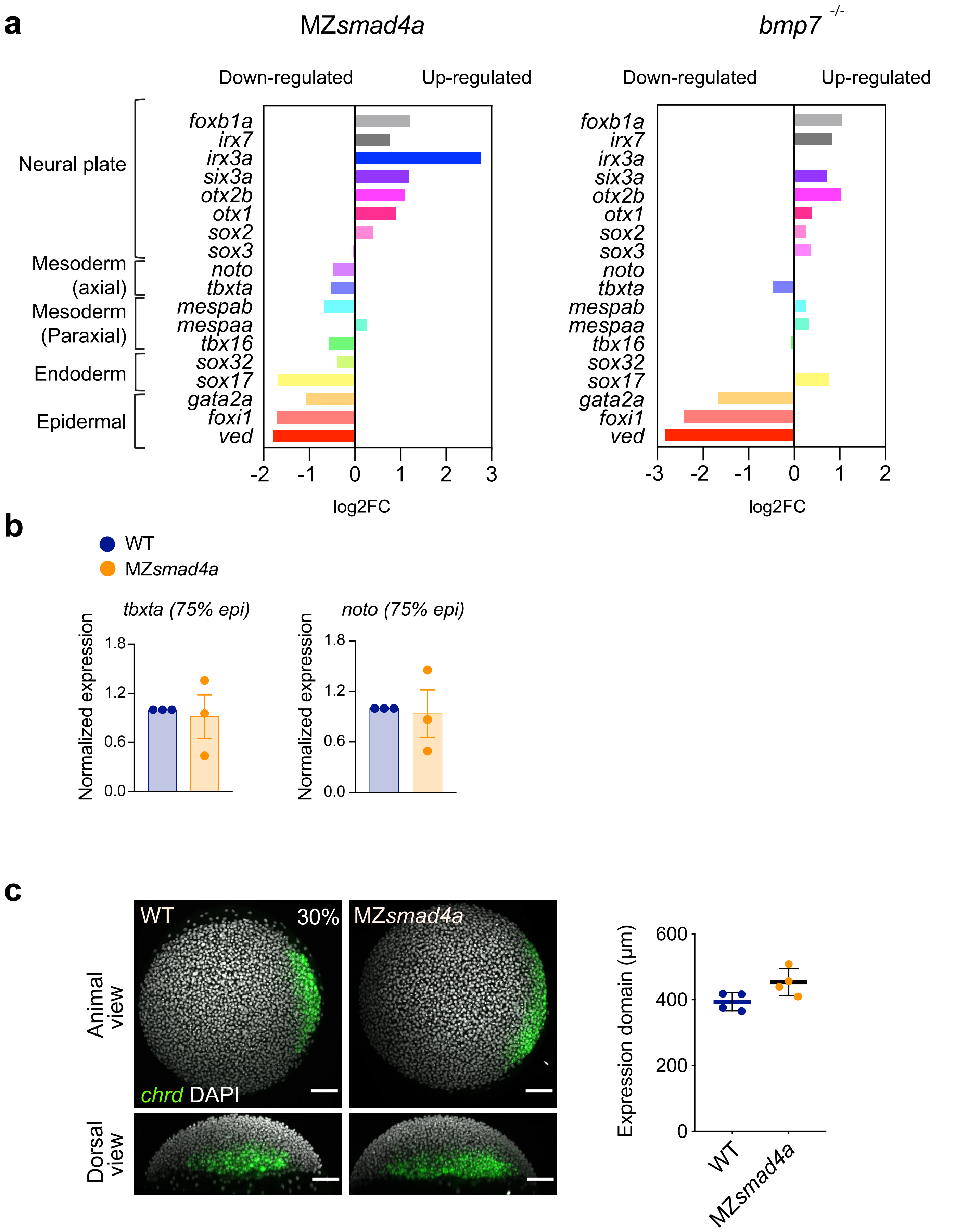

Supplementary Fig. 6

Supplementary Fig. 6. Loss of BMP signaling dominates effects on germ layer formation and morphogenesis in MZ*smad4a* embryos. a RNA-seq data (log2FC) for germ layers markers in MZ*smad4a* (left panel) and *bmp7*$^{-/-}$ (right panel). Data are expressed as log2FC, obtained by the comparison of mutant embryos replicates with their respective WT controls. The tissues are indicated, as are the specific markers. **b** qPCR for *tbxta* and *noto* mRNA in WT and MZ*smad4a* embryos at 75% epiboly. Normalized values are showed as means ± SEM from 3 biological replicates. *tbxta*: p = 0.780, *noto*: p = 0.843, Two-sided one sample t and Wilcoxon test. **c** FISH for *chrd* at 40% epiboly. Upper panel animal view; bottom panel dorsal view. Nuclei were stained with DAPI (white). Right panel shows quantitation of *chrd* expression in WT and MZ*smad4a* embryos. Means ± SD are shown from n=4 embryos for each group. p = 0.114, Two-sided Mann Whitney test. Scale bar corresponds to 100 μm.

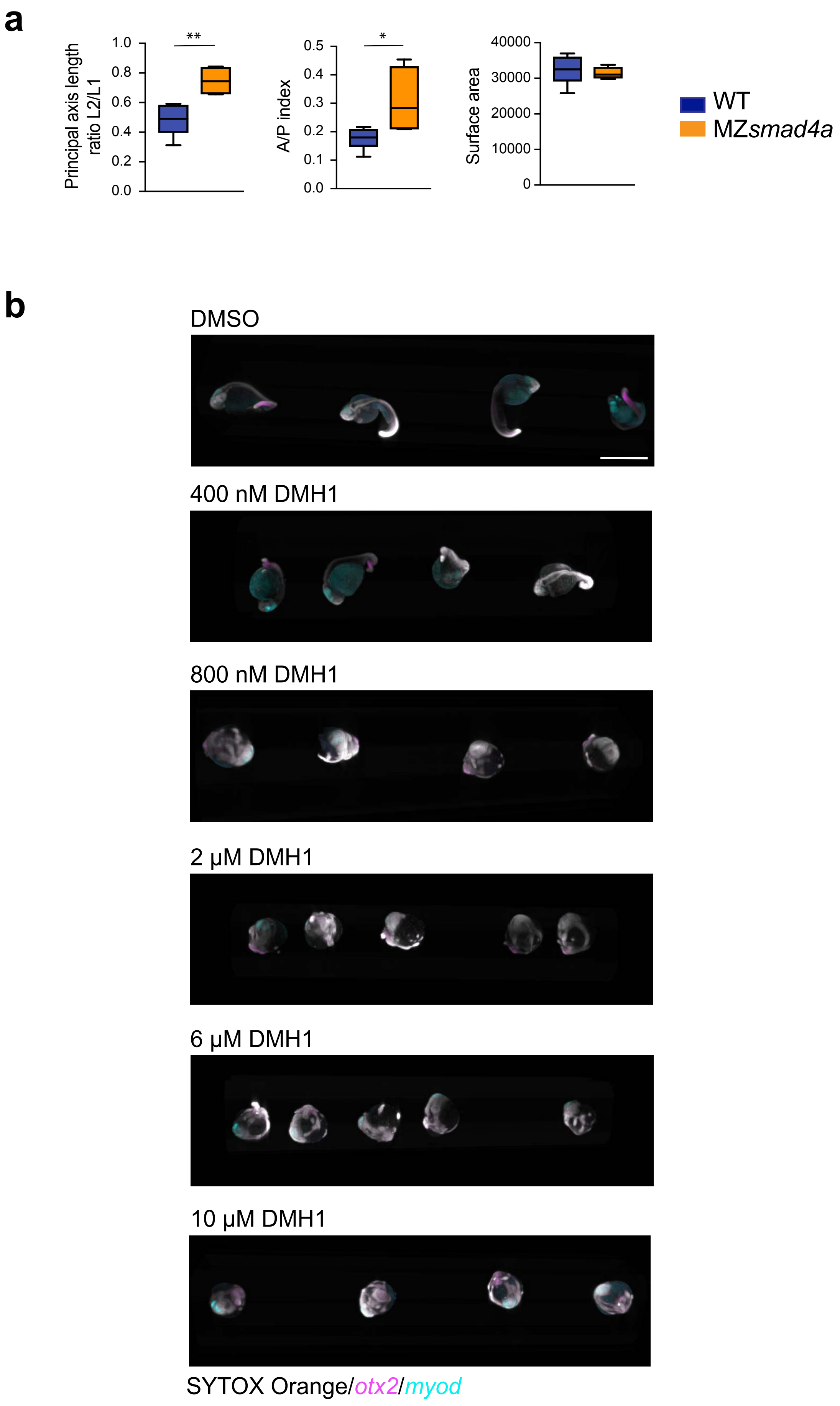

Supplementary Fig. 7

Supplementary Fig. 7. OPT images for embryos treated with different doses of DMH1. a Subset of morphological descriptors from the output array (3 out of 26). Note that all measures, beside the A/P index, refer to the segmented embryo mask. Box-and-whiskers plots show WT (n=6) vs MZ*smad4a* mutants (n=4). Box at 25–75th percentile, whiskers at minimum and maximum values, line at median. Principal axis length ratio L2/L1 p = 0.009, AP index p = 0.038 *, p<0.05; **, p<0.01, Two-sided Mann Whitney test. See also Fig. 6 and Supplementary Fig. 8. **b** Rendering of embryos treated with DMSO or different doses of DMH1 as indicated. Embryos were stained for *otx2* (cyan; anterior marker) and *myod* (magenta; posterior marker). Nuclei were labeled with SYTOX Orange. Images were acquired using OPT. Scale bar corresponds to 975 µm. The WT (DMSO) data shown are the same as in Supplementary Movie 3 for comparison.

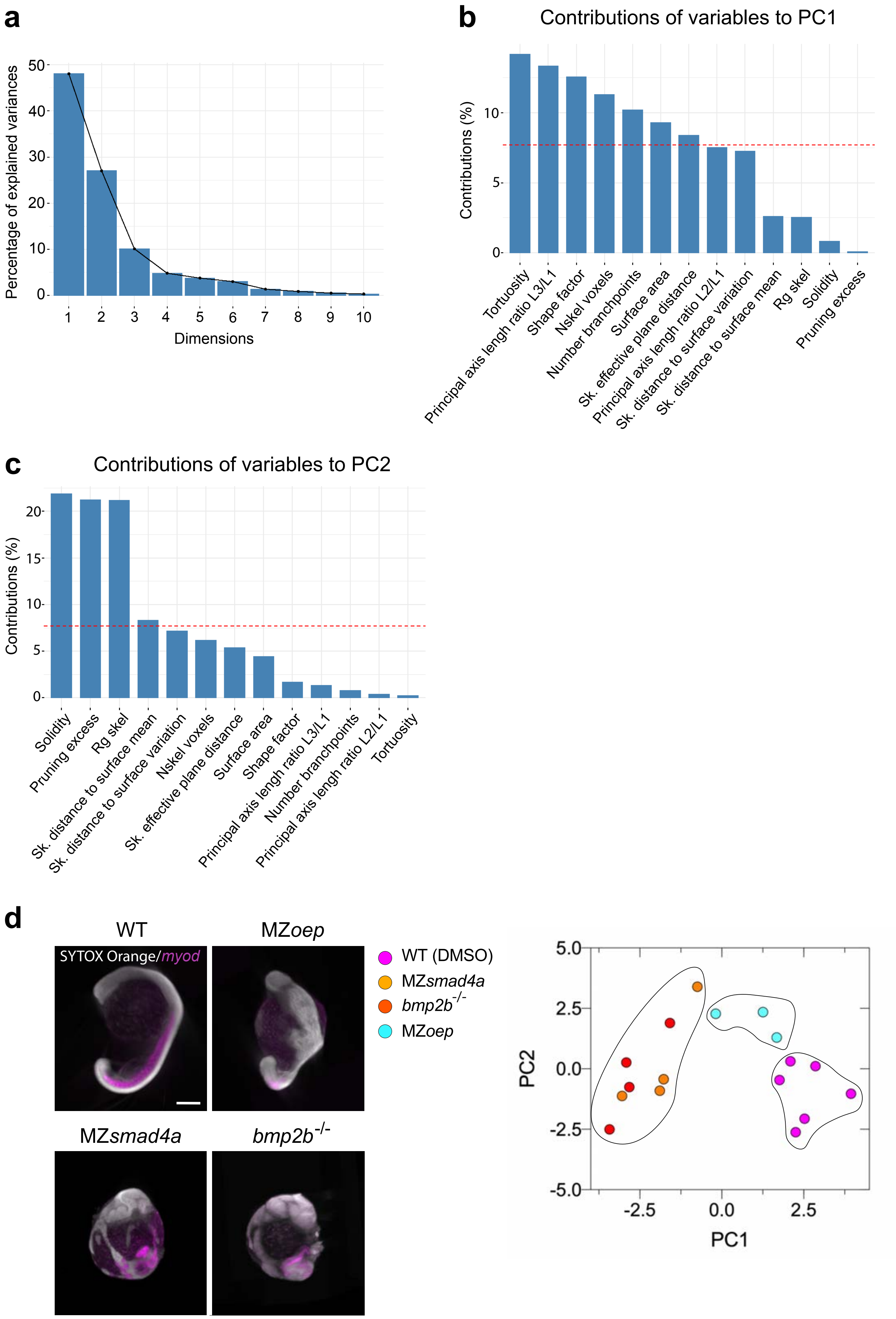

Supplementary Fig. 8

Supplementary Fig. 8. Defining BMP morphospace. a Scree plot showing the contribution of the different Principal Components (PCs) to the total explained variance. **b** Bar plot showing the contribution of the different variables to the PC1 axes. Red dotted line indicates the expected value if the contribution where uniform. **c** Bar plot showing the contribution of the different variables to the PC2 axes. Red dotted line indicates the expected value if the contribution where uniform. **d** Rendering of 3D OPT image data of WT and MZ*smad4a* embryos compared to Nodal (MZ*oep*) and BMP (*bmp2b*$^{-/-}$) mutants. Staining shows *myod* expression (magenta). Scale bar corresponds to 195 µm. Right panel shows MZ*oep* embryos falling on a different region of the morphospace compared to WT, MZ*smad4a* and *bmp2b*$^{-/-}$ mutants.

Supplementary Table I - Oligonucleotides

Gene target	Purpose	Oligonucleotide
**Zebrafish primers**		
smad4a	CRISPR/Cas9 gRNA Fwd oligonucleotide	taggCGGCGCGGGACGGCGGGTGC
	CRISPR/Cas9 gRNA Rev oligonucleotide	aaacGCACCCGCCGTCCCGCGCCG
smad4a	PCR, forward primer (genotyping)	TCAGGGCTGATGGTGAAGGAT
	PCR, reverse primer (genotyping)	TGCTAACATTTGAGCGATGCGT
smad4a	Sequencing primer (genotyping)	GATGCTGGAGAAGGCGGAG
smad4a	PCR, forward primer (cloning)	CGCGatcgatCATTCACAGTACACAGAACAGTGC
	PCR, reverse primer (cloning)	GCGCctcgagTGTCGTGACCTCTGACCCTG
smad4b	PCR, forward primer	CGCGggatccTGAAGCTCGGGAGGATCGAA
	PCR, reverse primer	GCGCctcgagCACATGAATCCGCCCCTGTG
tbx16	PCR forward primer (probe synthesis)	GATCCCGAGCGAGTTGCAAAG
	PCR reverse primer (probe synthesis)	TAATACGACTCACTATAGGGGTACCGGGCGAC TTCGTGTG
lft2	qPCR, forward primer	GGACATGGGCGCACCAGAACT
	qPCR, reverse primer	TACCCGGCCGGCTCGATGAT
lft1	qPCR, reverse primer	GCTGGTGCTTTACACACTCAACCT
	qPCR, reverse primer	GTTCCCTGCAGCACATTTCACG
eve1	qPCR, forward primer	ATGCCAGAGGACAGGGAGTT
	qPCR, reverse primer	GAGTCAGCTGTTCCCTCGTG
dlx3b	qPCR, forward primer	GATGGGGGCAACACCCGGAG
	qPCR, reverse primer	TGTGGTGGCGTCTGCAGGTC
ndr1	qPCR, forward primer	CTCCGTCTTGAGCCTCGTCG
	qPCR, reverse primer	TCGCTGGACGTCATCGCTTG
ndr2	qPCR, forward primer	AATGCATACCGGTGCGAGGG
	qPCR, reverse primer	GCAGGAACACGACTGGGGTG
noto	qPCR, forward primer	CTGTTGGCATCTGCTCTCCA
	qPCR, reverse primer	TCTCCATTTGATGCGCCTGT

tbxta	qPCR, forward primer	AAGACGCGGAGTTGTGGACC
	qPCR, reverse primer	ACTGGCTCTGAGCACGGGAA
smad4a	qPCR, forward primer	CGGATCTGTCGGAGGAATCG
	qPCR, reverse primer	TCTTTAATGCTCTGCCGGGG
smad4b	qPCR, forward primer	CGCCTGACACTTTCAACAGC
	qPCR, reverse primer	AGGAATGCTGGGAGGAAAGC
actin	qPCR, forward primer	CGAGCTGTCTTCCCATCCA
	qPCR, reverse primer	TCACCAACGTAGCTGTCTTTCTG
**Human primers**		
ID3	Top *ID3* WT (DNA pulldown assay)	5'biotin-TTGATCTAAGACTCAGCCCCAGACAGCCTGGCGCCAGGGTGGGACGTCA
	Bottom *ID3* WT (DNA pulldown assay)	TGACGTCCCACCCTGGCGCCAGGCTGTCTGGGGCTGAGTCTTAGATCAA
ID3	Top *ID3* Mut (DNA pulldown assay)	5' biotin-TTGATCTAATATTCAGCCCCATATAGCCTATATATAGGGTGGGACGTCA
	Bottom *ID3* Mut (DNA pulldown assay)	TGACGTCCCACCCTATATATAGGCTATATGGGGCTGAATATTAGATCAA
	Top mutated competitive oligonucleotide (DNA pulldown assay)	GGATTTGCTAATGATATAGTAATATATATATATACATATATATATATTGATCTTCA
	Bottom mutated competitive oligonucleotide (DNA pulldown assay)	TGAAGATCAATATATATATATATGTATATATATATATTACTATATCATTAGCAAATCC
BMP4	PCR, forward primer (cloning)	CGCGggatccTTTCTGTCAAGACACCATGATTCC
	PCR, reverse primer (cloning)	GCGCctcgagTATCCTCAAGGACTGCCTGATCT
**Mouse primers**		
Smad4	CRISPR/Cas9 gRNA Fwd oligonucleotide	CACCGACAACCCGCTCATAGTGATA
	CRISPR/Cas9 gRNA Rev oligonucleotide	AAACTATCACTATGAGCGGGTTGTC
Smad4	*Smad4* Forward primer for clone screening	GGAGCTGTAGCCTTCAGTTAGCT

	Smad4 Reverse primer for clone screening	AGGCACGCTGGTGTATGTCTCTA
Gapdh	qPCR, forward primer	TCTTGTGCAGTCCCAGCCT
	qPCR, reverse primer	CAATATGGCCAAATCCGTTCA
Lefty1	qPCR, forward primer	GCTCGATCAACCGCCAGTCCTG
	qPCR, reverse primer	GCCACCTCTCGAAGGTTCTGGCT
Lefty2	qPCR, forward primer	AGAGATGACCTCCTTGCCCA
	qPCR, reverse primer	ACAGATCTATCCCCCTGGGT
T	qPCR, forward primer	TGTGACCAAGAACGGCAGGAGG
	qPCR, reverse primer	CAGCGGTGGTTGTCAGCCGT
Id1	qPCR, forward primer	GCGAGATCAGTGCCTTGG
	qPCR, reverse primer	CTCCTGAAGGGCTGGAGTC
Id2	qPCR, forward primer	GCAGATCGCCCTGGACTCGC
	qPCR, reverse primer	CAGATGCCTGCAAGGACAGGATGC
Id3	qPCR, forward primer	CCTCCCGAACGCAGGTGCTG
	qPCR, reverse primer	CATGCCCTCAGGCTTCCGGC

SUPPLEMENTARY TABLE 2 - KEY RESOURCES

REAGENT or RESOURCE	SOURCE	IDENTIFIER
**Antibodies**		
Anti-phospho-Smad2 (IF) (Dilution: 1 in 800)	Cell Signaling Technology	Cat# 8828; RRID: AB_2631089
Anti-phospho-Smad2 (WB) (Dilution: 1 in 250)	Cell Signaling Technology	Cat# 3108 RRID: AB_490941
Anti-phospho-Smad1/5 (IF, WB) (Dilution: 1 in 800 (IF); 1 in 1000 (WB))	Cell Signaling Technology	Cat# 13820; RRID:AB_2493181
Anti-SMAD2/3 [clone 18] (WB) (Dilution: 1 in 1000)	BD Biosciences	Cat#610842; RRID: AB_398161
Anti-SMAD4 [B-8] (WB) (Dilution: 1 in 500)	Santa Cruz Biotechnology	Cat# sc-7966 RRID:AB_627905
Anti-SMAD5 (WB) (Dilution: 1 in 2000)	Proteintech	Cat# 12167-1-AP RRID:AB_2286502
Anti-MCM6 [H-8] (WB) (Dilution: 1 in 2000)	Santa Cruz Biotechnology	Cat# sc-393618 RRID:AB_2885187
Anti-Tubulin [YL1/2] (WB) (Dilution: 1 in 10,000)	Abcam	Cat# ab6160 RRID:AB_305328
Anti-Digoxigenin HRP (*in situ*) (Dilution: 1 in 500)	Roche	Cat# 1207733910
Anti-DNP AP (*in situ*) (Dilution: 1 in 1000)	Vector Laboratories	Cat# MB-3100; RRID: AB_2336089
Anti-Rabbit Alexa Fluor 488 (IF) (Dilution: 1 in 400)	ThermoFisher Scientific	Cat# A-21206; RRID: AB_2535792
Goat anti-rabbit HRP (WB) (Dilution: 1 in 5000)	Dako	Cat# P0448 RRID: AB_2617138
Goat anti-mouse HRP (WB) (Dilution: 1 in 5000)	Dako	Cat# P0447 RRID:AB_2617137
Rabbit anti-rat HRP (WB) (Dilution: 1 in 5000)	Dako	Cat# P0450 RRID:AB_2630354
**Chemicals, Peptides, and Recombinant Proteins**		
SB-505124	Tocris	Cat# S4696
DMH1	Selleck Chemicals	Cat# S7146
Fast Red TR/Naphthol AS-MX Tablets	Sigma	Cat# F4648
Tyramine hydrochloride	Sigma	Cat# T2879
NHS-Fluorescein ester	ThermoFisher Scientific	Cat# 46410
Fast Blue BB Salt	Sigma	Cat# F3378
Naphthol AS-MX phosphate disodium salt (NAMP)	Sigma	Cat# N5000
Pierce NeutrAvidin Agarose (DNAP and peptide pulldown assay)	ThermoFisher Scientific	Cat# 29200
Human recombinant BMP4	Peprotech	Cat# 120-05ET
Human recombinant Activin A	Peprotech	Cat# 120-14E
DMEM/F-12	ThermoFisher Scientific	Cat# 10565018
Digoxigenin (Dig)-11-UTP	Roche	Cat# 11209256910
dinitrophenol (DNP)-11-UTP	Perkin Elmer	Cat# NEL555001EA
DAPI	Sigma Aldrich	Cat# 10236276001
TRIzol	ThermoFisher Scientific	Cat# 15596026
MO-*smad4b* GACATGCTGCTGGATTTACACTCAA	Gene Tools, LLC	www.gene tools.com
mpMO-smad4b GAGATCCTGCTCGATTAAGACTCAA	Gene Tools, LLC	www.gene tools.com
**Critical Commercial Assays**		
Multiplex Fluorescent Assay v2	ACDBio	acdbio.com
Power-Up SYBR Green Master Mix	ThermoFisher Scientific	Cat# A25742

**Deposited Data**		
Bulk RNA-seq raw data. Accession number: GSE162289	Gene Expression Omnibus	https://www.ncbi.nlm.nih.gov/geo/query/acc.cgi?acc=GSE162289
Sc-RNA-seq raw data. Accession number: GSE164574	Gene Expression Omnibus	https://www.ncbi.nlm.nih.gov/geo/query/acc.cgi?acc=GSE164574
**Experimental Models: Cell Lines**		
HaCaT cells, human	Francis Crick Institute Cell Services	
HaCaT SMAD4 KO, human	Gori et al., 2021 PMID: 33416497	
E14-TG2a cells carrying a *Hhex*-RedStar/*Gsc*-GFP, mouse	Villegas et al., 2013 PMID: 24368729	
E14-TG2a cells carrying a *Hhex*-RedStar/*Gsc*-GFP SMAD4 KO, mouse	This paper	
**Experimental Models: Organisms/Strains**		
Zebrafish *Danio rerio*: WT		
Zebrafish *Danio rerio*: MZ*smad4a*$^{\Delta 14}$, M*smad4a*$^{\Delta 14}$, Z*smad4a*$^{\Delta 14}$	This Paper	
Zebrafish *Danio rerio*: *bmp2b*^{tdc24}*(swirl)*	Hammond et al., 2009 PMID: 19190757	
Zebrafish *Danio rerio*: MZ*oep*	Gritsman et al, 1999 PMID: 10199408	
**Oligonucleotides**		
For the list of oligonucleotides used see Supplementary Table 1		
**Recombinant DNA**		
pCS2-hBMP4: for mRNA digest NotI, transcribe SP6	This Paper	N/A
pCS2-zf*smad4b*: for mRNA digest NotI, transcribe SP6	This Paper	N/A
pCS2-zf*smad4a*: for mRNA digest ApaI, transcribe SP6	This Paper	N/A
pCS2-H2B_PSmOrange: for mRNA synthesis linearize with NotI: polymerase SP6	Beretta et al., 2013 PMID: 24067090	N/A
pFTX5-HSmad4: for mRNA synthesis linearize with: XbaI; polymerase T7	Howell et al., 1999 PMID: 10525340	N/A
pT7-gRNA for mRNA synthesis linearize with: BamH1; polymerase T7	Jao et al., 2013 PMID: 23918387	N/A
pT3TS-nls-zCas9-nls for mRNA synthesis linearize with: XbaI; polymerase T3	Jao et al., 2013 PMID: 23918387	N/A
pSpCas9(BB)−2A-GFP (PX458)	Ran et al 2013 PMID: 24157548	N/A
**Software and Algorithms**		
FIJI (ImageJ)	Schneider et al., 2012 PMID: 22930834	https://imagej.net/Fiji/Downloads
Imaris	Oxford instruments	https://imaris.oxinst.com/
Icy	Institut Pasteur and France-Bioimaging	http://icy.bioimageanalysis.org/
Matlab	MathWorks	https://www.mathworks.com/products/matlab.html
R	The R Foundation	https://www.r-project.org/

Prism	GraphPad	https://www.graphpad.com/scientific-software/prism/
**Other**		
Dr-*lft1* probe (RNAscope)	ACDBio	Cat# 557771-C2
Dr-*ndr1* probe (RNAscope)	ACDBio	Cat# 557761-C1
Antisense RNA probe *tbxta*: linearize Xho1: polymerase T7	Schulte-Merker et al.,1992<br>PMID: 7600949	N/A
Antisense RNA probe *sox32*: linearize Not1: polymerase T7	https://zfin.org/ZDB-PUB-010810-1<br>ZFIN online publication	N/A
Antisense RNA probe *myod*: linearize Xba1: polymerase T7	Weinberg et al., 1996<br>PMID: 8565839	N/A
Antisense RNA probe *otx2*: linearize EcoRI: polymerase T7	Li et al., 1994<br>PMID: 7893604	N/A
Antisense RNA probe *chrd*: linerize Spe1; polymerase T7	Miller-Bertoglio et al 1997<br>PMID:9441687	N/A
