# Supplementary material for: Smad4 controls signaling robustness and morphogenesis by differentially contributing to the Nodal and BMP pathways
Source: Nat Commun. 2021 Nov 4;12:6374. doi: 10.1038/s41467-021-26486-3 (PMC8569018; doi:10.1038/s41467-021-26486-3)
Supplement: Supplementary file 3 — Description of Additional Supplementary Files [file 41467_2021_26486_MOESM3_ESM.docx]

**Description of Additional Supplementary Files**

**Title: Supplementary Movie 1: Live imaging of WT embryo development**

Description: Rendering of MuVi-SPIM light sheet microscope 3D image data of mosaically-labeled nuclei in WT embryos. PSmOrange mRNA was injected at the 1-cell stage. Acquisition was started at mid gastrulation for 11 consecutive hours with 5 min intervals.

**Title: Supplementary Movie 2: Live imaging of MZ*smad4a* embryo development**

Description: Rendering of MuVi-SPIM light sheet microscope 3D image data of mosaically-labelled nuclei in MZ*smad4a* embryos. PSmOrange mRNA was injected at the 1-cell stage. Acquisition was started at mid gastrulation for 11 consecutive hours with 5 min intervals.

**Title: Supplementary Movie 3: OPT acquisition of fluorescently-labeled WT embryos** Rendering of Description: multi-sample OPT image data of rotating WT embryos at 24 hpf. Embryos were stained for *otx2* (cyan; anterior marker) and *myod* (magenta; posterior marker). Nuclei were labeled with SYTOX Orange.

**Title: Supplementary Movie 4: OPT acquisition of fluorescently-labeled MZ*smad4a* embryos**

Description: Rendering of multi-sample OPT image data of rotating MZ*smad4a* embryos at 24 hpf. Embryos were stained for *otx2* (cyan; anterior marker) and *myod* (magenta; posterior marker). Nuclei were labeled with SYTOX Orange.

**Title: Supplementary Software**

Description: Custom code for embryo segmentation and quantitation implemented in MATLAB, enabling the extraction of morphological parameters, which were used for the analyses shown in Figs 6 and 7 and Supplementary Figs 7 and 8. Information concerning how to run the code can be found in the README.md file in the following [Github link](https://github.com/yalexand/ALYtools).

**Title: Supplementary Dataset 1**

Description: Raw data for the bulk RNA-seq showing normalized RNA-seq reads for three batches of WT and MZ*smad4a* mutant embryos. Means are shown and log2 fold change between the WT and mutant Mean read values are shown, as well as the relevant statistics. These data were used to generate the heatmaps displayed in Fig. 2a, and the graphs in Figs 1h and 2b and Supplementary Figs 1h, 2c, 3b and 6a.

**Title: Supplementary Dataset 2**

Description: A spreadsheet showing the parameters used for the quantitation of segmented embryos (sheet 1), and the output values of the resulting analyses for each embryo (sheet 2). The descriptors in sheet 2 are defined in sheet 1. The descriptors colored in yellow were used for the PCA plots shown in Figs 7b, c and e and in Supplementary Fig 8d.
