# Supplementary figures and images for: Smad4 controls signaling robustness and morphogenesis by differentially contributing to the Nodal and BMP pathways
Source: Nat Commun. 2021 Nov 4;12:6374. doi: 10.1038/s41467-021-26486-3 (PMC8569018; doi:10.1038/s41467-021-26486-3)

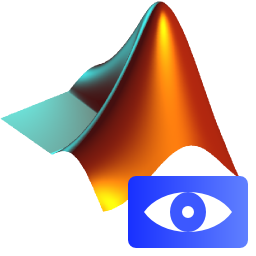

Supplement: Supplementary file 10 — Software code [file 41467_2021_26486_MOESM10_ESM.zip › ALYtools-master 08.21/icy_matlab/matlabxserver/MatlabXServerDeamon.png]

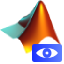

Supplement: Supplementary file 10 — Software code [file 41467_2021_26486_MOESM10_ESM.zip › ALYtools-master 08.21/icy_matlab/matlabxserver/MatlabXServerDeamon_icon.png]

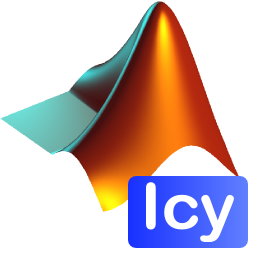

Supplement: Supplementary file 10 — Software code [file 41467_2021_26486_MOESM10_ESM.zip › ALYtools-master 08.21/icy_matlab/matlabcommunicator/MatlabCommunicatorDeamon.png]

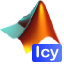

Supplement: Supplementary file 10 — Software code [file 41467_2021_26486_MOESM10_ESM.zip › ALYtools-master 08.21/icy_matlab/matlabcommunicator/MatlabCommunicatorDeamon_icon.png]
