# Supplementary material for: Smad4 controls signaling robustness and morphogenesis by differentially contributing to the Nodal and BMP pathways
Source: Nat Commun. 2021 Nov 4;12:6374. doi: 10.1038/s41467-021-26486-3 (PMC8569018; doi:10.1038/s41467-021-26486-3)
Supplement: Supplementary file 12 — Supplementary Data File 3_ESM [file 41467_2021_26486_MOESM12_ESM.zip › Sphere-scRNA-seq.html]

Sphere stage


# Sphere stage

#### Luca Guglielmi

#### 14/09/2020

# 1: Loading required packages

```
library(dplyr)
```

```
## 
## Attaching package: 'dplyr'
```

```
## The following objects are masked from 'package:stats':
## 
##     filter, lag
```

```
## The following objects are masked from 'package:base':
## 
##     intersect, setdiff, setequal, union
```

```
library(Seurat)
```

```
## Attaching SeuratObject
```

```
library(patchwork)
```

# 2: Loading the dataset via the Read10X function

```
sphere2<- Read10X(data.dir = "~/Desktop/GSM5014336_Zsphere_scRNA")
```

# 3: Transforming the matrix into a Seurat Object

```
sphere2 <- CreateSeuratObject(counts = sphere2, project = "sphere2", min.cells = 3, min.features = 200)
```

```
## Warning: Feature names cannot have underscores ('_'), replacing with dashes
## ('-')
```

# 4: Calculating percentage of mitochondrial genes

```
sphere2 [["percent.mt"]] <- PercentageFeatureSet(sphere2, pattern = "^mt-")
```

# 5: Visualizing QC metrics

```
VlnPlot(sphere2, features = c("nFeature_RNA", "nCount_RNA", "percent.mt"), ncol = 3)
```

# 6: We remove dying cells using the subset function

```
sphere2 <- subset(sphere2, subset = percent.mt < 10)
```

# 7: We normalize the data by using the Log Normalize function

```
sphere2 <- NormalizeData(sphere2, normalization.method = "LogNormalize", scale.factor = 10000)
```

# 8: Identification of highly variable features

```
sphere2 <- FindVariableFeatures(sphere2, selection.method = "vst", nfeatures = 2000)
top10 <- head(VariableFeatures(sphere2), 10)
plot1 <- VariableFeaturePlot(sphere2)
plot1 <- LabelPoints(plot = plot1, points = top10, repel = TRUE)
```

```
## When using repel, set xnudge and ynudge to 0 for optimal results
```

```
plot1
```

# 9: Scaling the data

```
all.genes <- rownames(sphere2)
```

```
sphere2 <- ScaleData(sphere2, features = all.genes)
```

```
## Centering and scaling data matrix
```

# 10: Perform linear dimensional reduction (PCA)

```
sphere2 <- RunPCA(sphere2, features = VariableFeatures(object = sphere2))
```

```
## PC_ 1 
## Positive:  cldne, cebpb, krt4, krt18, si:ch211-125o16.4, grhl3, cldnb, ackr3b, mid1ip1a, krt8 
##     ftr82, plekhf1, klf2a, lye, rassf7b, gadd45bb, her6, si:ch211-95j8.2, ovol1, gata3 
##     neurl1ab, gadd45ba, arl13a, gata2a, stard14, fzd7a, tfap2a, grhl1, depdc7, CYSTM1 (1 of many) 
## Negative:  zic2b, si:ch1073-80i24.3, her5, cx43.4, fam212aa, cxcr4a, fam46ba, aldob, zgc:113424, sb:cb81 
##     id1, has2, ccna1, si:rp71-45k5.2, apoeb, admp, mych, mex3b, anp32a, si:dkeyp-87d1.1 
##     zgc:64022, ccnb2, snai2, gmnn, cited2, nnr, blf, tbx16, BX324155.1, zic3 
## PC_ 2 
## Positive:  ccnd1, itpkcb, nnr, exd2, zgc:64022, CR383676.1, si:ch73-1a9.3, abca3b, thy1, tacc3 
##     fbxo5, admp, si:dkeyp-115e12.6, aspm, sox3, znf1046, sox19a, si:ch211-57f7.7, cldnd, svila 
##     fhdc3, ccdc102a, fam212aa, cx43.4, id1, gpd1b, sult6b1, cited2, si:dkey-122c11.8, per1b 
## Negative:  mixl1, noto, foxa3, ca15b, sebox, wnt11, fgf8a, myl12.1, arl4ab, lamp2 
##     chd, lft1, dharma, ta, vrtn, ddx4, mid1ip1l, CABZ01090031.1, rasgef1ba, arg2 
##     dazl, acp5a, dkk1b, si:dkey-261j4.3, siva1, ndel1b, osr1, flrt3, celf1, pfn2 
## PC_ 3 
## Positive:  mixl1, fgf8a, noto, wnt11, dharma, arl4ab, foxa3, ta, lft1, sebox 
##     osr1, chd, flrt3, dkk1b, ism1, CR383676.1, lft2, fgf4, nog1, tbx16 
##     fgf17, pnrc2, znfl2a, fzd8b, si:dkey-261j4.3, si:dkey-238d18.15, foxa2, vrtn, ndr1, pitx2 
## Negative:  ccnb1, cldnd, thy1, zgc:64022, sox19b, cth1, epcam, aurka, id1, tpx2 
##     ccnb2, zgc:113424, si:dkeyp-115e12.6, zgc:55733, stmn1a, zgc:153867, ccna1, bora, sinup, pias4b 
##     ctss2.1, ube2ib, arntl2, pttg1, zgc:56493, sox3, elovl7b, fam212aa, orc6, tacc3 
## PC_ 4 
## Positive:  CABZ01090031.1, phlda3, si:ch211-239d6.2, zgc:114130, isg15, mid1ip1l, gadd45aa, mdm2, bbc3, sesn1 
##     rnd3a, zgc:158852, ccnb1, foxo3b, sox19b, ccna1, rasl11b, h1m, aplnrb, cxcl12b 
##     cdkn1a, cldng, cth1, acp5a, grasp, zgc:162879, spsb1, vps9d1, si:ch211-260e23.9, si:dkey-85n7.7 
## Negative:  asb11, tbx16, CR383676.1, hnrnpa0b, ccnd1, marcksl1b, si:ch1073-80i24.3, sb:cb81, cox7c, stm 
##     vgll4l, hspb1, lrwd1, dynll1, aldob, blf, si:ch211-173m16.2, zic2b, tdgf1, pnrc2 
##     RPL41, sox11a, nnr, hmgn6, kpna2, hmgb2a, polr3gla, znfl2a, si:ch73-1a9.3, yy1a 
## PC_ 5 
## Positive:  CABZ01090031.1, zgc:114130, mid1ip1l, sox19b, ccna1, id1, zgc:158852, ccnb1, cxcr4a, zgc:77118 
##     ccnb2, dusp6, siva1, h1m, p4hb, btg3, zgc:113424, zic2a, gmnn, lsm14b 
##     acp5a, cth1, lsm4, ube2al, brf2, snupn, rasgef1ba, ccnd1, klf17, rhpn2 
## Negative:  si:ch211-239d6.2, phlda3, isg15, gadd45aa, foxo3b, sesn3, aplnrb, fosab, mdm2, cxcl12b 
##     cdkn1a, rasl11b, bbc3, lnx1, RPS27L, bdnf, rnd3a, sesn1, tp53inp1, foxi1 
##     si:dkey-85n7.7, rbl2, pimr191, METRNL (1 of many), RNF169, si:ch211-260e23.9, grasp, tat, phlda2, ctsh
```

```
DimPlot(sphere2, reduction = "pca")
```

# 11: Determine dataset dimentionality

```
ElbowPlot(sphere2)
```

The majority of true signal is captured within the first 15 PCs

# 12: Clustering the cells

```
sphere2 <- FindNeighbors(sphere2, dims = 1:15)
```

```
## Computing nearest neighbor graph
```

```
## Computing SNN
```

```
sphere2 <- FindClusters(sphere2, resolution = 0.2)
```

```
## Modularity Optimizer version 1.3.0 by Ludo Waltman and Nees Jan van Eck
## 
## Number of nodes: 2595
## Number of edges: 85814
## 
## Running Louvain algorithm...
## Maximum modularity in 10 random starts: 0.8481
## Number of communities: 4
## Elapsed time: 0 seconds
```

we use a low resolution for the Louvain algorithm to have an Idea of the general regionalization of the emrbyo

# 13: Non-linear dimensional reduction (uMAP)

```
sphere2 <- RunUMAP(sphere2, dims = 1:15)
```

```
## Warning: The default method for RunUMAP has changed from calling Python UMAP via reticulate to the R-native UWOT using the cosine metric
## To use Python UMAP via reticulate, set umap.method to 'umap-learn' and metric to 'correlation'
## This message will be shown once per session
```

```
## 17:28:13 UMAP embedding parameters a = 0.9922 b = 1.112
```

```
## 17:28:13 Read 2595 rows and found 15 numeric columns
```

```
## 17:28:13 Using Annoy for neighbor search, n_neighbors = 30
```

```
## 17:28:13 Building Annoy index with metric = cosine, n_trees = 50
```

```
## 0%   10   20   30   40   50   60   70   80   90   100%
```

```
## [----|----|----|----|----|----|----|----|----|----|
```

```
## **************************************************|
## 17:28:13 Writing NN index file to temp file /var/folders/s2/phwgmcs926x8kbjk57m5lqw14_t3jy/T//Rtmp4q5BjM/file32203e95a44f
## 17:28:13 Searching Annoy index using 1 thread, search_k = 3000
## 17:28:14 Annoy recall = 100%
## 17:28:14 Commencing smooth kNN distance calibration using 1 thread
## 17:28:14 Initializing from normalized Laplacian + noise
## 17:28:14 Commencing optimization for 500 epochs, with 99410 positive edges
## 17:28:17 Optimization finished
```

```
DimPlot(sphere2, reduction = "umap",label = TRUE,  pt.size = 2) + NoLegend()
```

# 14: Plotting expression of selected genes

```
FeaturePlot(sphere2, features = c("smad4a"), pt.size = 2)
```

# 15: Plotting percentage of mitocondrial genes on the uMAP

```
FeaturePlot(sphere2, features = c("percent.mt"), pt.size = 2)
```

# 16: Plotting percentage of mitocondrial genes across clusters using the VlnPlot function

```
VlnPlot(sphere2, features = c("percent.mt"), ncol = 3)
```

# 17: Characterizing the different clusters (20 genes each)

```
sphere2.markers <- FindAllMarkers(sphere2, only.pos = TRUE, min.pct = 0.25, logfc.threshold = 0.25)
```

```
## Calculating cluster 0
```

```
## For a more efficient implementation of the Wilcoxon Rank Sum Test,
## (default method for FindMarkers) please install the limma package
## --------------------------------------------
## install.packages('BiocManager')
## BiocManager::install('limma')
## --------------------------------------------
## After installation of limma, Seurat will automatically use the more 
## efficient implementation (no further action necessary).
## This message will be shown once per session
```

```
## Calculating cluster 1
```

```
## Calculating cluster 2
```

```
## Calculating cluster 3
```

```
sphere2.markers %>%  group_by(cluster) %>%  top_n(n = 20, wt = avg_log2FC) -> top20
DoHeatmap(sphere2, features = top20$gene) + NoLegend()
```

# 18:Plotting expression of cluster specific genes

```
FeaturePlot(sphere2, features = c("zic3","zic2b","admp","aldob","zic2b","id1"), pt.size = 1)
```

```
FeaturePlot(sphere2, features = c("cebpb","lye","grhl3","cldnb"), pt.size = 1)
```

```
FeaturePlot(sphere2, features = c("lft1","dharma","mixl1","chd"), pt.size = 1)
```

```
FeaturePlot(sphere2, features = c("sesn3","mdm2"), pt.size = 1)
```

# 19:Plotting expression of signalling effectors

```
FeaturePlot(sphere2, features = c("bmp7a","bmp2b","bmp4","smad2","smad1","smad5","smad2","smad3a","smad3b"), pt.size = 1)
```
