# Supplementary material for: Smad4 controls signaling robustness and morphogenesis by differentially contributing to the Nodal and BMP pathways
Source: Nat Commun. 2021 Nov 4;12:6374. doi: 10.1038/s41467-021-26486-3 (PMC8569018; doi:10.1038/s41467-021-26486-3)
Supplement: Supplementary file 10 — Software code [file 41467_2021_26486_MOESM10_ESM.zip › ALYtools-master 08.21/SIFNE/SIFNE_User-Manual.pdf]

© 2016 Kanchanawong Laboratory, Mechanobiology Institute, National University of Singapore

SIFNE 1.0

User Manual

Content

1. Preparation 3
    1.1 Source File 3
    1.2 Software Installation 3
    1.3 Data Directory 3
2. Data Processing Steps 4
    2.1 Load Image 4
    2.2 Image Enhancement 4
        2.2.1 Preview and Choose Region of Interest 4
        2.2.2 Line and Orientation Filter Transform 6
    2.3 Segmentation and Tip Registration 6
        2.3.1 Segmentation 6
        2.3.2 Junction Removal 7
        2.3.3 Iterative Extraction of Linear Fragments 7
        2.3.4 Tip Registration 8
    2.4 Grouping and Analysis 8
        2.4.1 Image Information 8
        2.4.2 Preview and Search Criteria 9
        2.4.3 Tip Pairing and Grouping 11
        2.4.4 Filament Sorting 11
        2.4.5 Analysis Features 11
            2.4.5.1 Detected Filament 12
            2.4.5.2 Junctions 12
            2.4.5.3 Filament Orientation 13
            2.4.5.4 Curvature 13
            2.4.5.5 Export into Excel 14
3. Manual Correction 17
    3.1 Initialization 17
    3.2 Connect 19
    3.3 Break/Remove 19
    3.4 New Filament 19
    3.5 Continue 19
    3.6 Save 19

1. Preparation

1.1 Source File

Download the software package named *SIFNE.zip* and unzip it (Fig. 1). The unzipped folder contains the following sample images for testing.
(1) *spider.tiff*. Synthetic image of cobweb patterin for quick test.
(2) *MT.tiff*. Superresolution image of 4088×4088 pixels with pixel size of 20nm.
** This manual uses MT.tiff for illustration. Parameters can be used for spider.tiff as well, unless otherwise mentioned.*

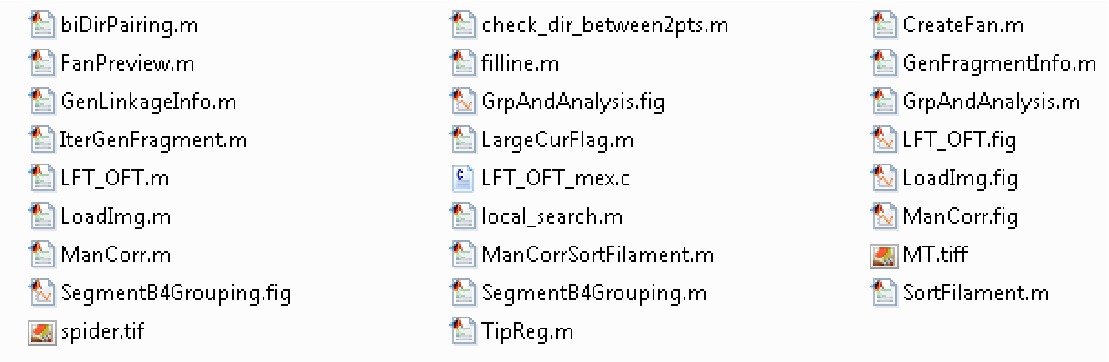

Figure 1. Files included with SIFNE 1.0 package

There are 4 user interfaces (GUIs) that are sequentially opened.
(1) *LoadImg.m*/*LoadImg.fig*
(2) *LFT_OFT.m*/*LFT_OFT.fig*
(3) *SegmentB4Grouping.m*/*SegmentB4Grouping.fig*
(4) *GrpAndAnalysis.m*/*GrpAndAnalysis.fig*

1.2 Software Installation

In SIFNE, most code were written using Matlab except for one routine for image enhancement which is written in C, *LFT_OFT_mex.c*. In order to call this external C function, the user needs to create a MEX file by setting up a C compiler to compile this C code via command '*mex -setup*'. A list of supported and compatible compilers can be found in the following link.
http://www.mathworks.com/support/sysreq/files/SystemRequirements-Release2015a_SupportedCompilers.pdf
Upon successful compilation, the user should be able to see a new file (e.g. *LFT_OFT_mex.mexw64*) created in the folder.

1.3 Data Directory

While using this software, three new folders (data, result and UserSettings) will be created containing all necessary parameters, intermediate and ultimate results.
(1) data. This folder contains all intermediate and ultimate results in *.mat*

 © 2016 Kanchanawong Laboratory, Mechanobiology Institute, National University of Singapore

format.
(2) result. All ultimate results including plots (in *.fig* format for easy modification) and exported data sheets (in *.xlsx* format) are saved in this folder.
(3) UserSettings. This folder contains all user settings in GUIs.

2. Data Processing Steps

2.1 Load Image

To start, run the script, LoadImg.m to load the first GUI (Fig. 2) and click button ① to load the image, MT.tiff and open the second GUI (Fig. 3).

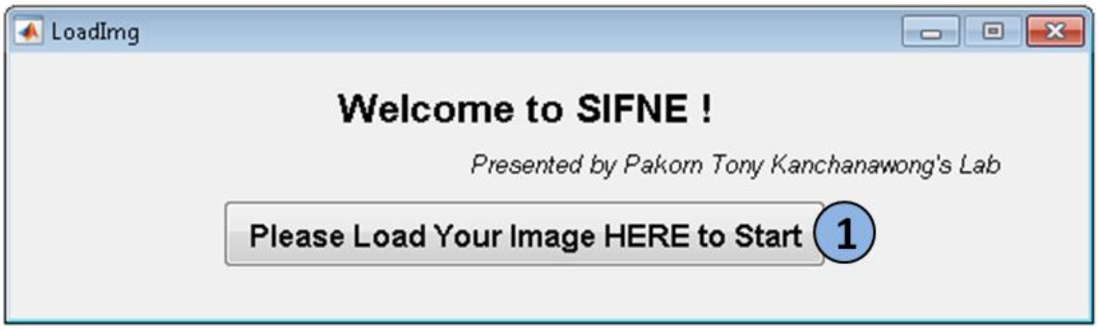

Figure 2. GUI for image loading.

2.2 Image Enhancement

2.2.1 Preview and Choose Region of Interest

The image enhancement method we use in our algorithm is line and orientation filter transform (LFT and OFT). For this enhancement approach, the user should define the radius and number of rotations of the scanning line segment at ① and ② (Fig. 3). Click button ③ to see the dimension of the scanning line segment (Fig. 4). Since MT.tiff is quite larger, the demonstrative region of scanning is very small as a red dot (Fig. 4, left). Hence, you can zoom in to see details (Fig. 4, right).
** The default values here have been optimized for MT.tiff.*

© 2016 Kanchanawong Laboratory, Mechanobiology Institute, National University of Singapore

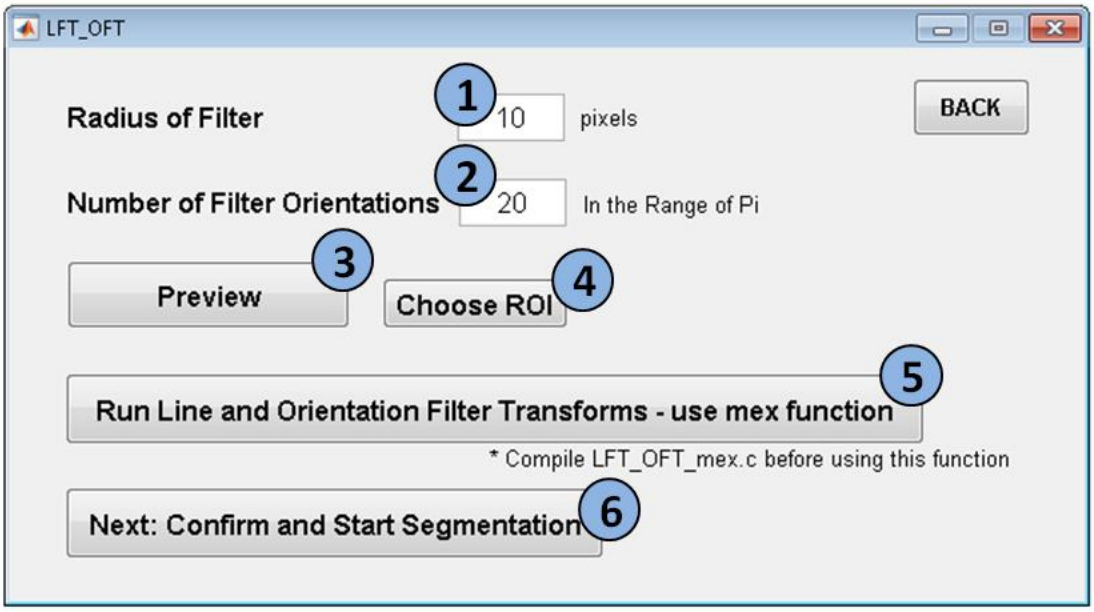

Figure 3. GUI for imaging enhancement.

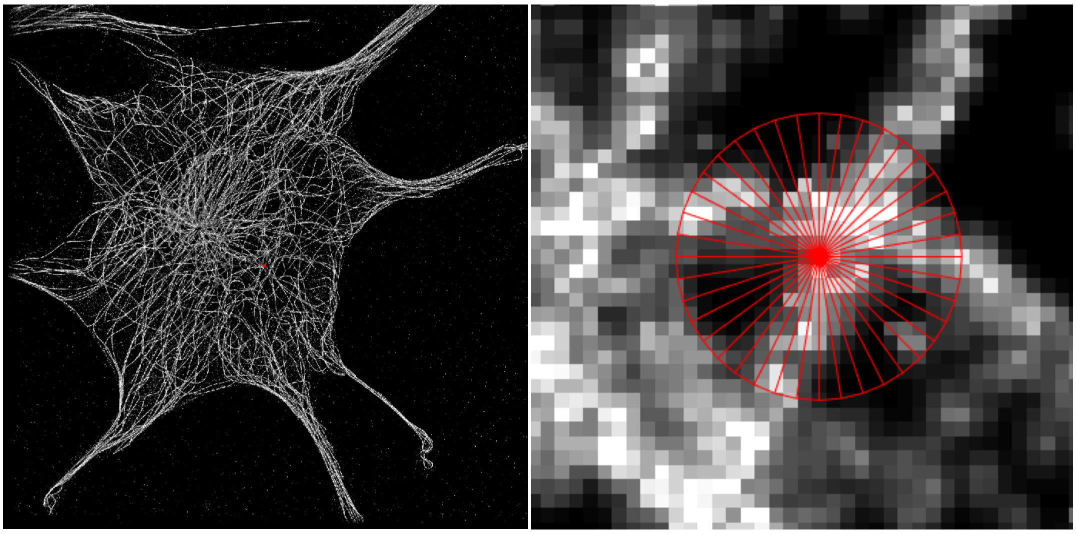

Figure 4. Preview of the filter dimension of LFT and OFT.

Click button ④ to choose region of interest.

** This region will be used to define the cell boundary and calculate the distance map in the analysis section. So the user should choose the ROI carefully.*

To do this, left-single-click all neighboring control points as highlighted in red rings (Fig. 5, left). Right-single-click at the last control point, the region will close itself (Fig. 5, right). Left click the center of the ROI twice.

The image will disappear and a message box will pop up telling you 'ROI Selected'. Click OK to continue.

** For large dataset, this may take around 20 seconds.*

 © 2016 Kanchanawong Laboratory, Mechanobiology Institute, National University of Singapore

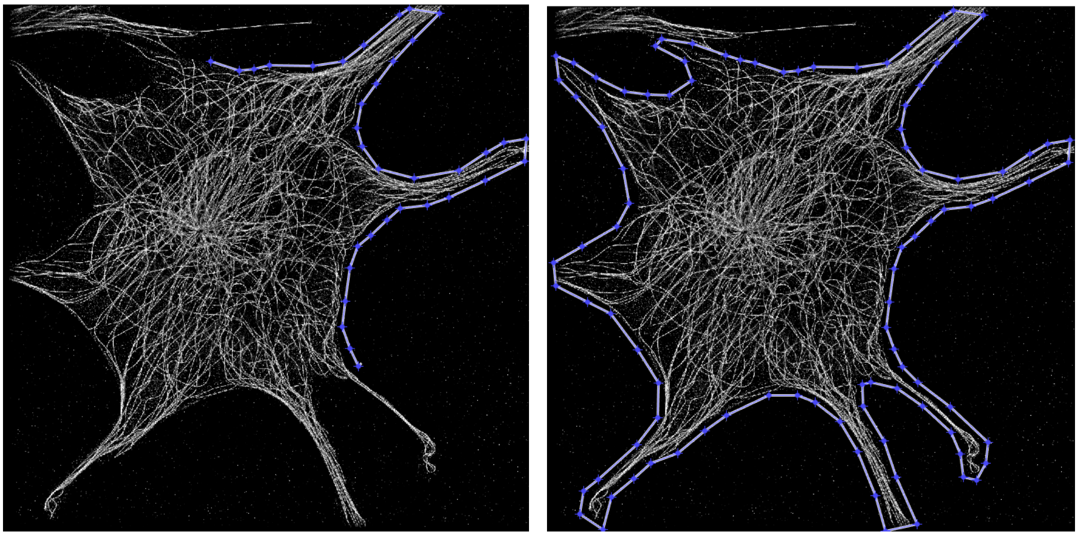

Figure 5. Selection of the region of interest.

2.2.2 Line and Orientation Filter Transform

Click the button ⑤ to run LFT and OFT. For MT.tiff, this step will take a couple of minutes. At the end of transformation, a message box will pop up telling you 'Transformation Done!' and the enhanced image will appear. When you are done, click button ⑥ to open the next GUI.

2.3 Segmentation and Tip Registration

2.3.1 Segmentation

Click button ① in the new GUI to automatically calculate the threshold for binarizing the enhanced image whose intensities has been normalized to 1 (Fig. 6) and the threshold will appear at ②. The default threshold is 1.42 times the value calculated using Otsu's method. Then a box will jump out telling you the Otsu's threshold. The binary image and overlay of original image and its extracted skeleton will appear for the user to evaluate by observation (Fig. 7). The user can feel free to manually define the threshold value between 0 and 1 at ② and click button ③ to assess again.

** The scale factor 1.42 is based on the noise level of MT.tiff and sensitivity test using synthetic images as described in the main text.*

** The user can feel free to zoom in to see details of figures.*

** The user does not have to stick to Otsu's threshold since it only provides you a reference value to start with.*

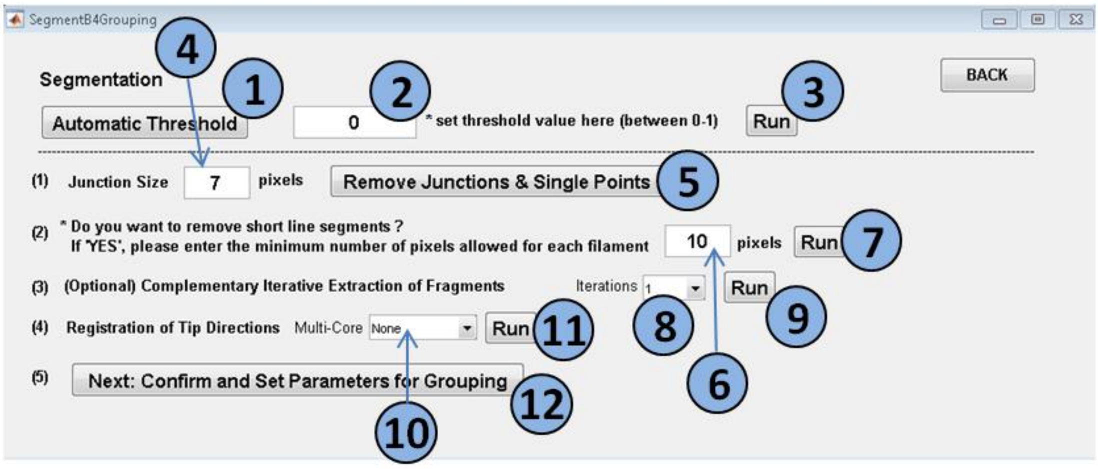

Figure 6. GUI for segmentation and tip registration.

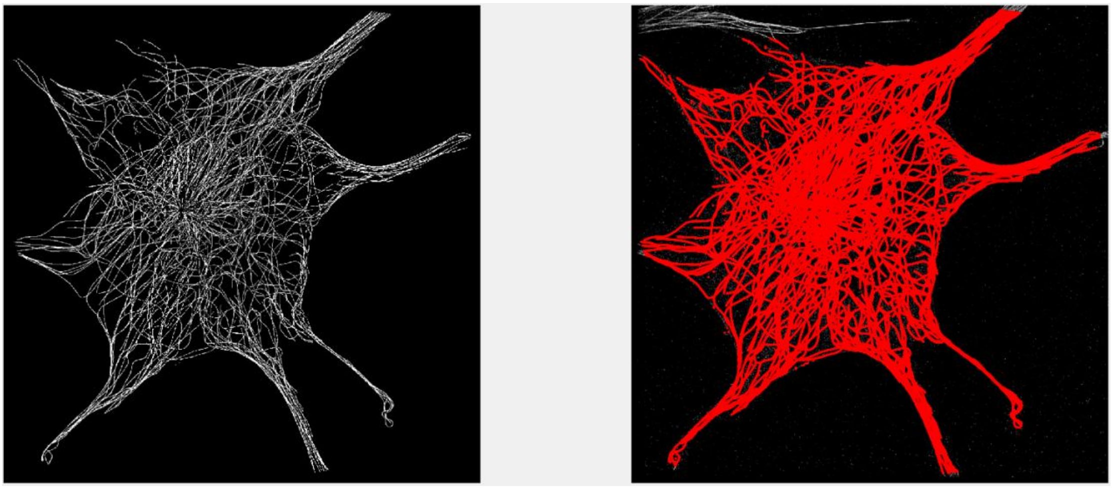

Figure 7. Skeleton of binarized image.

2.3.2 Junction Removal

To create the pool of minimal linear filament fragments, regions of junctions should be removed. Click button ⑤ to remove a local region of 7-by-7 pixels around each junction. This step will also remove single points. It is suggested to remove some short filament fragments primarily generated from noise by clicking button ⑦.

** The user can feel free to define the size of the junction region at ④ and minimal number of pixels in each filament fragment at ⑥.*

2.3.3 Iterative Extraction of Linear Fragments

Although this step is optional our result has shown that an iterative extraction of filament fragments will significantly recover undetected linear structures, especially in highly complex filament networks. Choose the number of additional iterations (from 1 to 5) you want to perform at ⑧ and click button ⑨. If you choose to iteratively extract filament fragments, the command window will display its progress including the iteration you are doing and number of

fragments added (Fig. 8).
** Noted that each iteration takes a couple of minutes for MT.tiff.*
** For spider.tiff, you can skip this step.*

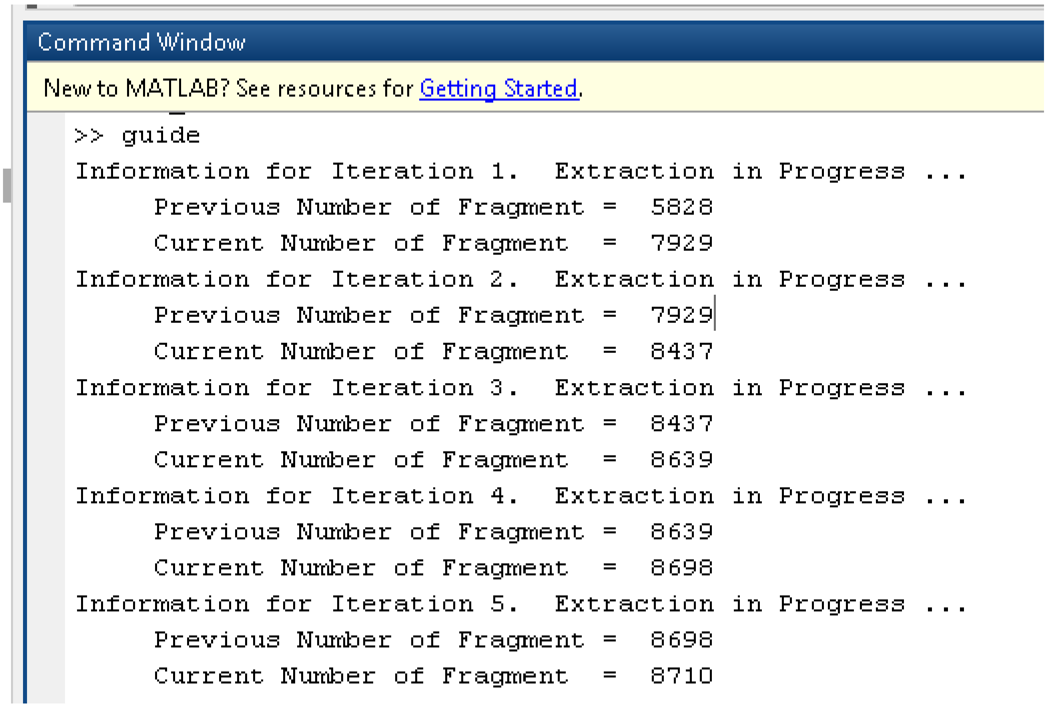

```
Command Window
New to MATLAB? See resources for Getting Started.
>> guide
Information for Iteration 1.  Extraction in Progress ...
     Previous Number of Fragment =  5828
     Current Number of Fragment  =  7929
Information for Iteration 2.  Extraction in Progress ...
     Previous Number of Fragment =  7929
     Current Number of Fragment  =  8437
Information for Iteration 3.  Extraction in Progress ...
     Previous Number of Fragment =  8437
     Current Number of Fragment  =  8639
Information for Iteration 4.  Extraction in Progress ...
     Previous Number of Fragment =  8639
     Current Number of Fragment  =  8698
Information for Iteration 5.  Extraction in Progress ...
     Previous Number of Fragment =  8698
     Current Number of Fragment  =  8710
```

Figure 8. Progress in iterative extraction of filament fragments.

2.3.4 Tip Registration

To register the propagation direction of each tip, the user can choose the computation mode at ⑩ as follows and click button ⑪,
(1) None: No parallel computing is needed.
(2) Half: Use half of the cores.
(3) Max: Use all cores.
Click button ⑫ and go to the last GUI.
** To increase the computational speed, we configured the program for parallel computing.*

2.4 Grouping and Analysis

2.4.1 Image Information

Indicate pixel size at ①.
Indicate the maximum curvature at ②.
** Noted that this parameter is only used to help automatically set other parameters. If the user doesn't know the max curvature of your filament, you can just ignore button ④ and manually set other parameters. For MT.tiff, the value is 1.*
Click button ④ to automatically set the conditions for fragment grouping.
This part is configured for parallel computing at. The user can choose at ③ accordingly.

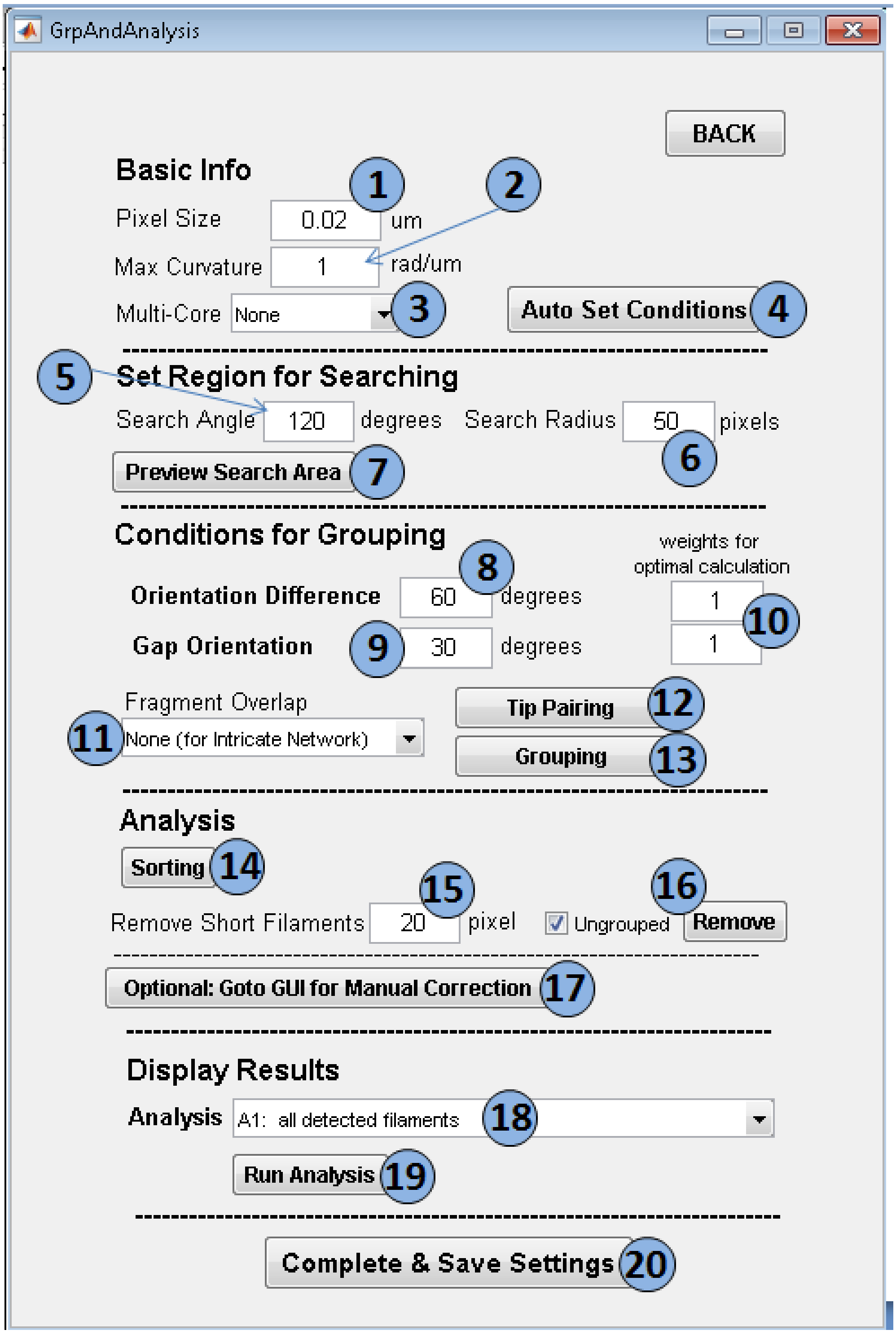

Figure 9. GUI for filament reconstruction and analysis.

2.4.2 Preview and Search Criteria

Indicate the search angle and radius at ⑤ and ⑥.

** Noted that these two parameters can be automatically set.*

Click button ⑦ to preview the search region and check whether it is suitable to cover most gaps that should be filled. An image of filament fragments will

 © 2016 Kanchanawong Laboratory, Mechanobiology Institute, National University of Singapore

appear request the user to click one location of network for preview (Fig. 10). Due to large data set, the search region (green color) looks quite small (Fig. 11, left). The user can zoom in to see it clearly (Fig. 11, right).

Indicate the maximum allowable orientation difference between two endpoints at ⑧ and the maximum allowable angle difference between base endpoint and gap vector at ⑨.

** Noted that the above two parameters can be automatically set.*

Indicate the weights for similarity and continuity conditions during scoring calculation at ⑩. The default value is 1.

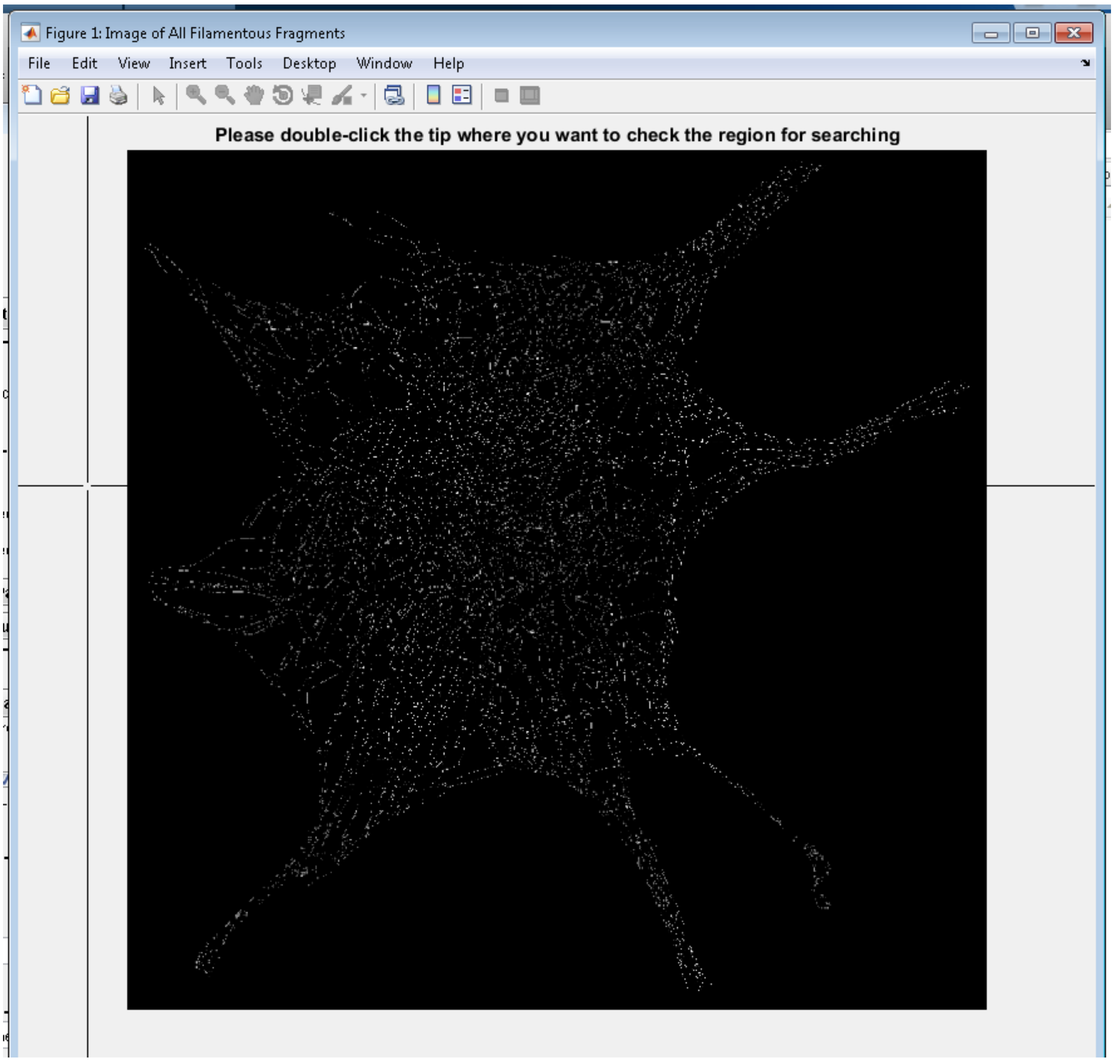

Figure 10. Interaction visualization of search region.

© 2016 Kanchanawong Laboratory, Mechanobiology Institute, National University of Singapore

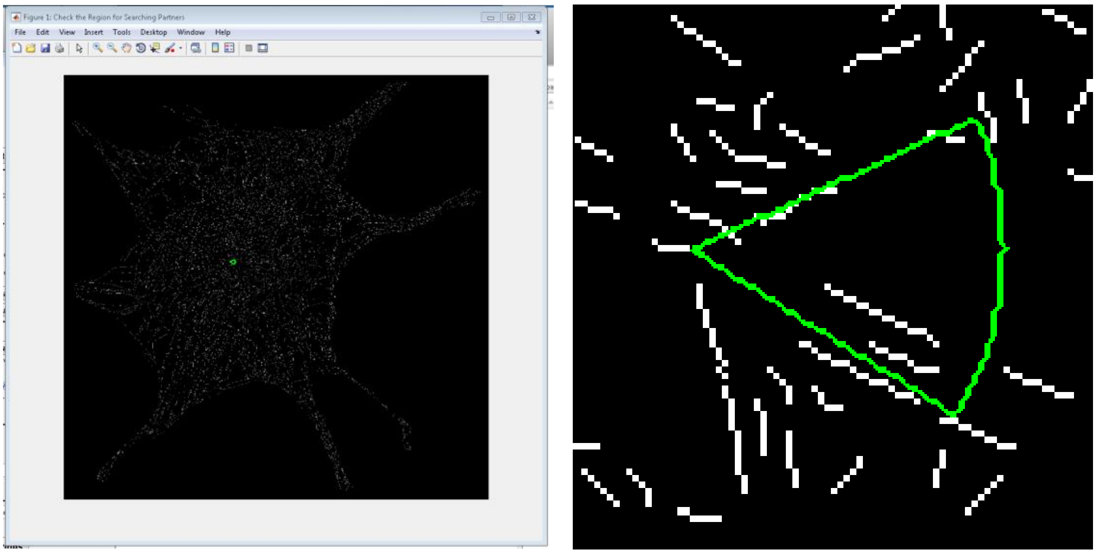

Figure 11. Zoom-in view of search region.

2.4.3 Tip Pairing and Grouping

In our algorithm we also allow the case that a fragment is combined into more than one composite filament in dependent on the maximum number of pairings it can form with other endpoints.

Two options at ⑪

(1) None (for Intricate Network)

(2) Allowed

** Noted that for MT.tiff, it is suggested to use the first option, 'None' due to high complexity of the network.*

Click button ⑫ to pair endpoints (also known as tips).

** Noted that you can use parallel computing in this step and indicate at ③. A message box will pop up after finish.*

Click button ⑬ to generate composite filaments.

** A message box will pop up after finish.*

2.4.4 Filament Sorting

Click button ⑭ to sort composite filaments

** Noted that you can use parallel computing in this step and indicate at ③. A message box will pop up after finish.*

You can define the minimum filament length allowed at ⑮ and toggle at ⑯ to choose whether you want to remove ungrouped filament fragments.

** Tick: To remove ungrouped fragments.*

Optional: Click button ⑰ to open another GUI for manual correction. This will be described in section 3.

2.4.5 Analysis Features

SIFNE provides the following analysis and the user can select at 18 and then click button 19.

A1: all detected filaments

A2: junctions

 © 2016 Kanchanawong Laboratory, Mechanobiology Institute, National University of Singapore

A3: histogram of orientations
A4: curvature
A5: export into excel

2.4.5.1 Detected Filament

Image of the skeleton of binarized image overlaid with composite filaments shown in different colors (Fig. 12). Cell boundary is indicated in white.

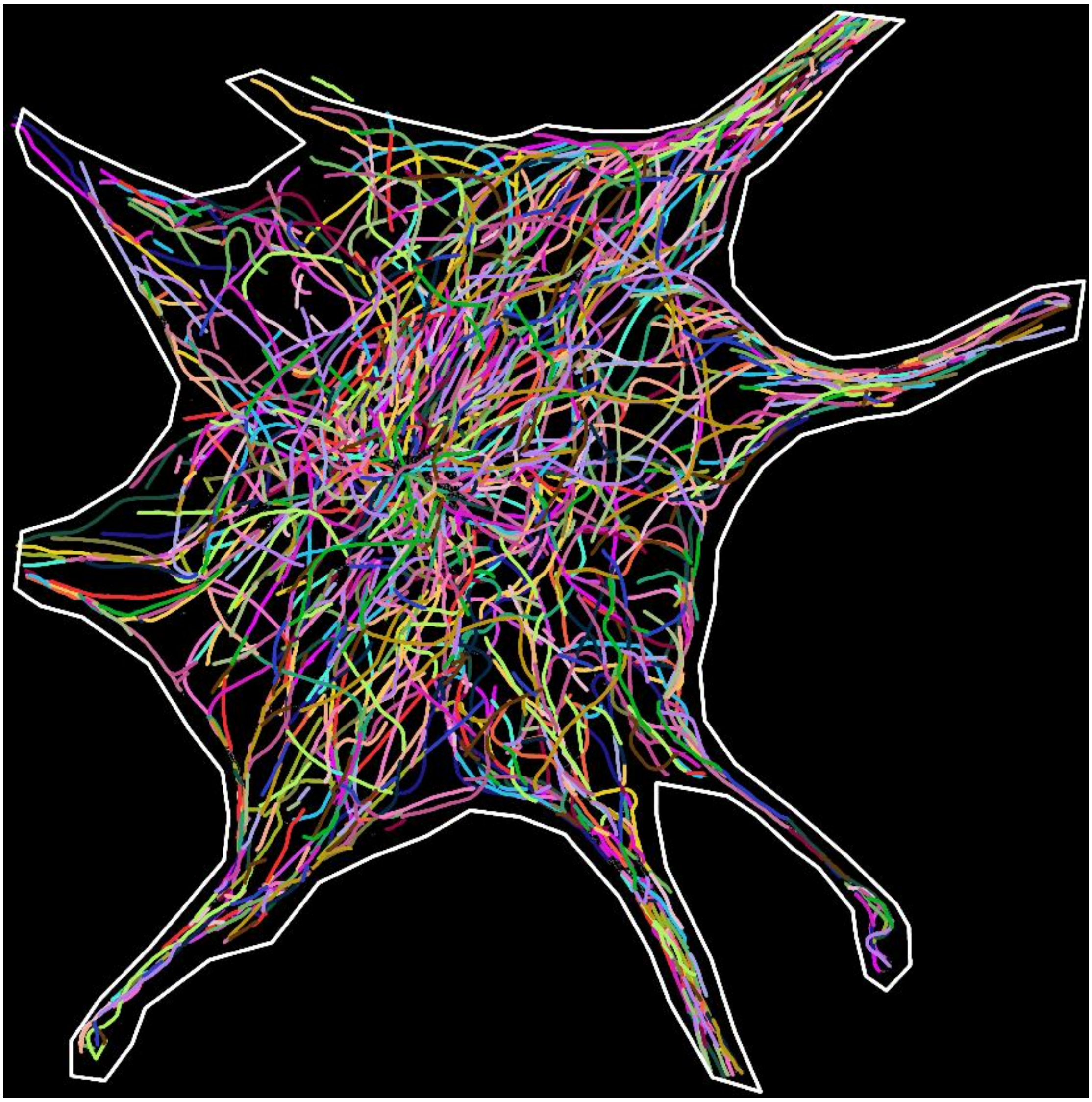

Figure 12. Extracted filaments.

2.4.5.2 Junctions

Enlarged image of all composite filaments (black) overlaid with all centroids of junctions (green) (Fig. 13, left). The background image is distance map as a function of the distance to cell edge.

** Unit of color bar: μm.*

Distribution of junctions as a function of their distances to cell boundary (Fig. 13, right).

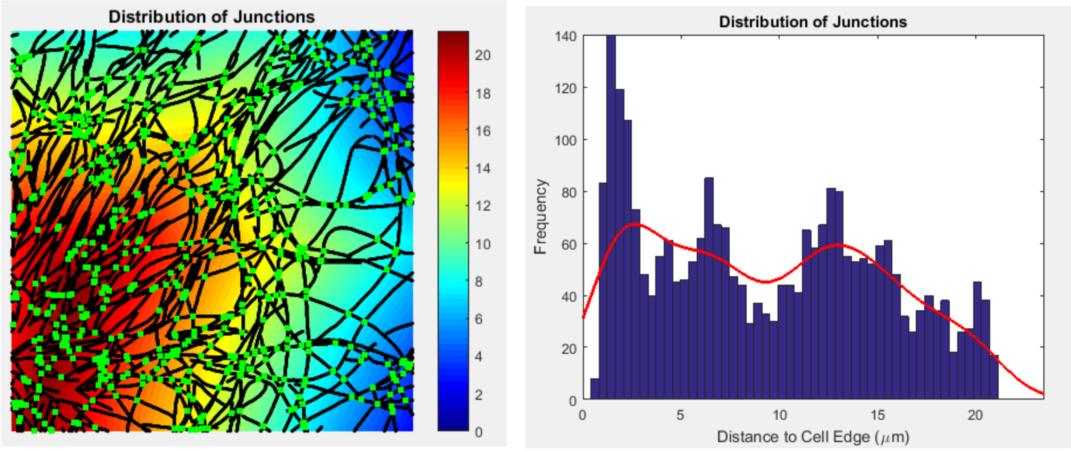

Figure 13. Junction analysis.

2.4.5.3 Filament Orientation

Rose plot of all filament orientations.

** The orientations of filaments ranges from -90° to 90° (Fig. 14, left).*

Spatial distribution of all orientations as a function of the distance between filament centroids and cell edge (Fig. 14, right).

** Unit of colorbar: counts/frequency.*

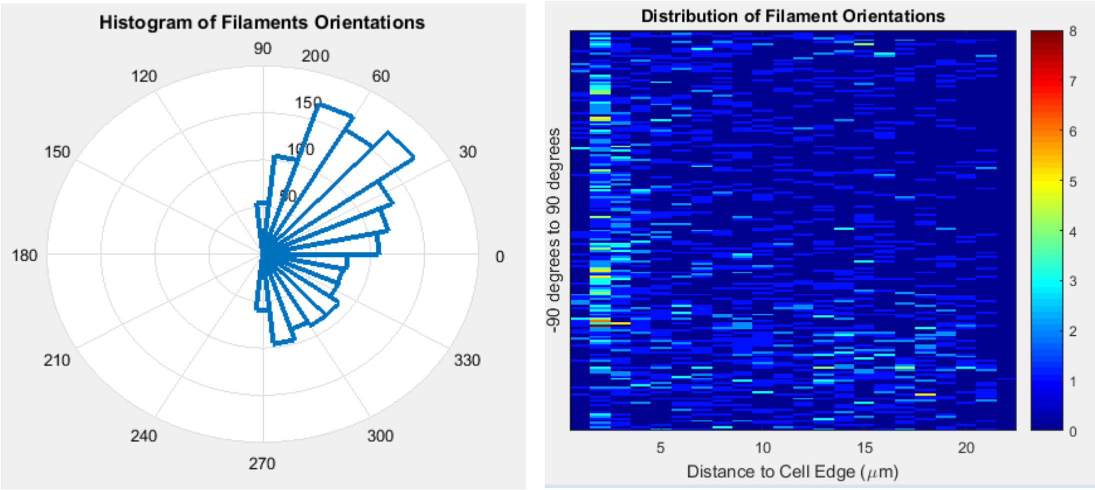

Figure 14. Orientation analysis.

2.4.5.4 Curvature

Enlarged image of filament curvatures (Fig. 15, left).

** Unit of colorbar: μm^{-1}*

Histogram of curvatures all composite filament pixels (Fig. 15, middle).

Plot of the mean curvatures of all composite filaments as a function of the distances between the centroids of filaments and cell edge (Fig. 15, right).

© 2016 Kanchanawong Laboratory, Mechanobiology Institute, National University of Singapore

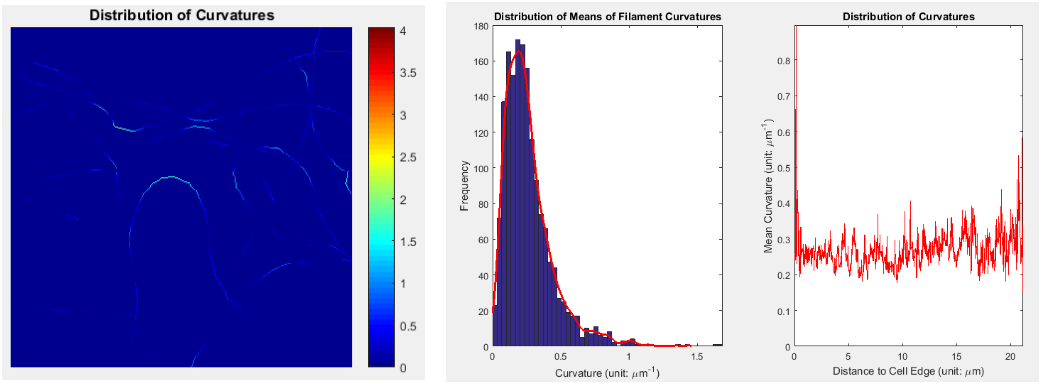

Figure 15. Curvature analysis.

2.4.5.5 Export into Excel

Export the information of composite filaments, junctions and fragment linkage into an excel file for more customized analysis. The exported excel file includes 4 worksheets as follows.

Worksheet 1: Information of all composite filaments (Fig. 16, 17)

Worksheet 2: Information of all filament fragments (Fig. 18)

Worksheet 3: Linkage information before removing short filaments and ungrouped fragments (Fig. 19)

Worksheet 4: Linkage information after removing short filaments and ungrouped fragments(Fig. 20)

When the user is done with all analysis, click button ⑳ to complete and save settings.

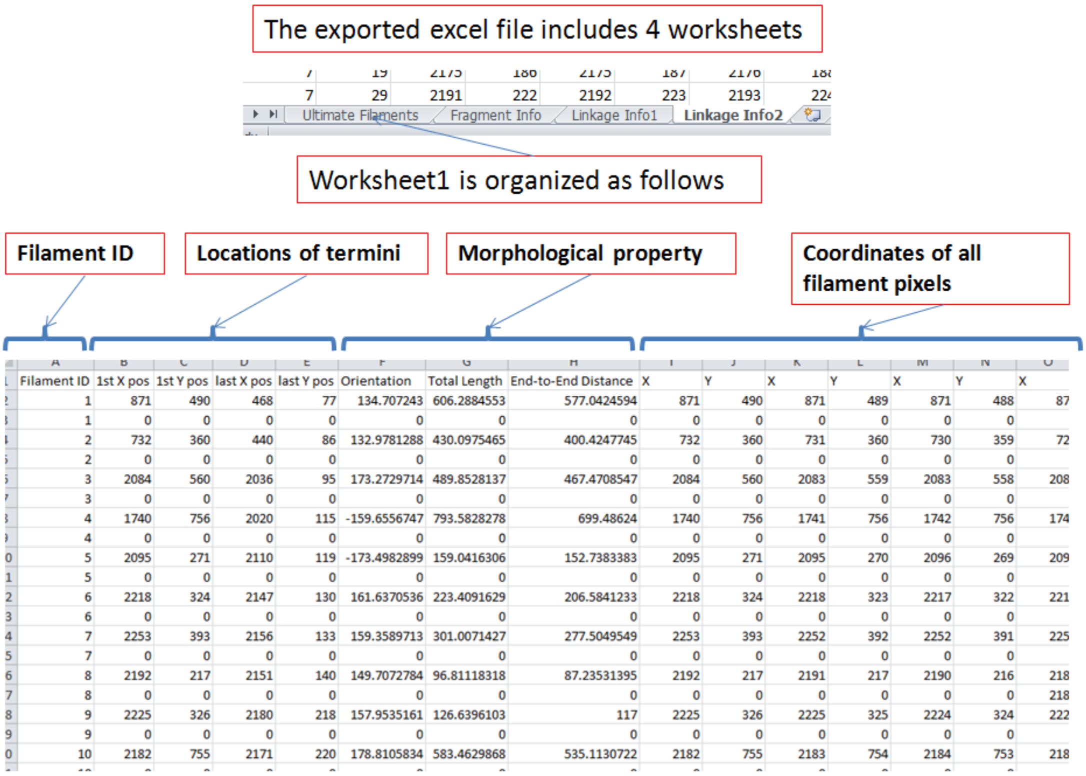

Filament ID	1st X pos	1st Y pos	last X pos	last Y pos	Orientation	Total Length	End-to-End Distance	X	Y	X	Y	X	Y	X
1	871	490	468	77	134.707243	606.2884553	577.0424594	871	490	871	489	871	488	87
1	0	0	0	0	0	0	0	0	0	0	0	0	0	
2	732	360	440	86	132.9781288	430.0975465	400.4247745	732	360	731	360	730	359	72
2	0	0	0	0	0	0	0	0	0	0	0	0	0	
3	2084	560	2036	95	173.2729714	489.8528137	467.4708547	2084	560	2083	559	2083	558	208
3	0	0	0	0	0	0	0	0	0	0	0	0	0	
4	1740	756	2020	115	-159.6556747	793.5828278	699.48624	1740	756	1741	756	1742	756	174
4	0	0	0	0	0	0	0	0	0	0	0	0	0	
5	2095	271	2110	119	-173.4982899	159.0416306	152.7383383	2095	271	2095	270	2096	269	209
5	0	0	0	0	0	0	0	0	0	0	0	0	0	
6	2218	324	2147	130	161.6370536	223.4091629	206.5841233	2218	324	2218	323	2217	322	221
6	0	0	0	0	0	0	0	0	0	0	0	0	0	
7	2253	393	2156	133	159.3589713	301.0071427	277.5049549	2253	393	2252	392	2252	391	225
7	0	0	0	0	0	0	0	0	0	0	0	0	0	
8	2192	217	2151	140	149.7072784	96.81118318	87.23531395	2192	217	2191	217	2190	216	218
8	0	0	0	0	0	0	0	0	0	0	0	0	0	218
9	2225	326	2180	218	157.9535161	126.6396103	117	2225	326	2225	325	2224	324	222
9	0	0	0	0	0	0	0	0	0	0	0	0	0	
10	2182	755	2171	220	178.8105834	583.4629868	535.1130722	2182	755	2183	754	2184	753	218

Figure 16. Exported Excel File for 'all composite filaments' option.

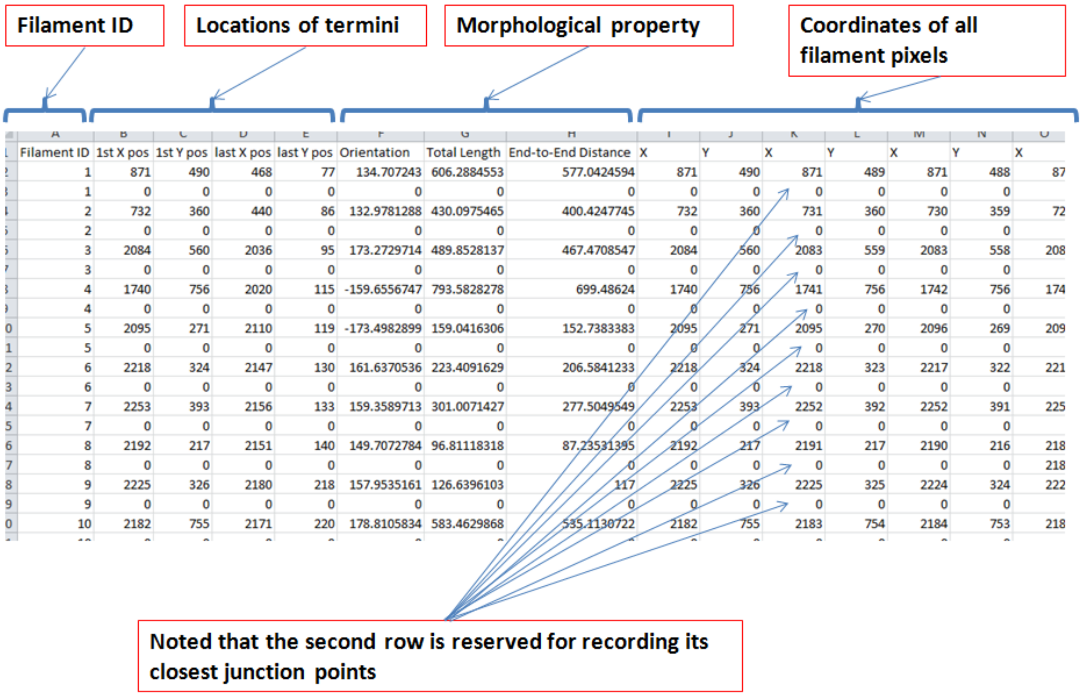

Filament ID	1st X pos	1st Y pos	last X pos	last Y pos	Orientation	Total Length	End-to-End Distance	X	Y	X	Y	X	Y	X
1	871	490	468	77	134.707243	606.2884553	577.0424594	871	490	871	489	871	488	87
1	0	0	0	0	0	0	0	0	0	0	0	0	0	
2	732	360	440	86	132.9781288	430.0975465	400.4247745	732	360	731	360	730	359	72
2	0	0	0	0	0	0	0	0	0	0	0	0	0	
3	2084	560	2036	95	173.2729714	489.8528137	467.4708547	2084	560	2083	559	2083	558	208
3	0	0	0	0	0	0	0	0	0	0	0	0	0	
4	1740	756	2020	115	-159.6556747	793.5828278	699.48624	1740	756	1741	756	1742	756	174
4	0	0	0	0	0	0	0	0	0	0	0	0	0	
5	2095	271	2110	119	-173.4982899	159.0416306	152.7383383	2095	271	2095	270	2096	269	209
5	0	0	0	0	0	0	0	0	0	0	0	0	0	
6	2218	324	2147	130	161.6370536	223.4091629	206.5841233	2218	324	2218	323	2217	322	221
6	0	0	0	0	0	0	0	0	0	0	0	0	0	
7	2253	393	2156	133	159.3589713	301.0071427	277.5049549	2253	393	2252	392	2252	391	225
7	0	0	0	0	0	0	0	0	0	0	0	0	0	
8	2192	217	2151	140	149.7072784	96.81118318	87.23531395	2192	217	2191	217	2190	216	218
8	0	0	0	0	0	0	0	0	0	0	0	0	0	218
9	2225	326	2180	218	157.9535161	126.6396103	117	2225	326	2225	325	2224	324	222
9	0	0	0	0	0	0	0	0	0	0	0	0	0	
10	2182	755	2171	220	178.8105834	583.4629868	535.1130722	2182	755	2183	754	2184	753	218

Figure 17. Junction information in exported Excel File

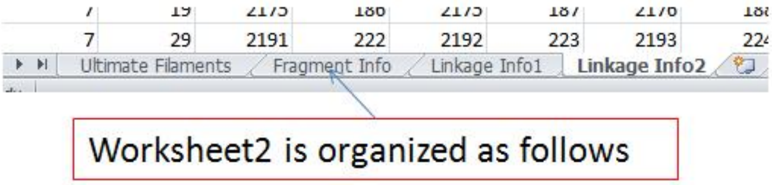

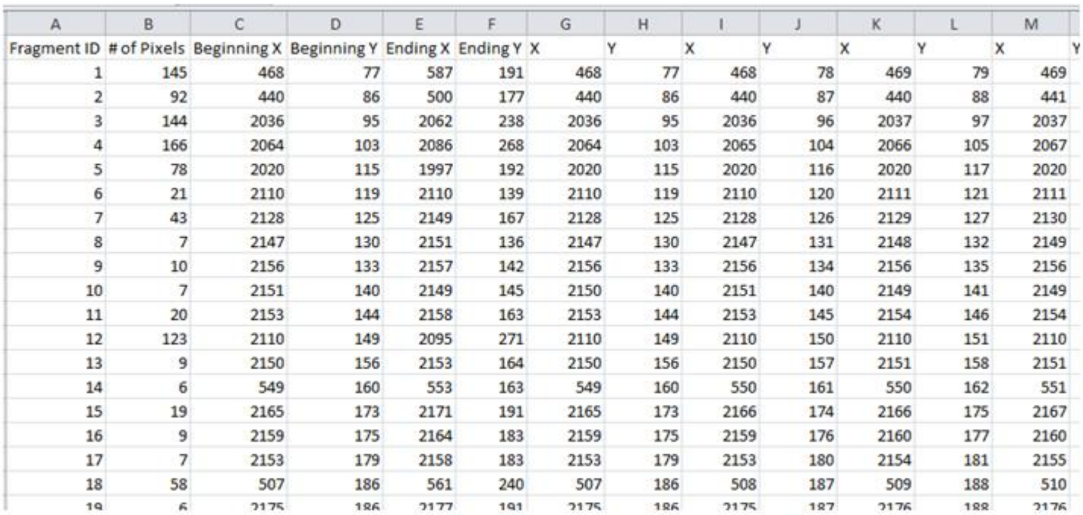

Fragment ID	# of Pixels	Beginning X	Beginning Y	Ending X	Ending Y	X	Y	X	Y	X	Y	X	Y
1	145	468	77	587	191	468	77	468	78	469	79	469	
2	92	440	86	500	177	440	86	440	87	440	88	441	
3	144	2036	95	2062	238	2036	95	2036	96	2037	97	2037	
4	166	2064	103	2086	268	2064	103	2065	104	2066	105	2067	
5	78	2020	115	1997	192	2020	115	2020	116	2020	117	2020	
6	21	2110	119	2110	139	2110	119	2110	120	2111	121	2111	
7	43	2128	125	2149	167	2128	125	2128	126	2129	127	2130	
8	7	2147	130	2151	136	2147	130	2147	131	2148	132	2149	
9	10	2156	133	2157	142	2156	133	2156	134	2156	135	2156	
10	7	2151	140	2149	145	2150	140	2151	140	2149	141	2149	
11	20	2153	144	2158	163	2153	144	2153	145	2154	146	2154	
12	123	2110	149	2095	271	2110	149	2110	150	2110	151	2110	
13	9	2150	156	2153	164	2150	156	2150	157	2151	158	2151	
14	6	549	160	553	163	549	160	550	161	550	162	551	
15	19	2165	173	2171	191	2165	173	2166	174	2166	175	2167	
16	9	2159	175	2164	183	2159	175	2159	176	2160	177	2160	
17	7	2153	179	2158	183	2153	179	2153	180	2154	181	2155	
18	58	507	186	561	240	507	186	508	187	509	188	510	

Figure 18. Export into excel. All filament fragment information.

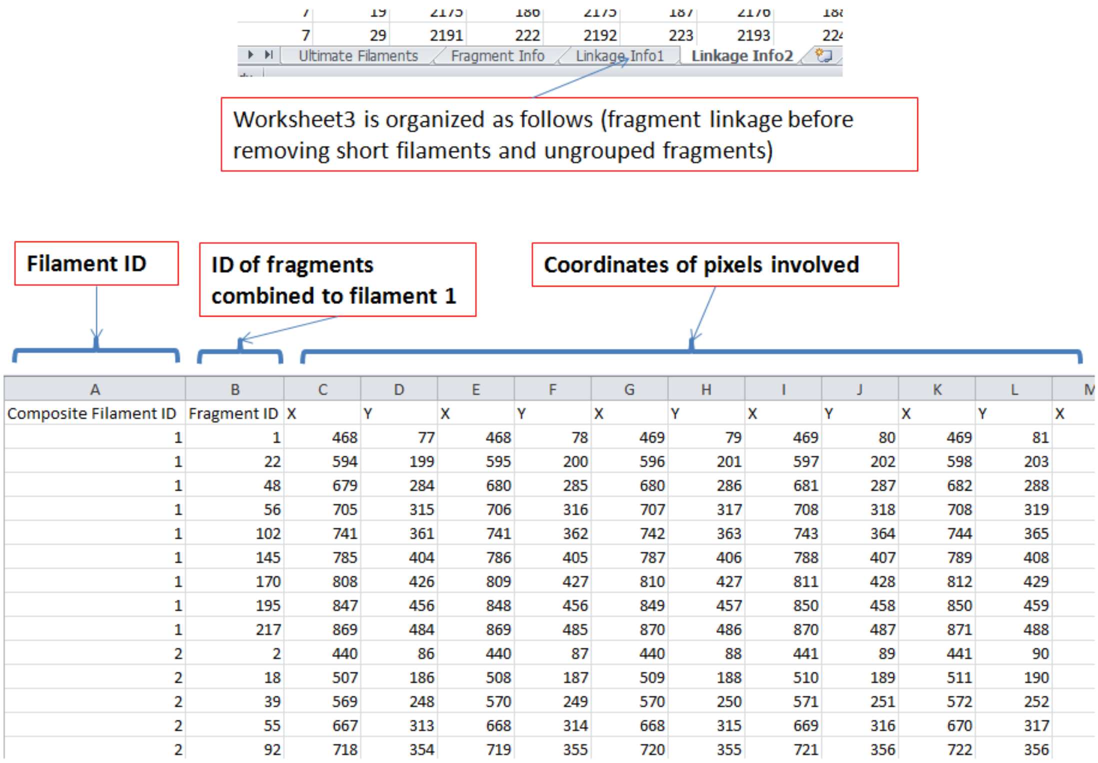

Composite Filament ID	Fragment ID	X	Y	X	Y	X	Y	X	Y	X	Y	X
1	1	468	77	468	78	469	79	469	80	469	81	
1	22	594	199	595	200	596	201	597	202	598	203	
1	48	679	284	680	285	680	286	681	287	682	288	
1	56	705	315	706	316	707	317	708	318	708	319	
1	102	741	361	741	362	742	363	743	364	744	365	
1	145	785	404	786	405	787	406	788	407	789	408	
1	170	808	426	809	427	810	427	811	428	812	429	
1	195	847	456	848	456	849	457	850	458	850	459	
1	217	869	484	869	485	870	486	870	487	871	488	
2	2	440	86	440	87	440	88	441	89	441	90	
2	18	507	186	508	187	509	188	510	189	511	190	
2	39	569	248	570	249	570	250	571	251	572	252	
2	55	667	313	668	314	668	315	669	316	670	317	
2	92	718	354	719	355	720	355	721	356	722	356	

Figure 19. Linkage information before the removal of short filaments and ungrouped fragments in exported Excel file

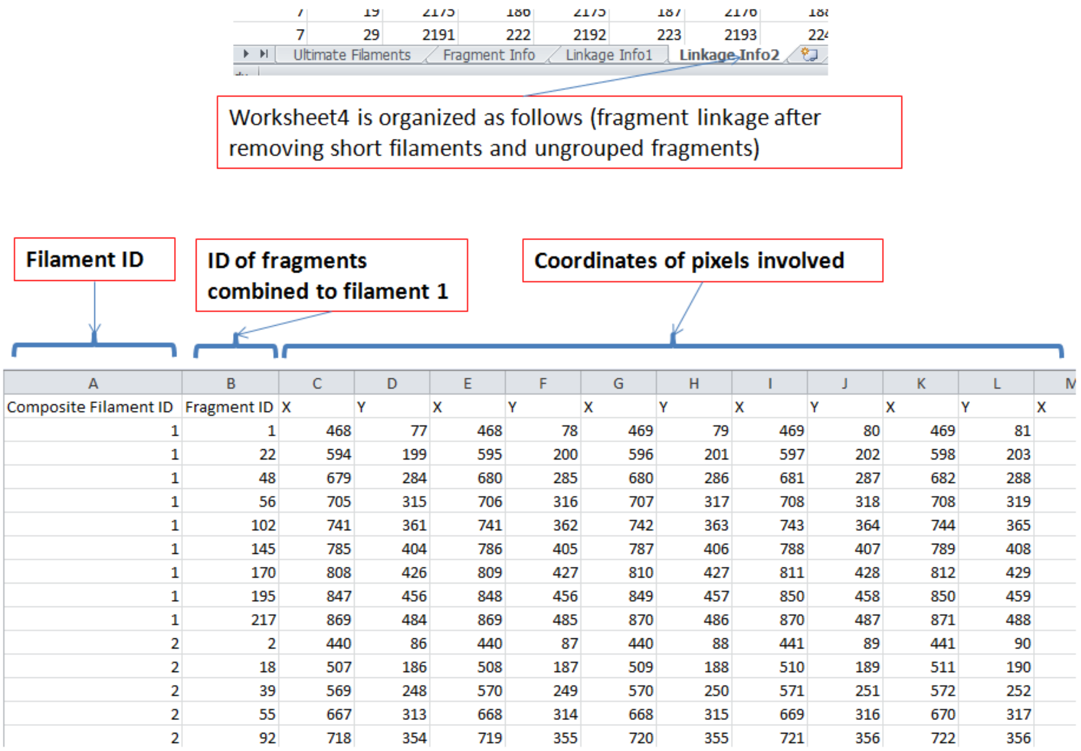

Composite Filament ID	Fragment ID	X	Y	X	Y	X	Y	X	Y	X	Y	X
1	1	468	77	468	78	469	79	469	80	469	81	
1	22	594	199	595	200	596	201	597	202	598	203	
1	48	679	284	680	285	680	286	681	287	682	288	
1	56	705	315	706	316	707	317	708	318	708	319	
1	102	741	361	741	362	742	363	743	364	744	365	
1	145	785	404	786	405	787	406	788	407	789	408	
1	170	808	426	809	427	810	427	811	428	812	429	
1	195	847	456	848	456	849	457	850	458	850	459	
1	217	869	484	869	485	870	486	870	487	871	488	
2	2	440	86	440	87	440	88	441	89	441	90	
2	18	507	186	508	187	509	188	510	189	511	190	
2	39	569	248	570	249	570	250	571	251	572	252	
2	55	667	313	668	314	668	315	669	316	670	317	
2	92	718	354	719	355	720	355	721	356	722	356	

Figure 20. Linkage information after the removal of short filaments and ungrouped fragments in exported Excel file.

© 2016 Kanchanawong Laboratory, Mechanobiology Institute, National University of Singapore

3. Manual Correction

3.1 Initialization

Click button ⑰ in Fig. 9 to open the GUI for manual correction as shown in Fig. 21.
Click button ① to initialize and the image for correction will appear (Fig. 22). Detected filament pixels are shown in cyan and endpoints are marked in red.
During correction, click button ⑨ if the user wants to save corrected filaments and continue after some time (e.g. to continue tomorrow). Click button ② if you want to continue from previously saved results.
To highlight a filament (help the user clearly distinguish one from others), click button ③ and then click any point of the filament the user wants to highlighted in the image (the selected filament will be highlighted in red) (Fig. 23).
To refresh the current figure, click button ④.

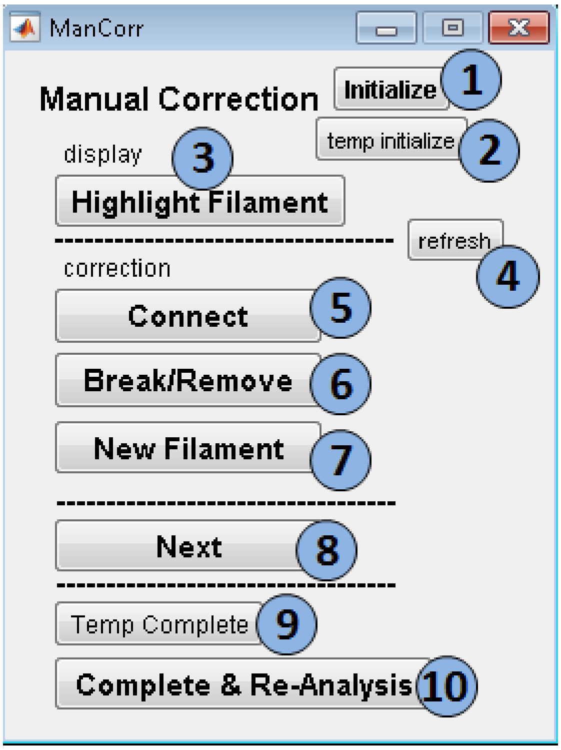

Figure 21. GUI for manual correction.

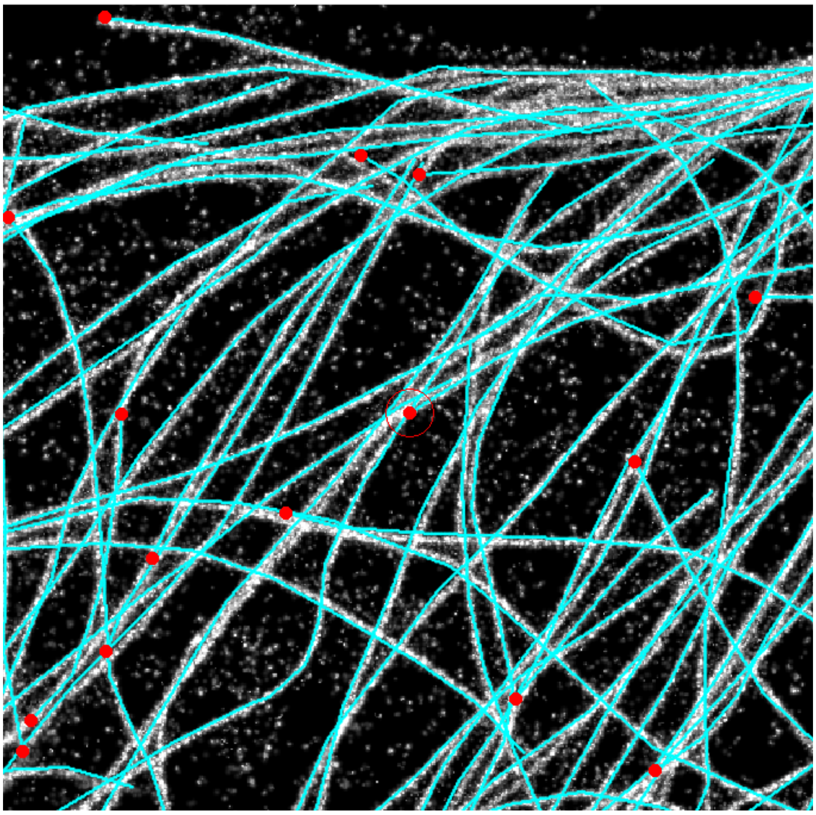

Figure 22. Example of an image to be corrected.

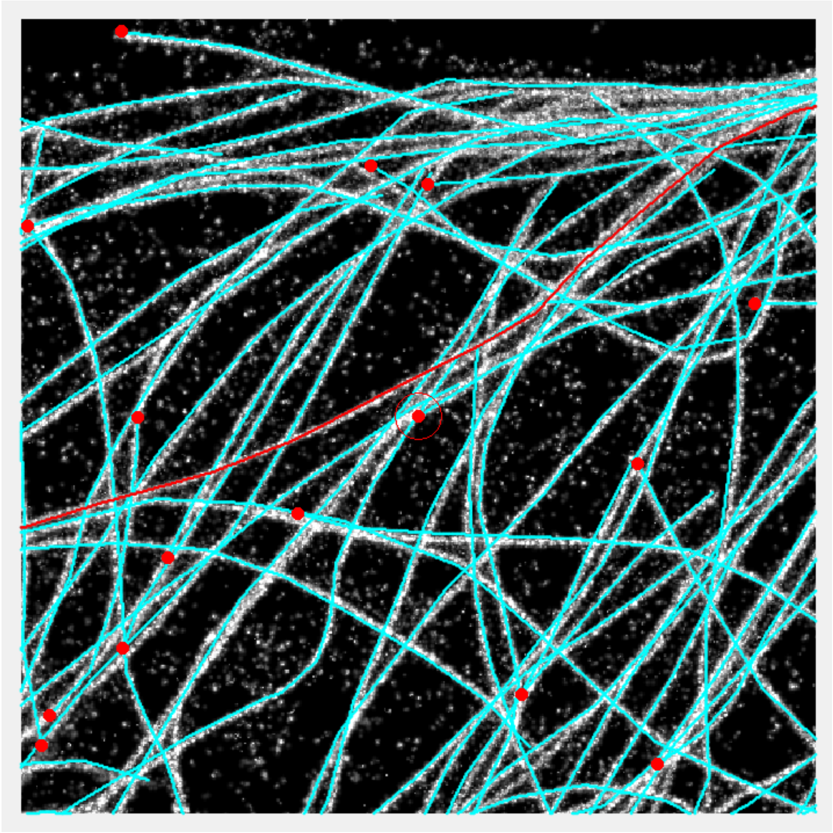

Figure 23. Highlighed filament.

3.2 Connect

To connect two tips, click button ⑤ and then click the two red endpoints in the image.

3.3 Break/Remove

To remove an entire or partial filament, click button ⑥ and then click the filament to modify in the image (its color will become red).
Subsequently, use mouse to enclose the region you want to remove. This step should be performed as follows
(1) left-click a point and hold
(2) enclose the region to remove (not necessarily go back to the first point)
(3) release your finger
(4) new filament information will be updated in the image

3.4 New Filament

To create a new filament, click button ⑦ and then go back to the image to draw a new filament.
The drawing should be performed as follows
(1) left-click the first control point and release
(2) left-click the second control point and release
(3) repeat step2 till the last control point
(4) right-click to complete this drawing

3.5 Continue

During the manual correction, the user should check the situation at each tip. This progress can be monitored in the command window (Fig. 24). Click button ⑧ to check the situation around the next tip

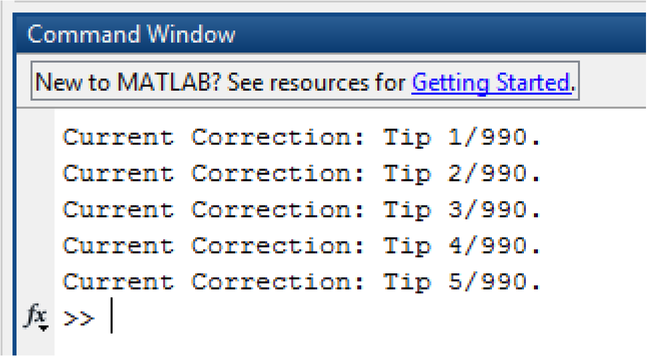

Figure 24. Progress in manual correction.

3.6 Save

When completed, click button ⑩ to re-analyze all corrected filaments.

© 2016 Kanchanawong Laboratory, Mechanobiology Institute, National University of Singapore

Contact Information and Updates

Contact 1: Dr Pakorn Kanchanawong (biekp@nus.edu.sg)
Contact 2: Zhen Zhang (a0045820@u.nus.edu)
Website: https://sites.google.com/site/kanchanawonglab/home
