# Supplementary material for: Smad4 controls signaling robustness and morphogenesis by differentially contributing to the Nodal and BMP pathways
Source: Nat Commun. 2021 Nov 4;12:6374. doi: 10.1038/s41467-021-26486-3 (PMC8569018; doi:10.1038/s41467-021-26486-3)
Supplement: Supplementary file 10 — Software code [file 41467_2021_26486_MOESM10_ESM.zip › ALYtools-master 08.21/HelperFunctions/GUI Layout Toolbox/layoutdoc/Getting Started.html]

Getting Started


|  |  |
| --- | --- |
| **GUI Layout Toolbox 2.1** |  |

# Getting Started

This chapter gives an overview of this package, including its purpose,
how to install it and any known issues at the time of release.


  


## Contents

- 1. What is GUI Layout Toolbox?
- 2. Compatibility considerations
- 3. Release notes
- 4. Installation
- 5. Support
- 6. Acknowledgements

  

|  |  |  |  |  |
| --- | --- | --- | --- | --- |
|  | Front page | [Top] | What is GUI Layout Toolbox? |  |

© 2014 The MathWorks Ltd
• Terms of Use
• Patents
• Trademarks
