# Supplementary material for: Smad4 controls signaling robustness and morphogenesis by differentially contributing to the Nodal and BMP pathways
Source: Nat Commun. 2021 Nov 4;12:6374. doi: 10.1038/s41467-021-26486-3 (PMC8569018; doi:10.1038/s41467-021-26486-3)
Supplement: Supplementary file 10 — Software code [file 41467_2021_26486_MOESM10_ESM.zip › ALYtools-master 08.21/HelperFunctions/GUI Layout Toolbox/layoutdoc/User guide7_4.html]

onListSelection


|  |  |
| --- | --- |
| **User guide** |  |

  

## 7.4: onListSelection

Finally, let's have a look at how one of the callbacks works: "onListSelection", the function
that is called when an item is selected in the listbox. The other
callbacks obey the same pattern.

The basic rule of thumb is that a callback should not update any part of
the user interface directly - it's job is to respond to user interaction by changing the "data" structure.
In this example, each callback changes the underlying data structure
then asks the interface to refresh. This might mean that things update in the
interface that don't need to, but ensures the callbacks remain simple and that all
interface update logic is in one place. Extending this to more granular interface updates
is straightforward. See Scalability for more details.

For the listbox callback, the "src" argument is a handle to the
listbox and we simply need to update the SelectedDemo field of "data"
to reflect the new selection. We then ask the rest of the interface to
update in response to the change.

```
                % User selected a demo from the list - update "data" and refresh
data.SelectedDemo = get( src, 'Value' );
updateInterface();
redrawDemo();
```

(Full source code for this application is available here:
[ view
| edit
| run ]
)

  

|  |  |  |  |  |
| --- | --- | --- | --- | --- |
|  | updateInterface | [Top] | Running it |  |

© 2014 The MathWorks Ltd
• Terms of Use
• Patents
• Trademarks
