# Supplementary material for: Smad4 controls signaling robustness and morphogenesis by differentially contributing to the Nodal and BMP pathways
Source: Nat Commun. 2021 Nov 4;12:6374. doi: 10.1038/s41467-021-26486-3 (PMC8569018; doi:10.1038/s41467-021-26486-3)
Supplement: Supplementary file 10 — Software code [file 41467_2021_26486_MOESM10_ESM.zip › ALYtools-master 08.21/HelperFunctions/GUI Layout Toolbox/layoutdoc/User guide4_1.html]

Context help


|  |  |
| --- | --- |
| **User guide** |  |

  

## 4.1: Context help

When a `uix.BoxPanel` has its `HelpFcn`
filled in, a help button () is shown in the upper-right of the
title-bar. When the user clicks this button the specified function
is called.

### For example:

Here we hookup the HelpFcn to simply bring up
the MATLAB documentation for each command. Clicking any of the "?" buttons
will cause the MATLAB help browser to open.

```
f = figure( 'Name', 'uix.BoxPanel Help Example' );
b = uix.HBox( 'Parent', f );
uix.BoxPanel( 'Parent', b, 'Title', 'sin', 'HelpFcn', @(a,b) doc('sin') );
uix.BoxPanel( 'Parent', b, 'Title', 'cos', 'HelpFcn', @(a,b) doc('cos') );
uix.BoxPanel( 'Parent', b, 'Title', 'tan', 'HelpFcn', @(a,b) doc('tan') );
```

  

|  |  |  |  |  |
| --- | --- | --- | --- | --- |
|  | Advanced maneuvers with panels | [Top] | Minimize and maximize |  |

© 2014 The MathWorks Ltd
• Terms of Use
• Patents
• Trademarks
