# Supplementary material for: Smad4 controls signaling robustness and morphogenesis by differentially contributing to the Nodal and BMP pathways
Source: Nat Commun. 2021 Nov 4;12:6374. doi: 10.1038/s41467-021-26486-3 (PMC8569018; doi:10.1038/s41467-021-26486-3)
Supplement: Supplementary file 10 — Software code [file 41467_2021_26486_MOESM10_ESM.zip › ALYtools-master 08.21/HelperFunctions/GUI Layout Toolbox/layoutdoc/User guide1_2.html]

Types of layout


|  |  |
| --- | --- |
| **User guide** |  |

  

## 1.2: Types of layout

The layouts in this toolbox come in three forms:

1. **Panels**: show a single child element with some decoration. Other children
   of the layout are hidden from view. The visible child can be switched. Available
   panels include
   Panel,
   CardPanel,
   BoxPanel and
   TabPanel.
2. **Boxes**: arrange children linearly in a single row or column. Available boxes include
   HBox,
   VBox,
   HBoxFlex and
   VBoxFlex.
3. **Grids**: (also known as tables) arrange children in a two-dimensional grid. Available grids include
   Grid and
   GridFlex.

  

|  |  |  |  |  |
| --- | --- | --- | --- | --- |
|  | Layout basics | [Top] | Sizes and units |  |

© 2014 The MathWorks Ltd
• Terms of Use
• Patents
• Trademarks
