# Supplementary material for: Smad4 controls signaling robustness and morphogenesis by differentially contributing to the Nodal and BMP pathways
Source: Nat Commun. 2021 Nov 4;12:6374. doi: 10.1038/s41467-021-26486-3 (PMC8569018; doi:10.1038/s41467-021-26486-3)
Supplement: Supplementary file 10 — Software code [file 41467_2021_26486_MOESM10_ESM.zip › ALYtools-master 08.21/HelperFunctions/GUI Layout Toolbox/layoutdoc/index.html]

GUI Layout Toolbox documentation


# GUI Layout Toolbox documentation

**Version: 2.1**

This toolbox provides tools to create sophisticated MATLAB graphical
user interfaces that resize gracefully. The classes supplied can be
used in combination to produce virtually any user interface layout.

- Arrange MATLAB user-interface components horizontally, vertically or in grids
- Ability to mix fixed size and variable size elements
- Dynamic resizing of elements by dragging dividers
- Use panels and tabs for switching interface pages

This toolbox was developed by
David Sampson and
Ben Tordoff from the
Consulting Services group
at MathWorks.

## Contents:

|  |  |  |
| --- | --- | --- |
|  | **Getting Started:** | Introductory notes and installation instructions |
|  | **Examples:** | A list of the examples that are provided in the documentation |
|  | **User guide:** | Describes how to use these tools |
|  | **Function reference:** | A list of the available functions |
|  | **Index** | Alphabetical index of sections, functions and concepts |

  

© 2014 The MathWorks Ltd
• Terms of Use
• Patents
• Trademarks
