# Supplementary material for: Smad4 controls signaling robustness and morphogenesis by differentially contributing to the Nodal and BMP pathways
Source: Nat Commun. 2021 Nov 4;12:6374. doi: 10.1038/s41467-021-26486-3 (PMC8569018; doi:10.1038/s41467-021-26486-3)
Supplement: Supplementary file 10 — Software code [file 41467_2021_26486_MOESM10_ESM.zip › ALYtools-master 08.21/HelperFunctions/GUI Layout Toolbox/layoutdoc/Getting Started1.html]

 


What is GUI Layout Toolbox?


|  |  |
| --- | --- |
| **Getting Started** |  |

## 1: What is GUI Layout Toolbox?

GUI Layout Toolbox provides a package of MATLAB objects that allow for complex
arrangement of graphical user interface elements within a figure window. The
main capabilities provided are:

- Automatic element arrangement horizontally, vertically or in grids
- Ability to specify fixed sizes or resizing weights for each element
- Ability to "nest" layouts to produce virtually any element arrangement
- Divider bars for user-resizing of elements

These element arrangement capabilities are designed to match those found as
standard in other user-interface toolkits such as Java Swing, GTK, QT etc.

Note that this is version 2 of this toolbox, designed to work with
MATLAB releases R2014b and later. For earlier MATLAB releases use
version 1.

  

|  |  |  |  |  |
| --- | --- | --- | --- | --- |
|  | Getting Started | [Top] | Compatibility considerations |  |

© 2014 The MathWorks Ltd
• Terms of Use
• Patents
• Trademarks
