# Supplementary material for: Smad4 controls signaling robustness and morphogenesis by differentially contributing to the Nodal and BMP pathways
Source: Nat Commun. 2021 Nov 4;12:6374. doi: 10.1038/s41467-021-26486-3 (PMC8569018; doi:10.1038/s41467-021-26486-3)
Supplement: Supplementary file 10 — Software code [file 41467_2021_26486_MOESM10_ESM.zip › ALYtools-master 08.21/HelperFunctions/GUI Layout Toolbox/layoutdoc/User guide7_5.html]

Running it


|  |  |
| --- | --- |
| **User guide** |  |

  

## 7.5: Running it

When the main function is launched (click
here)
it first creates the data, then the GUI, then updates the GUI using the data. At this point the
function exits and control is returned to the command prompt. Note, however,
that the GUI is still onscreen and will still respond to user interaction.

This works because the "shared" variables in the main function are not cleared when the
function exits. They are only cleared once the GUI is closed. This is a slightly
unusual, but very useful, feature of using nested functions for building
applications.

### For example:

```
>> demoBrowser
```

(Full source code for this application is available here:
[ view
| edit
| run ]
)

  

|  |  |  |  |  |
| --- | --- | --- | --- | --- |
|  | onListSelection | [Top] | Scalability |  |

© 2014 The MathWorks Ltd
• Terms of Use
• Patents
• Trademarks
