# Supplementary material for: Smad4 controls signaling robustness and morphogenesis by differentially contributing to the Nodal and BMP pathways
Source: Nat Commun. 2021 Nov 4;12:6374. doi: 10.1038/s41467-021-26486-3 (PMC8569018; doi:10.1038/s41467-021-26486-3)
Supplement: Supplementary file 10 — Software code [file 41467_2021_26486_MOESM10_ESM.zip › ALYtools-master 08.21/HelperFunctions/GUI Layout Toolbox/layoutdoc/User guide2.html]

 


Positioning Axes


|  |  |
| --- | --- |
| **User guide** |  |

## 2: Positioning Axes

Unlike other MATLAB user interface components, `axes`
have two position properties: `Position` and
`OuterPosition`. This means one has some extra
options as to how the layout will arrange the axes.

### Section contents:


- 2.1. Position vs OuterPosition
- 2.2. Axes inside layouts
- 2.3. Colorbars and legends

  

|  |  |  |  |  |
| --- | --- | --- | --- | --- |
|  | Why use layouts? | [Top] | Position vs OuterPosition |  |

© 2014 The MathWorks Ltd
• Terms of Use
• Patents
• Trademarks
