# Supplementary material for: Smad4 controls signaling robustness and morphogenesis by differentially contributing to the Nodal and BMP pathways
Source: Nat Commun. 2021 Nov 4;12:6374. doi: 10.1038/s41467-021-26486-3 (PMC8569018; doi:10.1038/s41467-021-26486-3)
Supplement: Supplementary file 10 — Software code [file 41467_2021_26486_MOESM10_ESM.zip › ALYtools-master 08.21/HelperFunctions/GUI Layout Toolbox/layoutdoc/Getting Started6.html]

 


Acknowledgements


|  |  |
| --- | --- |
| **Getting Started** |  |

## 6: Acknowledgements

The authors wish to acknowledge the earlier contributions of the following MathWorks
consultants to this area:

- Brad Phelan
- Malcolm Wood
- Richard Lang
- Paul Kerr-Delworth

  

|  |  |  |  |  |
| --- | --- | --- | --- | --- |
|  | Support | [Top] | Front page |  |

© 2014 The MathWorks Ltd
• Terms of Use
• Patents
• Trademarks
>
