# Supplementary material for: Smad4 controls signaling robustness and morphogenesis by differentially contributing to the Nodal and BMP pathways
Source: Nat Commun. 2021 Nov 4;12:6374. doi: 10.1038/s41467-021-26486-3 (PMC8569018; doi:10.1038/s41467-021-26486-3)
Supplement: Supplementary file 10 — Software code [file 41467_2021_26486_MOESM10_ESM.zip › ALYtools-master 08.21/HelperFunctions/GUI Layout Toolbox/layoutdoc/Function reference3_1.html]

uix.Grid


|  |  |
| --- | --- |
| **Function reference** |  |

  

## 3.1: uix.Grid

  

Arrange elements in a two dimensional grid

`obj` `= uix.Grid(` `)`
:   creates a new new grid layout with all properties set to defaults. The number of rows and
    columns to use is determined from the number of elements in the
    Heights and Widths properties respectively. Child elements are
    arranged down column one first, then column two etc. If there are insufficient
    columns then a new one is added.
    The output is a new layout object that can be used as the parent for other user-interface components.

`obj` `= uix.Grid(` `prop``,` `value``,` `...` `)`
:   also sets one or more property values.

### uix.Grid properties

| Property | Value | Description |
| --- | --- | --- |
| `BackgroundColor` | `colorspec` | The color to use for exposed areas of the layout background. This can be an RGB triple (e.g. [0 0 1]) or a colour name (e.g. 'b'). |
| `BeingDeleted` | [ on \| off ] | is the object in the process of being deleted. |
| `Children` | handle array | List of child objects within this layout (note that this can only be set to permutations of itself). |
| `DeleteFcn` | `function_handle` | Function to call when the layout is being deleted |
| `Enable` | [ on \| off ] | Is interaction with this layout enabled? Note that changing the enable property will cause all children contained to also be enabled/disabled, however since they can be individually re-enabled the state of this property does not reflect the state of the children. See the enable example for more details. |
| `Heights` | double vector | The height of each of the rows. Positive entries indicate fixed sizes in pixels, negative values indicate relative weights for resizing. |
| `Padding` | positive integer | Number of pixels of extra space around the outside of the layout. |
| `Parent` | handle | The handle of the parent figure or container. |
| `Position` | [x y w h] | The position (x,y) and size (w,h) of the layout. |
| `Spacing` | positive integer | Number of pixels of extra space to leave between elements in the layout. |
| `Tag` | string | A string that can be used to identify this object later. |
| `Type` | string | the object type (class). |
| `Units` | [ inches \| centimeters \| normalized \| points \| pixels \| characters ] | The units of measurement for position the layout within its parent. |
| `Visible` | [ on \| off ] | Is the object visible on screen. |
| `Widths` | double vector | The width of each of the columns. Positive entries indicate fixed sizes in pixels, negative values indicate relative weights for resizing. |

### For example:

```
f = figure();
g = uix.Grid( 'Parent', f, 'Spacing', 5 );
uicontrol( 'Parent', g, 'Background', 'r' )
uicontrol( 'Parent', g, 'Background', 'b' )
uicontrol( 'Parent', g, 'Background', 'g' )
uix.Empty( 'Parent', g )
uicontrol( 'Parent', g, 'Background', 'c' )
uicontrol( 'Parent', g, 'Background', 'y' )
set( g, 'Widths', [-1 100 -2], 'Heights', [-1 100] );
```

  
**See also:**

- `uix.GridFlex` - for creating a grid arrangement with draggable dividers

  

|  |  |  |  |  |
| --- | --- | --- | --- | --- |
|  | uix.VButtonBox | [Top] | uix.GridFlex |  |

© 2014 The MathWorks Ltd
• Terms of Use
• Patents
• Trademarks
