# Supplementary material for: Smad4 controls signaling robustness and morphogenesis by differentially contributing to the Nodal and BMP pathways
Source: Nat Commun. 2021 Nov 4;12:6374. doi: 10.1038/s41467-021-26486-3 (PMC8569018; doi:10.1038/s41467-021-26486-3)
Supplement: Supplementary file 10 — Software code [file 41467_2021_26486_MOESM10_ESM.zip › ALYtools-master 08.21/HelperFunctions/GUI Layout Toolbox/layoutdoc/User guide2_1.html]

Position vs OuterPosition


|  |  |
| --- | --- |
| **User guide** |  |

  

## 2.1: Position vs OuterPosition

Typically one
would position some axes using their `OuterPosition` so that the
axis labels, title and other annotations are all contained within the
specified area. Sometimes, particularly if drawing images, one might want
to instead make the axes canvas (the white bit!) fill the specified area.
This is done by setting the `Position` property instead.

### For example:

```
figure
axes( 'Units', 'Normalized', 'OuterPosition', [0 0 1 1] )
```

```
figure
axes( 'Units', 'Normalized', 'Position', [0 0 1 1] )
```

  

|  |  |  |  |  |
| --- | --- | --- | --- | --- |
|  | Positioning Axes | [Top] | Axes inside layouts |  |

© 2014 The MathWorks Ltd
• Terms of Use
• Patents
• Trademarks
