# Supplementary material for: Smad4 controls signaling robustness and morphogenesis by differentially contributing to the Nodal and BMP pathways
Source: Nat Commun. 2021 Nov 4;12:6374. doi: 10.1038/s41467-021-26486-3 (PMC8569018; doi:10.1038/s41467-021-26486-3)
Supplement: Supplementary file 10 — Software code [file 41467_2021_26486_MOESM10_ESM.zip › ALYtools-master 08.21/HelperFunctions/GUI Layout Toolbox/layoutdoc/User guide5.html]

 


Using layouts inside GUIDE GUIs


|  |  |
| --- | --- |
| **User guide** |  |

## 5: Using layouts inside GUIDE GUIs

Whilst the layouts in this toolbox do not integrate into the
MATLAB Graphical User Interface Design Environment (`GUIDE`), it
is possible to insert layouts into a GUIDE-built GUI as follows:

#### Create the GUIDE GUI

Create you GUIDE application as usual, placing a panel where you
want to insert the layout. You can turn the panel border and title
off if you do not want them.

#### Insert the layout

Edit the "OpeningFcn" in the GUIDE-created code and insert your
layout into the panel, making it fill the space. In the example below
four boxpanels are inserted into a grid, which itself is placed inside
`uipanel1`:

```
% --- Executes just before guideApp is made visible. 
function guideApp_OpeningFcn(hObject, eventdata, handles, varargin)

% Choose default command line output for guideApp 
handles.output = hObject;

% Update handles structure 
guidata(hObject, handles);

% Put a layout in the panel 
g = uix.GridFlex( 'Parent', handles.uipanel1, ...
    'Units', 'Normalized', 'Position', [0 0 1 1], ...
    'Spacing', 5 );
uix.BoxPanel( 'Parent', g, 'Title', 'Panel 1' );
uix.BoxPanel( 'Parent', g, 'Title', 'Panel 2' );
uix.BoxPanel( 'Parent', g, 'Title', 'Panel 3' );
uix.BoxPanel( 'Parent', g, 'Title', 'Panel 4' );
g.Heights = [-1 -1];
```

(Full source code for this application is available here:
[ view
| edit
| run ]
)

  

|  |  |  |  |  |
| --- | --- | --- | --- | --- |
|  | Dock and undock | [Top] | Deploying GUIs using the MATLAB Compiler |  |

© 2014 The MathWorks Ltd
• Terms of Use
• Patents
• Trademarks
