# Supplementary material for: Smad4 controls signaling robustness and morphogenesis by differentially contributing to the Nodal and BMP pathways
Source: Nat Commun. 2021 Nov 4;12:6374. doi: 10.1038/s41467-021-26486-3 (PMC8569018; doi:10.1038/s41467-021-26486-3)
Supplement: Supplementary file 10 — Software code [file 41467_2021_26486_MOESM10_ESM.zip › ALYtools-master 08.21/HelperFunctions/GUI Layout Toolbox/layoutdoc/User guide1_4.html]

Layout heirarchies


|  |  |
| --- | --- |
| **User guide** |  |

  

## 1.4: Layout heirarchies

Consider the following simple interface:

Instead of thinking of this in terms of three elements that need
positioning, we can break it up into two simple linear ("box") arrangements:
a vertical box with the axes at the top and a control area at the bottom and
a horizontal box containing two buttons:

.

By placing the second layout (horizontal box) inside the first (vertical box)
we arrive at the complete layout. Since the sizes of the elements in each list
can be set to be fixed or flexible we can achieve a user-interface that is not just
arranged correctly when created, but also behaves well when resized.

Note that the buttons have stayed fixed height as the window grows and the
axes grow to fill the remaining space.

  

---

### 1.4.1: Code:

The example above can be created programmically pretty
much as described in text
(the completed example code is here:
[ view
| edit
| run ]
)

**1. Open a window**

```
f = figure( 'Position', 200*ones(1,4) );
```

**2. Create the first layout (vertical box)**  
Inside this vertical box
we place the axes

```
vbox = uix.VBox( 'Parent', f );
axes( 'Parent', vbox );
```

**3. Create the second layout (horizontal box)**  
Inside this horizontal box
we place two buttons

```
hbox = uix.HButtonBox( 'Parent', vbox, 'Padding', 5 );
uicontrol( 'Parent', hbox, ...
    'String', 'Button 1' );
uicontrol( 'Parent', hbox, ...
    'String', 'Button 2' );
```

**4. Set the sizes**  
We want the axes to grow with the window so
set the first size to be -1 (which means variable size with wieght 1) and the buttons to stay fixed height so set the
second size to 35 (fixed height of 35 pixels)

```
set( vbox, 'Heights', [-1 35] )
```

  

|  |  |  |  |  |
| --- | --- | --- | --- | --- |
|  | Sizes and units | [Top] | Why use layouts? |  |

© 2014 The MathWorks Ltd
• Terms of Use
• Patents
• Trademarks
