# Supplementary material for: Smad4 controls signaling robustness and morphogenesis by differentially contributing to the Nodal and BMP pathways
Source: Nat Commun. 2021 Nov 4;12:6374. doi: 10.1038/s41467-021-26486-3 (PMC8569018; doi:10.1038/s41467-021-26486-3)
Supplement: Supplementary file 10 — Software code [file 41467_2021_26486_MOESM10_ESM.zip › ALYtools-master 08.21/HelperFunctions/GUI Layout Toolbox/layoutdoc/helptoc.html]

Table of Contents


 **GUI Layout**  
  
  **Getting Started**  
     1. What is GUI Layout Toolbox?  
     2. Compatibility considerations  
     3. Release notes  
     4. Installation  
     5. Support  
     6. Acknowledgements  
  
  **Examples**  
  
  **User guide**  
     1. Understanding layouts  
     2. Positioning Axes  
     3. Controlling visibility  
     4. Advanced maneuvers with panels  
     5. Using layouts inside GUIDE GUIs  
     6. Deploying GUIs using the MATLAB Compiler  
     7. A complete example  
  
  **Function reference**  
  
  **Index**
