# Supplementary material for: Smad4 controls signaling robustness and morphogenesis by differentially contributing to the Nodal and BMP pathways
Source: Nat Commun. 2021 Nov 4;12:6374. doi: 10.1038/s41467-021-26486-3 (PMC8569018; doi:10.1038/s41467-021-26486-3)
Supplement: Supplementary file 10 — Software code [file 41467_2021_26486_MOESM10_ESM.zip › ALYtools-master 08.21/HelperFunctions/GUI Layout Toolbox/layoutdoc/User guide1_5.html]

Why use layouts?


|  |  |
| --- | --- |
| **User guide** |  |

  

## 1.5: Why use layouts?

MATLAB ships with a GUI design tool called `GUIDE`. This
doesn't use layouts, but forces users to manually position each element. This approach
is a much faster way to build simple user-interfaces, so why would you want to
use layouts?

The over-riding reason for using layouts or layout managers is
to gain control of the resizing behaviour of the interface without
having to write a complex "ResizeFcn". If you simply position user-interface elements
directly (either using `GUIDE` or programmatically), you
have two choices about what happens when the window resizes:

### For example:

**1. The user-interface components scale with the window (normalised units)**  
We didn't really want the buttons to grow but everything resizes in proportion.

```
f = figure( 'Position', 200*ones(1,4) );
axes( 'Parent', f, ...
    'Units', 'Normalized', ...
    'OuterPosition', [0.02 0.2 0.96 0.8] );
uicontrol( 'Parent', f, ...
    'Units', 'Normalized', ...
    'Position', [0.02 0.02 0.46 0.16], ...
    'String', 'Button 1' );
uicontrol( 'Parent', f, ...
    'Units', 'Normalized', ...
    'Position', [0.52 0.02 0.46 0.16], ...
    'String', 'Button 2' );
```

.

**2. The user-interface components stay fixed and the window resize creates empty space (pixel units)**  
Although the buttons don't now grow, neither does the axes, which looks very odd.

```
f = figure( 'Position', 200*ones(1,4) );
axes( 'Parent', f, ...
    'Units', 'Pixels', ...
    'OuterPosition', [10 35 190 175] );
uicontrol( 'Parent', f, ...
    'Units', 'Pixels', ...
    'Position', [5 5 90 25], ...
    'String', 'Button 1' );
uicontrol( 'Parent', f, ...
    'Units', 'Pixels', ...
    'Position', [105 5 90 25], ...
    'String', 'Button 2' );
```

.

Neither of these alternatives is particularly useful for a serious
user-interface. Typically there are user-interface components that should
be fixed size: icons, buttons, selectors etc; and others that should resize
with the window: graphs, images, prose text etc. To achieve this one needs
to be able to specify which interface components should be fixed size and
which variable. Over the last two decades, layouts have been shown to be
the method of choice for achieving this.

### For example:

Using layouts, some user-interface components scale with the window, others stay fixed

```
f = figure( 'Position', 200*ones(1,4) );
vbox = uix.VBox( 'Parent', f );
axes( 'Parent', vbox );
hbox = uix.HButtonBox( 'Parent', vbox, 'Padding', 5 );
uicontrol( 'Parent', hbox, ...
    'String', 'Button 1' );
uicontrol( 'Parent', hbox, ...
    'String', 'Button 2' );
set( vbox, 'Heights', [-1 35] )
```

.

  

|  |  |  |  |  |
| --- | --- | --- | --- | --- |
|  | Layout heirarchies | [Top] | Positioning Axes |  |

© 2014 The MathWorks Ltd
• Terms of Use
• Patents
• Trademarks
