# Supplementary material for: Smad4 controls signaling robustness and morphogenesis by differentially contributing to the Nodal and BMP pathways
Source: Nat Commun. 2021 Nov 4;12:6374. doi: 10.1038/s41467-021-26486-3 (PMC8569018; doi:10.1038/s41467-021-26486-3)
Supplement: Supplementary file 10 — Software code [file 41467_2021_26486_MOESM10_ESM.zip › ALYtools-master 08.21/HelperFunctions/GUI Layout Toolbox/layoutdoc/User guide4.html]

 


Advanced maneuvers with panels


|  |  |
| --- | --- |
| **User guide** |  |

## 4: Advanced maneuvers with panels

The `uix.BoxPanel` provides some extra buttons
and callbacks that can be used to design advanced user-interface
layouts. In particular, the sections below illustrate how to create
interfaces with context help, and panels that can be minimized or undocked.

### Section contents:


- 4.1. Context help
- 4.2. Minimize and maximize
- 4.3. Dock and undock

  

|  |  |  |  |  |
| --- | --- | --- | --- | --- |
|  | Visible example | [Top] | Context help |  |

© 2014 The MathWorks Ltd
• Terms of Use
• Patents
• Trademarks
