# Supplementary material for: Smad4 controls signaling robustness and morphogenesis by differentially contributing to the Nodal and BMP pathways
Source: Nat Commun. 2021 Nov 4;12:6374. doi: 10.1038/s41467-021-26486-3 (PMC8569018; doi:10.1038/s41467-021-26486-3)
Supplement: Supplementary file 10 — Software code [file 41467_2021_26486_MOESM10_ESM.zip › ALYtools-master 08.21/HelperFunctions/GUI Layout Toolbox/layoutdoc/Function reference4_2.html]

layoutRoot


|  |  |
| --- | --- |
| **Function reference** |  |

  

## 4.2: layoutRoot

returns the folder containing the GUI layout toolbox

`folder` `= layoutRoot(` `)`
:   returns the full path to the folder containing the GUI Layout Toolbox.

### For example:

```
folder = layoutRoot()
```

`folder = 'C:\Temp\LayoutToolbox1.0'`

  

|  |  |  |  |  |
| --- | --- | --- | --- | --- |
|  | uix.Empty | [Top] | Front page |  |

© 2014 The MathWorks Ltd
• Terms of Use
• Patents
• Trademarks
>
