# Supplementary material for: Smad4 controls signaling robustness and morphogenesis by differentially contributing to the Nodal and BMP pathways
Source: Nat Commun. 2021 Nov 4;12:6374. doi: 10.1038/s41467-021-26486-3 (PMC8569018; doi:10.1038/s41467-021-26486-3)
Supplement: Supplementary file 10 — Software code [file 41467_2021_26486_MOESM10_ESM.zip › ALYtools-master 08.21/HelperFunctions/GUI Layout Toolbox/layoutdoc/Function reference1_2.html]

uix.CardPanel


|  |  |
| --- | --- |
| **Function reference** |  |

  

## 1.2: uix.CardPanel

  

Show one element (card) from a list

`obj` `= uix.CardPanel(` `)`
:   creates a new card panel which allows
    selection between the different child objects contained, making the
    selected child fill the space available and all other children
    invisible. This is commonly used for creating wizards or quick
    switching between different views of a single data-set.

`obj` `= uix.CardPanel(` `prop``,` `value``,` `...` `)`
:   also sets one or more property values.

### uix.CardPanel properties

| Property | Value | Description |
| --- | --- | --- |
| `BackgroundColor` | `colorspec` | The color to use for exposed areas of the layout background. This can be an RGB triple (e.g. [0 0 1]) or a colour name (e.g. 'b'). |
| `BeingDeleted` | [ on \| off ] | is the object in the process of being deleted. |
| `Children` | handle array | List of child objects within this layout (note that this can only be set to permutations of itself). |
| `DeleteFcn` | `function_handle` | Function to call when the layout is being deleted |
| `Enable` | [ on \| off ] | Is interaction with this layout enabled? Note that changing the enable property will cause all children contained to also be enabled/disabled, however since they can be individually re-enabled the state of this property does not reflect the state of the children. See the enable example for more details. |
| `Padding` | positive integer | Number of pixels of extra space around the outside of the layout. |
| `Parent` | handle | The handle of the parent figure or container. |
| `Position` | [x y w h] | The position (x,y) and size (w,h) of the layout. |
| `Selection` | positive integer or empty | Which child is visible. |
| `Tag` | string | A string that can be used to identify this object later. |
| `Type` | string | the object type (class). |
| `Units` | [ inches \| centimeters \| normalized \| points \| pixels \| characters ] | The units of measurement for position the layout within its parent. |
| `Visible` | [ on \| off ] | Is the object visible on screen. |

### For example:

```
f = figure();
p = uix.CardPanel( 'Parent', f, 'Padding', 5 );
uicontrol( 'Parent', p, 'Background', 'r' );
uicontrol( 'Parent', p, 'Background', 'b' );
uicontrol( 'Parent', p, 'Background', 'g' );
p.Selection = 2;
```

  
**See also:**

- `uix.Panel` - for creating a standard panel
- `uix.BoxPanel` - for creating a more boxy panel
- `uix.TabPanel` - for a panel with tabs for selecting the visible child

  

|  |  |  |  |  |
| --- | --- | --- | --- | --- |
|  | uix.Panel | [Top] | uix.BoxPanel |  |

© 2014 The MathWorks Ltd
• Terms of Use
• Patents
• Trademarks
