# Supplementary material for: Smad4 controls signaling robustness and morphogenesis by differentially contributing to the Nodal and BMP pathways
Source: Nat Commun. 2021 Nov 4;12:6374. doi: 10.1038/s41467-021-26486-3 (PMC8569018; doi:10.1038/s41467-021-26486-3)
Supplement: Supplementary file 10 — Software code [file 41467_2021_26486_MOESM10_ESM.zip › ALYtools-master 08.21/HelperFunctions/GUI Layout Toolbox/layoutdoc/Function reference4_1.html]

uix.Empty


|  |  |
| --- | --- |
| **Function reference** |  |

  

## 4.1: uix.Empty

Create an empty space

`obj` `= uix.Empty(` `)`
:   creates an empty space object that can be
    used in layouts to add gaps between other elements.

`obj` `= uix.Empty(` `param``,` `value``,` `...` `)`
:   also sets one or more property values.

### uix.Empty properties

| Property | Value | Description |
| --- | --- | --- |
| `BeingDeleted` | [ on \| off ] | is the object in the process of being deleted. |
| `Parent` | handle | The handle of the parent figure or container. |
| `Position` | [x y w h] | The position (x,y) and size (w,h) of the layout. |
| `Tag` | string | A string that can be used to identify this object later. |
| `Type` | string | the object type (class). |
| `Units` | [ inches \| centimeters \| normalized \| points \| pixels \| characters ] | The units of measurement for position the layout within its parent. |
| `Visible` | [ on \| off ] | Is the object visible on screen. |

### For example:

```
f = figure();
box = uix.HBox( 'Parent', f, 'Spacing', 5 );
uicontrol( 'Parent', box, 'Background', 'r' )
uix.Empty( 'Parent', box )
uicontrol( 'Parent', box, 'Background', 'g' )
```

  
**See also:**

- `uix.HBox` - for arranging widgets horizontally

  

|  |  |  |  |  |
| --- | --- | --- | --- | --- |
|  | uix.GridFlex | [Top] | layoutRoot |  |

© 2014 The MathWorks Ltd
• Terms of Use
• Patents
• Trademarks
