# Supplementary material for: Smad4 controls signaling robustness and morphogenesis by differentially contributing to the Nodal and BMP pathways
Source: Nat Commun. 2021 Nov 4;12:6374. doi: 10.1038/s41467-021-26486-3 (PMC8569018; doi:10.1038/s41467-021-26486-3)
Supplement: Supplementary file 10 — Software code [file 41467_2021_26486_MOESM10_ESM.zip › ALYtools-master 08.21/HelperFunctions/GUI Layout Toolbox/layoutdoc/User guide.html]

User guide


|  |  |
| --- | --- |
| **GUI Layout Toolbox 2.1** |  |

# User guide

This chapter describes the purpose and overall design of the layout
tools that the toolbox provides. It also provides some examples of
creating some user interfaces using layouts.


  


## Contents

- 1. Understanding layouts
- 2. Positioning Axes
- 3. Controlling visibility
- 4. Advanced maneuvers with panels
- 5. Using layouts inside GUIDE GUIs
- 6. Deploying GUIs using the MATLAB Compiler
- 7. A complete example

  

|  |  |  |  |  |
| --- | --- | --- | --- | --- |
|  | Front page | [Top] | Understanding layouts |  |

© 2014 The MathWorks Ltd
• Terms of Use
• Patents
• Trademarks
