# Supplementary material for: Smad4 controls signaling robustness and morphogenesis by differentially contributing to the Nodal and BMP pathways
Source: Nat Commun. 2021 Nov 4;12:6374. doi: 10.1038/s41467-021-26486-3 (PMC8569018; doi:10.1038/s41467-021-26486-3)
Supplement: Supplementary file 10 — Software code [file 41467_2021_26486_MOESM10_ESM.zip › ALYtools-master 08.21/HelperFunctions/GUI Layout Toolbox/layoutdoc/User guide7.html]

 


A complete example


|  |  |
| --- | --- |
| **User guide** |  |

## 7: A complete example

The following example application uses many of the layout features
discussed above in order to create a good-looking user interface that
scales well when resized. It is not designed to showcase all the
layout functionality, but shows how callbacks are added to provide
user interaction. It also exemplifies separating the data from the GUI,
a fundamental part of creating modular and maintainable applications.

The full application is available here:

- View demoBrowser.m
- Edit demoBrowser.m
- Run demoBrowser.m

### Section contents:


- 7.1. Application structure
- 7.2. createInterface
- 7.3. updateInterface
- 7.4. onListSelection
- 7.5. Running it
- 7.6. Scalability

  

|  |  |  |  |  |
| --- | --- | --- | --- | --- |
|  | Deploying GUIs using the MATLAB Compiler | [Top] | Application structure |  |

© 2014 The MathWorks Ltd
• Terms of Use
• Patents
• Trademarks
