# Supplementary material for: Smad4 controls signaling robustness and morphogenesis by differentially contributing to the Nodal and BMP pathways
Source: Nat Commun. 2021 Nov 4;12:6374. doi: 10.1038/s41467-021-26486-3 (PMC8569018; doi:10.1038/s41467-021-26486-3)
Supplement: Supplementary file 10 — Software code [file 41467_2021_26486_MOESM10_ESM.zip › ALYtools-master 08.21/HelperFunctions/GUI Layout Toolbox/layoutdoc/Function reference.html]

Function reference


|  |  |
| --- | --- |
| **GUI Layout Toolbox 2.1** |  |

# Function reference


|  |  |
| --- | --- |
| 1: Panels | |
| 1.1 uix.Panel | Arrange a single element inside a standard panel |
| 1.2 uix.CardPanel | Show one element (card) from a list |
| 1.3 uix.BoxPanel | Arrange a single element in a panel with boxed title |
| 1.4 uix.TabPanel | Arrange elements in a panel with tabs for selecting which is visible |
| 2: Boxes | |
| 2.1 uix.HBox | Arrange elements horizontally in a single row |
| 2.2 uix.VBox | Arrange elements vertically in a single column |
| 2.3 uix.HBoxFlex | Arrange elements horizontally with draggable dividers |
| 2.4 uix.VBoxFlex | Arrange elements vertically with draggable dividers |
| 2.5 uix.HButtonBox | Arrange buttons horizontally in a single row |
| 2.6 uix.VButtonBox | Arrange buttons vertically in a single column |
| 3: Grids | |
| 3.1 uix.Grid | Arrange elements in a two dimensional grid |
| 3.2 uix.GridFlex | Arrange elements in a two dimensional grid with draggable dividers |
| 4: Other functions | |
| 4.1 uix.Empty | Create an empty space |
| 4.2 layoutRoot | Return the folder containing the GUI layout toolbox |

  
  

|  |  |  |  |  |
| --- | --- | --- | --- | --- |
|  | Front page | [Top] | uix.Panel |  |

© 2014 The MathWorks Ltd
• Terms of Use
• Patents
• Trademarks
