# Supplementary material for: Smad4 controls signaling robustness and morphogenesis by differentially contributing to the Nodal and BMP pathways
Source: Nat Commun. 2021 Nov 4;12:6374. doi: 10.1038/s41467-021-26486-3 (PMC8569018; doi:10.1038/s41467-021-26486-3)
Supplement: Supplementary file 10 — Software code [file 41467_2021_26486_MOESM10_ESM.zip › ALYtools-master 08.21/HelperFunctions/GUI Layout Toolbox/layoutdoc/User guide6.html]

 


Deploying GUIs using the MATLAB Compiler


|  |  |
| --- | --- |
| **User guide** |  |

## 6: Deploying GUIs using the MATLAB Compiler

Applications built using these layout tools can be deployed as standalone
executables using the MATLAB Compiler in
the same way as any other MATLAB application. There is, however, one thing to watch out for:

- You must explicitly include the "Resources" folder within the +uix package

#### Example

Below is a screen-shot of the `deploytool` setup
used to build the example application.
The "Resources" folder from the toolbox has been explicitly added as a
shared resource so that the mouse-pointers and panel icons continue to work.


  

|  |  |  |  |  |
| --- | --- | --- | --- | --- |
|  | Using layouts inside GUIDE GUIs | [Top] | A complete example |  |

© 2014 The MathWorks Ltd
• Terms of Use
• Patents
• Trademarks
