# Supplementary material for: Smad4 controls signaling robustness and morphogenesis by differentially contributing to the Nodal and BMP pathways
Source: Nat Commun. 2021 Nov 4;12:6374. doi: 10.1038/s41467-021-26486-3 (PMC8569018; doi:10.1038/s41467-021-26486-3)
Supplement: Supplementary file 10 — Software code [file 41467_2021_26486_MOESM10_ESM.zip › ALYtools-master 08.21/HelperFunctions/GUI Layout Toolbox/layoutdoc/User guide7_1.html]

Application structure


|  |  |
| --- | --- |
| **User guide** |  |

  

## 7.1: Application structure

There are many ways to build graphical applications in MATLAB, but
here we will take a very simple approach. If the application were to become
larger and more complex, this approach would be changed to better mitigate
the complexity. Some notes on this are contained at the end.

The application is structured as a single function with callbacks and other helper
functions stored as "nested" subfunctions, i.e. functions inside the main function. This has
the advantage that the nested subfunctions can share access to any variables
declared in the main function. This is also a risk as anything we accidentally
declare in the main function becomes "global" within the application. For that reason
*all* logic is put into subfunctions and we restrict the main
function to just declaring two shared variables:

- **data**: a structure containing all shared data
- **gui**: a structure containing handles to GUI widgets

```
function demoBrowser()

   % Declare shared variables
   data = createData();
   gui = createInterface( data.DemoNames );

   % Now update the GUI with the current data
   updateInterface();
   redrawDemo();
   
   % Helper subfunctions.
   function data = createData() ... end;
   function gui = createInterface(names) ... end;
   function updateInterface() ... end;
   function redrawDemo() ... end;

   % Callback subfunctions.
   function onMenuSelection() ... end;
   function onListSelection() ... end;
   function onDemoHelp() ... end;
   function onHelp() ... end;
   function onExit() ... end;

end  % Main function
```

Note that all of the work is done in subfunctions. Most subfunctions
are callbacks executed when a button is pressed or a menu selected. The four used
at startup are helper functions:

- **createData**: build the structure which contains all application data
- **createInterface**: build the user interface
- **updateInterface**: update selectors etc in response to a change in the data
- **redrawDemo**: redraw the plot part of the interface

We will not dig into all the subfunctions and callbacks, but instead
concentrate on the GUI creation (createInterface)
and update (updateInterface).

(Full source code for this application is available here:
[ view
| edit
| run ]
)

  

|  |  |  |  |  |
| --- | --- | --- | --- | --- |
|  | A complete example | [Top] | createInterface |  |

© 2014 The MathWorks Ltd
• Terms of Use
• Patents
• Trademarks
