# Supplementary material for: Smad4 controls signaling robustness and morphogenesis by differentially contributing to the Nodal and BMP pathways
Source: Nat Commun. 2021 Nov 4;12:6374. doi: 10.1038/s41467-021-26486-3 (PMC8569018; doi:10.1038/s41467-021-26486-3)
Supplement: Supplementary file 10 — Software code [file 41467_2021_26486_MOESM10_ESM.zip › ALYtools-master 08.21/HelperFunctions/GUI Layout Toolbox/layoutdoc/Function reference2_6.html]

uix.VButtonBox


|  |  |
| --- | --- |
| **Function reference** |  |

  

## 2.6: uix.VButtonBox

  

Arrange buttons vertically in a single column

`obj` `= uix.VButtonBox(` `)`
:   is a type of VBox specialised for
    arranging a column of buttons, check-boxes or similar graphical
    elements. All buttons are given equal size and by default are
    centered in the drawing area. The justification can be changed as
    required.

`obj` `= uix.VButtonBox(` `prop``,` `value``,` `...` `)`
:   also sets one or more property values.

### uix.VButtonBox properties

| Property | Value | Description |
| --- | --- | --- |
| `BackgroundColor` | `colorspec` | The color to use for exposed areas of the layout background. This can be an RGB triple (e.g. [0 0 1]) or a colour name (e.g. 'b'). |
| `BeingDeleted` | [ on \| off ] | is the object in the process of being deleted. |
| `ButtonSize` | [w h] | The size for the buttons (all are given equal size). |
| `Children` | handle array | List of child objects within this layout (note that this can only be set to permutations of itself). |
| `DeleteFcn` | `function_handle` | Function to call when the layout is being deleted |
| `Enable` | [ on \| off ] | Is interaction with this layout enabled? Note that changing the enable property will cause all children contained to also be enabled/disabled, however since they can be individually re-enabled the state of this property does not reflect the state of the children. See the enable example for more details. |
| `HorizontalAlignment` | [ left \| center \| right ] | The horizontal position of the buttons. |
| `Padding` | positive integer | Number of pixels of extra space around the outside of the layout. |
| `Parent` | handle | The handle of the parent figure or container. |
| `Position` | [x y w h] | The position (x,y) and size (w,h) of the layout. |
| `Spacing` | positive integer | Number of pixels of extra space to leave between elements in the layout. |
| `Tag` | string | A string that can be used to identify this object later. |
| `Type` | string | the object type (class). |
| `Units` | [ inches \| centimeters \| normalized \| points \| pixels \| characters ] | The units of measurement for position the layout within its parent. |
| `VerticalAlignment` | [ top \| middle \| bottom ] | The vertical position of the buttons. |
| `Visible` | [ on \| off ] | Is the object visible on screen. |

### For example:

```
f = figure();
b = uix.VButtonBox( 'Parent', f );
uicontrol( 'Parent', b, 'String', 'One' );
uicontrol( 'Parent', b, 'String', 'Two' );
uicontrol( 'Parent', b, 'String', 'Three' );
set( b, 'ButtonSize', [130 35], 'Spacing', 5 );
```

  
**See also:**

- `uix.HButtonBox` - for creating a horizontal arrangement of buttons
- `uix.VBox` - for creating a general vertical arrangement

  

|  |  |  |  |  |
| --- | --- | --- | --- | --- |
|  | uix.HButtonBox | [Top] | uix.Grid |  |

© 2014 The MathWorks Ltd
• Terms of Use
• Patents
• Trademarks
