# Supplementary material for: Smad4 controls signaling robustness and morphogenesis by differentially contributing to the Nodal and BMP pathways
Source: Nat Commun. 2021 Nov 4;12:6374. doi: 10.1038/s41467-021-26486-3 (PMC8569018; doi:10.1038/s41467-021-26486-3)
Supplement: Supplementary file 10 — Software code [file 41467_2021_26486_MOESM10_ESM.zip › ALYtools-master 08.21/HelperFunctions/GUI Layout Toolbox/layoutdoc/Getting Started5.html]

 


Support


|  |  |
| --- | --- |
| **Getting Started** |  |

## 5: Support

This toolbox is not a MathWorks supported product. However, if you
have problems, suggestions or other comments, please contact the
authors:

- David Sampson
- Ben Tordoff

If you like this toolbox, help others to find it by leaving a rating
and comment on the MATLAB Central File Exchange.

  

|  |  |  |  |  |
| --- | --- | --- | --- | --- |
|  | Installation | [Top] | Acknowledgements |  |

© 2014 The MathWorks Ltd
• Terms of Use
• Patents
• Trademarks
