# Supplementary material for: Smad4 controls signaling robustness and morphogenesis by differentially contributing to the Nodal and BMP pathways
Source: Nat Commun. 2021 Nov 4;12:6374. doi: 10.1038/s41467-021-26486-3 (PMC8569018; doi:10.1038/s41467-021-26486-3)
Supplement: Supplementary file 10 — Software code [file 41467_2021_26486_MOESM10_ESM.zip › ALYtools-master 08.21/HelperFunctions/GUI Layout Toolbox/layoutdoc/Function reference1_3.html]

uix.BoxPanel


|  |  |
| --- | --- |
| **Function reference** |  |

  

## 1.3: uix.BoxPanel

  

Arrange a single element in a panel with boxed title

`obj` `= uix.BoxPanel(` `)`
:   creates a box-styled panel object with
    automatic management of the contained widget or layout. The
    properties available are largely the same as the builtin `UIPANEL`
    object. Where more than one child is added, the currently visible
    child is determined using the Selection property.

`obj` `= uix.BoxPanel(` `prop``,` `value``,` `...` `)`
:   also sets one or more property values.

### uix.BoxPanel properties

| Property | Value | Description |
| --- | --- | --- |
| `BackgroundColor` | `colorspec` | The color to use for exposed areas of the layout background. This can be an RGB triple (e.g. [0 0 1]) or a colour name (e.g. 'b'). |
| `BeingDeleted` | [ on \| off ] | is the object in the process of being deleted. |
| `BorderType` | [ none \| etchedin \| etchedout \| beveledin \| beveledout \| line ] | Type of border around the title and content areas. |
| `Children` | handle array | List of child objects within this layout (note that this can only be set to permutations of itself). |
| `CloseRequestFcn` | `function_handle` | Function to call when the panel close icon is clicked. Note that if empty, no close button is shown. |
| `DeleteFcn` | `function_handle` | Function to call when the layout is being deleted |
| `DockFcn` | function handle | Function to call when panel is docked or undocked. Note that if empty, no dock button is shown. See advanced manoeuvres with panels for details. |
| `Enable` | [ on \| off ] | Is interaction with this layout enabled? Note that changing the enable property will cause all children contained to also be enabled/disabled, however since they can be individually re-enabled the state of this property does not reflect the state of the children. See the enable example for more details. |
| `FontAngle` | [ normal \| italic \| oblique ] | Title font angle. |
| `FontName` | string | Title font name (e.g. Arial, Helvetica etc). |
| `FontSize` | positive integer | Title font size. |
| `FontUnits` | [ inches \| centimeters \| normalized \| points \| pixels ] | Title font units for measuring size. |
| `FontWeight` | [ light \| normal \| demi \| bold ] | Title font weight. |
| `ForegroundColor` | `colorspec` | Title font color and/or color of 2-D border line. |
| `HelpFcn` | function handle | Function to call when the help icon is clicked. Note that if empty, no help button is shown. See advanced manoeuvres with panels for details. |
| `HighlightColor` | `colorspec` | 3-D frame highlight color. |
| `IsDocked` | logical | Is this panel in a docked state. See advanced manoeuvres with panels for details. |
| `IsMinimized` | logical | Is this panel in a minimized state. See advanced manoeuvres with panels for details. |
| `MinimizeFcn` | function handle | Function to call when panel is minimized or maximized. Note that if empty, no minimize button is shown. See advanced manoeuvres with panels for details. |
| `Padding` | positive integer | Number of pixels of extra space around the outside of the layout. |
| `Parent` | handle | The handle of the parent figure or container. |
| `Position` | [x y w h] | The position (x,y) and size (w,h) of the layout. |
| `Selection` | positive integer or empty | Which child is visible. |
| `ShadowColor` | `colorspec` | 3-D frame shadow color. |
| `Tag` | string | A string that can be used to identify this object later. |
| `Title` | string | Title string. |
| `TitleColor` | `colorspec` | color for the title bar background. |
| `Type` | string | the object type (class). |
| `Units` | [ inches \| centimeters \| normalized \| points \| pixels \| characters ] | The units of measurement for position the layout within its parent. |
| `Visible` | [ on \| off ] | Is the object visible on screen. See the visible example for more details. |

### For example:

```
f = figure();
p = uix.BoxPanel( 'Parent', f, 'Title', 'A BoxPanel', 'Padding', 5 );
uicontrol( 'Parent', p, 'Background', 'r' )
```

```
f = figure();
p = uix.BoxPanel( 'Parent', f, 'Title', 'A BoxPanel', 'Padding', 5 );
b = uix.HBox( 'Parent', p, 'Spacing', 5, 'Padding', 5  );
uicontrol( 'Style', 'listbox', 'Parent', b, 'String', {'Item 1','Item 2'} );
uicontrol( 'Parent', b, 'Background', 'b' );
set( b, 'Widths', [100 -1] );
p.FontSize = 12;
p.FontWeight = 'bold';
p.HelpFcn = @(x,y) disp('Help me!');
```

  
**See also:**

- `uix.Panel` - for creating a more standard panel
- `uix.TabPanel` - for a panel with tabs for selecting the visible child
- `uix.HBox` - for creating a horizontal arrangement within a panel

  

|  |  |  |  |  |
| --- | --- | --- | --- | --- |
|  | uix.CardPanel | [Top] | uix.TabPanel |  |

© 2014 The MathWorks Ltd
• Terms of Use
• Patents
• Trademarks
