# Supplementary material for: Smad4 controls signaling robustness and morphogenesis by differentially contributing to the Nodal and BMP pathways
Source: Nat Commun. 2021 Nov 4;12:6374. doi: 10.1038/s41467-021-26486-3 (PMC8569018; doi:10.1038/s41467-021-26486-3)
Supplement: Supplementary file 10 — Software code [file 41467_2021_26486_MOESM10_ESM.zip › ALYtools-master 08.21/HelperFunctions/GUI Layout Toolbox/layoutdoc/atoz.html]

GUI Layout Toolbox documentation: Index
  


GUI Layout Toolbox 2.1

# Alphabetical index

A -
B -
C -
D -
E -
F -
G -
H -
I -
J -
K -
L -
M
N -
O -
P -
Q -
R -
S -
T -
U -
V -
W -
X -
Y -
Z

|  |  |  |  |  |  |  |  |  |  |  |  |  |  |  |  |  |  |  |  |  |  |  |  |  |  |  |  |  |  |  |  |  |  |  |  |  |  |  |  |  |  |  |  |  |  |  |  |  |  |  |  |  |  |  |  |  |  |  |  |  |  |  |  |  |  |  |  |  |  |  |  |  |  |  |  |  |  |  |  |  |  |  |  |  |  |  |  |  |  |  |  |  |  |  |  |  |  |  |  |  |  |  |  |  |  |  |  |  |  |  |  |  |  |  |  |  |  |  |  |  |  |  |  |  |  |  |  |  |  |  |  |  |  |  |  |  |  |  |  |  |  |  |  |  |  |  |  |  |  |  |  |  |  |  |  |  |  |  |  |  |  |  |  |  |  |  |  |  |  |  |  |  |  |  |  |  |  |  |  |  |  |  |  |  |  |  |  |  |  |  |  |  |  |  |  |  |  |  |  |  |  |  |  |  |  |  |  |  |  |  |  |  |  |  |  |  |  |  |  |  |  |  |  |  |  |  |  |  |  |  |  |  |  |  |  |  |  |  |  |  |  |  |  |  |  |  |
| --- | --- | --- | --- | --- | --- | --- | --- | --- | --- | --- | --- | --- | --- | --- | --- | --- | --- | --- | --- | --- | --- | --- | --- | --- | --- | --- | --- | --- | --- | --- | --- | --- | --- | --- | --- | --- | --- | --- | --- | --- | --- | --- | --- | --- | --- | --- | --- | --- | --- | --- | --- | --- | --- | --- | --- | --- | --- | --- | --- | --- | --- | --- | --- | --- | --- | --- | --- | --- | --- | --- | --- | --- | --- | --- | --- | --- | --- | --- | --- | --- | --- | --- | --- | --- | --- | --- | --- | --- | --- | --- | --- | --- | --- | --- | --- | --- | --- | --- | --- | --- | --- | --- | --- | --- | --- | --- | --- | --- | --- | --- | --- | --- | --- | --- | --- | --- | --- | --- | --- | --- | --- | --- | --- | --- | --- | --- | --- | --- | --- | --- | --- | --- | --- | --- | --- | --- | --- | --- | --- | --- | --- | --- | --- | --- | --- | --- | --- | --- | --- | --- | --- | --- | --- | --- | --- | --- | --- | --- | --- | --- | --- | --- | --- | --- | --- | --- | --- | --- | --- | --- | --- | --- | --- | --- | --- | --- | --- | --- | --- | --- | --- | --- | --- | --- | --- | --- | --- | --- | --- | --- | --- | --- | --- | --- | --- | --- | --- | --- | --- | --- | --- | --- | --- | --- | --- | --- | --- | --- | --- | --- | --- | --- | --- | --- | --- | --- | --- | --- | --- | --- | --- | --- | --- | --- | --- | --- | --- | --- | --- | --- | --- | --- | --- | --- | --- | --- | --- | --- | --- | --- | --- | --- | --- | --- | --- | --- |
| \|  \|  \| \| --- \| --- \| \| ---   A \| \| \| A complete example \| A complete example (User guide: 7) \| \| About \| What is GUI Layout Toolbox? (Getting Started: 1) \| \| Acknowledgements \| Acknowledgements (Getting Started: 6) \| \| Advanced maneuvers with panels \| Advanced maneuvers with panels (User guide: 4) \| \| Application design \| A complete example (User guide: 7) \| \| Application structure \| Application structure (User guide: 7.1) \| \| axes \| Positioning Axes (User guide: 2) \| \| Axes inside layouts \| Axes inside layouts (User guide: 2.2) \| \| ---   B \| \| \| Box \| uix.HBox (Function reference: 2.1) \| \|  \| uix.VBox (Function reference: 2.2) \| \|  \| uix.HBoxFlex (Function reference: 2.3) \| \|  \| uix.VBoxFlex (Function reference: 2.4) \| \| Boxes \| Boxes (Function reference: 2) \| \| BoxPanel \| uix.BoxPanel (Function reference: 1.3) \| \| Button box \| uix.HButtonBox (Function reference: 2.5) \| \|  \| uix.VButtonBox (Function reference: 2.6) \| \| ---   C \| \| \| Callbacks \| A complete example (User guide: 7) \| \| CardPanel \| uix.CardPanel (Function reference: 1.2) \| \| Colorbars and legends \| Colorbars and legends (User guide: 2.3) \| \| Compatibility considerations \| Compatibility considerations (Getting Started: 2) \| \| Compiler \| Deploying GUIs using the MATLAB Compiler (User guide: 6) \| \| Context help \| Context help (User guide: 4.1) \| \| Controlling visibility \| Controlling visibility (User guide: 3) \| \| createInterface \| createInterface (User guide: 7.2) \| \| ---   D \| \| \| Deploying GUIs using the MATLAB Compiler \| Deploying GUIs using the MATLAB Compiler (User guide: 6) \| \| Dock and undock \| Dock and undock (User guide: 4.3) \| \| Draggable dividers \| uix.HBoxFlex (Function reference: 2.3) \| \|  \| uix.VBoxFlex (Function reference: 2.4) \| \|  \| uix.GridFlex (Function reference: 3.2) \| \| ---   E \| \| \| Empty \| uix.Empty (Function reference: 4.1) \| \| Examples \| (Examples) \| \| ---   F \| \| \| Flexible layout \| uix.HBoxFlex (Function reference: 2.3) \| \|  \| uix.VBoxFlex (Function reference: 2.4) \| \| Folder \| layoutRoot (Function reference: 4.2) \| \| Function reference \| (Function reference) \| \| ---   G \| \| \| Getting Started \| (Getting Started) \| \| Grid \| uix.Grid (Function reference: 3.1) \| \| GridFlex \| uix.GridFlex (Function reference: 3.2) \| \| Grids \| Grids (Function reference: 3) \| \| GUIDE \| Using layouts inside GUIDE GUIs (User guide: 5) \| \| ---   H \| \| \| HBox \| uix.HBox (Function reference: 2.1) \| \| HBoxFlex \| uix.HBoxFlex (Function reference: 2.3) \| \| HButtonBox \| uix.HButtonBox (Function reference: 2.5) \| \| Help \| Support (Getting Started: 5) \| \| Horizontal button layout \| uix.HButtonBox (Function reference: 2.5) \| \| Horizontal layout \| uix.HBox (Function reference: 2.1) \| \|  \| uix.HBoxFlex (Function reference: 2.3) \| \| ---   I \| \| \| Installation \| Installation (Getting Started: 4) \| \| Installation folder \| layoutRoot (Function reference: 4.2) \| \|  \| \| \|  \| \| \| ---   L \| \| \| Layout basics \| Layout basics (User guide: 1.1) \| \| Layout heirarchies \| Layout heirarchies (User guide: 1.4) \| \| layoutRoot \| layoutRoot (Function reference: 4.2) \| \| ---   M \| \| \| MATLAB Compiler \| Deploying GUIs using the MATLAB Compiler (User guide: 6) \| \| Minimize and maximize \| Minimize and maximize (User guide: 4.2) \| \| Minimum sizes \| Sizes and units (User guide: 1.3.1) \| \|  \| \| \| ---   O \| \| \| onListSelection \| onListSelection (User guide: 7.4) \| \| Other functions \| Other functions (Function reference: 4) \| \| ---   P \| \| \| Panel \| uix.Panel (Function reference: 1.1) \| \| Panels \| Panels (Function reference: 1) \| \| Position vs OuterPosition \| Position vs OuterPosition (User guide: 2.1) \| \| Positioning Axes \| Positioning Axes (User guide: 2) \| \|  \| \| \| ---   R \| \| \| Release notes \| Release notes (Getting Started: 3) \| \| Root \| layoutRoot (Function reference: 4.2) \| \| Running it \| Running it (User guide: 7.5) \| \| ---   S \| \| \| Scalability \| Scalability (User guide: 7.6) \| \| Sizes and units \| Sizes and units (User guide: 1.3) \| \| Support \| Support (Getting Started: 5) \| \| ---   T \| \| \| TabPanel \| uix.TabPanel (Function reference: 1.4) \| \| Types of layout \| Types of layout (User guide: 1.2) \| \| ---   U \| \| \| uix.BoxPanel \| uix.BoxPanel (Function reference: 1.3) \| \| uix.CardPanel \| uix.CardPanel (Function reference: 1.2) \| \| uix.Empty \| uix.Empty (Function reference: 4.1) \| \| uix.Grid \| uix.Grid (Function reference: 3.1) \| \| uix.GridFlex \| uix.GridFlex (Function reference: 3.2) \| \| uix.HBox \| uix.HBox (Function reference: 2.1) \| \| uix.HBoxFlex \| uix.HBoxFlex (Function reference: 2.3) \| \| uix.HButtonBox \| uix.HButtonBox (Function reference: 2.5) \| \| uix.Panel \| uix.Panel (Function reference: 1.1) \| \| uix.TabPanel \| uix.TabPanel (Function reference: 1.4) \| \| uix.VBox \| uix.VBox (Function reference: 2.2) \| \| uix.VBoxFlex \| uix.VBoxFlex (Function reference: 2.4) \| \| uix.VButtonBox \| uix.VButtonBox (Function reference: 2.6) \| \| Understanding layouts \| Understanding layouts (User guide: 1) \| \| Undock \| Dock and undock (User guide: 4.3) \| \| Uninstall \| Installation (Getting Started: 4) \| \| updateInterface \| updateInterface (User guide: 7.3) \| \| User guide \| (User guide) \| \| Using layouts inside GUIDE GUIs \| Using layouts inside GUIDE GUIs (User guide: 5) \| \| ---   V \| \| \| VBox \| uix.VBox (Function reference: 2.2) \| \| VBoxFlex \| uix.VBoxFlex (Function reference: 2.4) \| \| VButtonBox \| uix.VButtonBox (Function reference: 2.6) \| \| Vertical button layout \| uix.VButtonBox (Function reference: 2.6) \| \| Vertical layout \| uix.VBox (Function reference: 2.2) \| \|  \| uix.VBoxFlex (Function reference: 2.4) \| \| Visible example \| Visible example (User guide: 3.1) \| \| ---   W \| \| \| What is GUI Layout Toolbox? \| What is GUI Layout Toolbox? (Getting Started: 1) \| \| Why use layouts? \| Why use layouts? (User guide: 1.5) \| \|  \| \| \|  \| \| \|  \| \| |

  

Back to Top

© 2014 The MathWorks Inc
• Terms of Use
•  Patents
•  Trademarks
