# Supplementary material for: Smad4 controls signaling robustness and morphogenesis by differentially contributing to the Nodal and BMP pathways
Source: Nat Commun. 2021 Nov 4;12:6374. doi: 10.1038/s41467-021-26486-3 (PMC8569018; doi:10.1038/s41467-021-26486-3)
Supplement: Supplementary file 10 — Software code [file 41467_2021_26486_MOESM10_ESM.zip › ALYtools-master 08.21/HelperFunctions/GUI Layout Toolbox/layoutdoc/User guide7_6.html]

Scalability


|  |  |
| --- | --- |
| **User guide** |  |

  

## 7.6: Scalability

As applications get bigger the code gets more complex. The simple
application structure used here does not scale well to large applications,
however some small adjustments can make life much better:

- Convert the "data" structure into a handle object. This allows
  a single "data" object to be shared between mutliple graphical interfaces, and in turn means that the interface
  need not be built as a single monolithic entity.
- Use the events system to trigger updates
  to specific parts of the GUI in response to bits of the data object changing. This removes the need
  for a single large "UpdateInterface" function and reduces coupling between parts of the interface. For example,
  the "SelectedDemo" property would have an associated event such that when it is changed by a callback (or from the command-line) it
  notifies other interface components of the change. Each interface component (or group thereof) can
  just listen for the events that affect it.

Advice on how to build large-scale applications is beyond the scope
of this document. If you need help in this area, please contact your MathWorks
account manager who will be able to put you in touch with a technical specialist.

  

|  |  |  |  |  |
| --- | --- | --- | --- | --- |
|  | Running it | [Top] | Front page |  |

© 2014 The MathWorks Ltd
• Terms of Use
• Patents
• Trademarks
>
