# Supplementary material for: Smad4 controls signaling robustness and morphogenesis by differentially contributing to the Nodal and BMP pathways
Source: Nat Commun. 2021 Nov 4;12:6374. doi: 10.1038/s41467-021-26486-3 (PMC8569018; doi:10.1038/s41467-021-26486-3)
Supplement: Supplementary file 10 — Software code [file 41467_2021_26486_MOESM10_ESM.zip › ALYtools-master 08.21/HelperFunctions/GUI Layout Toolbox/layoutdoc/User guide1.html]

 


Understanding layouts


|  |  |
| --- | --- |
| **User guide** |  |

## 1: Understanding layouts

The purpose of a "layout" is to manage the positioning of one
or more user-interface components. This means that instead of
worrying about the exact position of each user interface component
you instead worry about the relative positioning of them; are they
arranged in a vertical list, horizontal list, a grid pattern, etc.

The best way to understand what layouts are and why they are a good user-interface
design tool is to see some examples. The sections below go through the
basics of building an interface using layouts and how to build complex
interfaces using them.

### Section contents:


- 1.1. Layout basics
- 1.2. Types of layout
- 1.3. Sizes and units
- 1.4. Layout heirarchies
- 1.5. Why use layouts?

  

|  |  |  |  |  |
| --- | --- | --- | --- | --- |
|  | User guide | [Top] | Layout basics |  |

© 2014 The MathWorks Ltd
• Terms of Use
• Patents
• Trademarks
