# Supplementary material for: Smad4 controls signaling robustness and morphogenesis by differentially contributing to the Nodal and BMP pathways
Source: Nat Commun. 2021 Nov 4;12:6374. doi: 10.1038/s41467-021-26486-3 (PMC8569018; doi:10.1038/s41467-021-26486-3)
Supplement: Supplementary file 10 — Software code [file 41467_2021_26486_MOESM10_ESM.zip › ALYtools-master 08.21/HelperFunctions/GUI Layout Toolbox/layoutdoc/User guide3_1.html]

Visible example


|  |  |
| --- | --- |
| **User guide** |  |

  

## 3.1: Visible example

(The code for this example can be found here:
[ view
| edit
| run ]
)

This example shows how the `Visible` property can be
used to hide whole sections of an interface.

Open a window and add a panel

```
fig = figure( 'Name', 'Visible example', ...
    'Position', [100 100 150 250], ...
    'MenuBar', 'none', ...
    'ToolBar', 'none', ...
    'NumberTitle', 'off' );
panel = uix.BoxPanel( 'Parent', fig, 'Title', 'Panel' );
```


Put some buttons inside the panel

```
box = uix.VButtonBox( 'Parent', panel );
uicontrol( 'Parent', box, 'String', 'Button 1' );
uicontrol( 'Parent', box, 'String', 'Button 2' );
uicontrol( 'Parent', box, 'String', 'Button 3', 'Visible', 'off' );
uicontrol( 'Parent', box, 'String', 'Button 4' );
uicontrol( 'Parent', box, 'String', 'Button 5', 'Visible', 'off' );
uicontrol( 'Parent', box, 'String', 'Button 6' );
```


Try hiding the panel

```
set( panel, 'Visible', 'off' );
```


Try showing the panel. Note that the original `Visible` state of each button is remembered.

```
set( panel, 'Visible', 'on' );
```

  

|  |  |  |  |  |
| --- | --- | --- | --- | --- |
|  | Controlling visibility | [Top] | Advanced maneuvers with panels |  |

© 2014 The MathWorks Ltd
• Terms of Use
• Patents
• Trademarks
