# Supplementary material for: Smad4 controls signaling robustness and morphogenesis by differentially contributing to the Nodal and BMP pathways
Source: Nat Commun. 2021 Nov 4;12:6374. doi: 10.1038/s41467-021-26486-3 (PMC8569018; doi:10.1038/s41467-021-26486-3)
Supplement: Supplementary file 10 — Software code [file 41467_2021_26486_MOESM10_ESM.zip › ALYtools-master 08.21/HelperFunctions/GUI Layout Toolbox/layoutdoc/Getting Started2.html]

 


Compatibility considerations


|  |  |
| --- | --- |
| **Getting Started** |  |

## 2: Compatibility considerations

### Section contents:

- 2.1. Minimum MATLAB version
- 2.2. Compatibility with version 1


  

---

## 2.1: Minimum MATLAB version

A new MATLAB graphics system was introduced in R2014b.

This is version 2 of GUI Layout Toolbox, designed to
work with MATLAB releases R2014b and later.

For earlier MATLAB releases using the old MATLAB graphics system,
use version 1.

  

---

## 2.2: Compatibility with version 1

If you are upgrading from version 1, there are a number of
compatibility considerations:

#### Package name

Version 1 packages were contained in the package "uiextras". Version 2 classes are
contained in the package "uix". In version 2, a package "uiextras" is included
to provide support for legacy code. Classes in "uiextras" extend
corresponding classes in "uix", and contain only compatibility-related
code.

#### Contents property

The contents of version 1 objects were accessible via the property `Children`.
The contents of version 2 objects are accessible via the property `Contents`.
Version 2 objects also provide a property `Children`, but this controls the
vertical stacking order of contents, rather than the layout order. Legacy
code that accesses `Children` will run without error, but will not achieve
the desired change in layout order, and should be modified to access
`Contents` instead.

An upcoming release of version 1 will include support for code that references
contents via `Contents`. That way, code modified to work in version 2 will also
work in version 1.

The background to this change is as follows. Version 1 objects were wrappers
for built-in graphics objects, and presented contents in layout order via
the property `Children`. Version 2 objects extend built-in graphics objects,
and as such, inherit properties, methods and events. One such property is
`Children` which is used to control the top-to-bottom stacking order.
MATLAB stacking rules, e.g. controls are always on top of axes, mean that
some reasonable layout orders may be invalid stacking orders, so a new
property for layout order is required.

#### Auto-parenting

The new MATLAB graphics system introduces unparented objects, i.e. those with property `Parent`
empty. The new system also introduces a separation between formal class constructors, e.g.
`matlab.ui.container.Panel`, and informal construction functions, e.g. `uipanel`.
Construction functions are auto-parenting, i.e. if `Parent` is not specified then it is set to
`gcf`, whereas class constructors return objects with `Parent` empty unless
explicitly specified. Version 2 presents a formal interface of class constructors which follow this new
convention.

Classes in "uiextras" are auto-parenting so the behavior of legacy code is
unchanged. However, best practice is to specify parent explicitly during
construction.

#### Defaults mechanism

Version 1 provided a defaults mechanism (`get`, `set` and `unset`
in "uiextras") that mimicked `get` and `set` in the MATLAB graphics
system itself. This feature has been removed from version 2. Users should use an alternative
programming pattern, e.g. factory function, to create objects with standard settings.

#### Enable and disable

Version 1 provided a mechanism to enable and disable container contents using
the property 'Enable'. This feature has been removed from version 2. Users
should enable and disable controls directly rather than via containers.

For more commentary, see this article.

#### Other property name changes

A number of property names have changed to achieve greater consistency
across the package. For example, `RowSizes` and `ColumnSizes` in
uiextras.Grid are now `Heights` and `Widths` in uix.Grid. The package
"uiextras" provides support for legacy property names.

- `RowSizes` in "uiextras" is `Heights` in "uix"
- `ColumnSizes` in "uiextras" is `Widths` in "uix"
- `ShowMarkings` in "uiextras" is `DividerMarkings` in "uix"

#### Property shape changes

Version 2 contents companion properties are now of the same size as `Contents`,
i.e. column vectors. In version 1, these properties were row vectors. The
package "uiextras" provides support for legacy property values.

#### Tab selection behavior

In version 1, after adding a tab to a tab panel, the new tab is selected.

In version 2, the original selection is preserved, except if the tab panel was
empty, in which case the new tab is selected. This is consistent with the
behavior of `uitabgroup`.

  

|  |  |  |  |  |
| --- | --- | --- | --- | --- |
|  | What is GUI Layout Toolbox? | [Top] | Release notes |  |

© 2014 The MathWorks Ltd
• Terms of Use
• Patents
• Trademarks
