# Supplementary material for: Smad4 controls signaling robustness and morphogenesis by differentially contributing to the Nodal and BMP pathways
Source: Nat Commun. 2021 Nov 4;12:6374. doi: 10.1038/s41467-021-26486-3 (PMC8569018; doi:10.1038/s41467-021-26486-3)
Supplement: Supplementary file 10 — Software code [file 41467_2021_26486_MOESM10_ESM.zip › ALYtools-master 08.21/HelperFunctions/GUI Layout Toolbox/layoutdoc/Getting Started3.html]

 


Release notes


|  |  |
| --- | --- |
| **Getting Started** |  |

## 3: Release notes

The GUI Layout Toolbox version numbers take the form `major.minor.iter`. The
current version you have installed can be checked by typing `ver`
at the MATLAB command prompt.

### Section contents:

- 3.1. Version 2.1


  

---

## 3.1: Version 2.1

- Released 2 October 2014
- Initial version for MATLAB R2014b

  

|  |  |  |  |  |
| --- | --- | --- | --- | --- |
|  | Compatibility considerations | [Top] | Installation |  |

© 2014 The MathWorks Ltd
• Terms of Use
• Patents
• Trademarks
