# Supplementary material for: Smad4 controls signaling robustness and morphogenesis by differentially contributing to the Nodal and BMP pathways
Source: Nat Commun. 2021 Nov 4;12:6374. doi: 10.1038/s41467-021-26486-3 (PMC8569018; doi:10.1038/s41467-021-26486-3)
Supplement: Supplementary file 10 — Software code [file 41467_2021_26486_MOESM10_ESM.zip › ALYtools-master 08.21/HelperFunctions/GUI Layout Toolbox/layoutdoc/User guide4_2.html]

Minimize and maximize


|  |  |
| --- | --- |
| **User guide** |  |

  

## 4.2: Minimize and maximize

When a `uix.BoxPanel` has its `MinimizeFcn`
filled in, a minimize/maximize button ( / ) is shown in the upper-right of the
title-bar. When the user clicks this button the specified function
is called. Since the behaviour of the parent container is
different in different use-cases, it is up to the user to write
some code to actually resize the panel. Note that minimizing
a panel to its title-bar only really makes sense inside a
`uix.VBox` or `uix.VBoxFlex`.

The following simple example shows how to add minimize/maximize
functionality to a box full of panels. Save the code into
a file called "dockexample.m" to run it.

(The code for this example can be found here:
[ view
| edit
| run ]
)

#### Create the layout with three panels

Open a new figure window and add three panels.

```
function minimizeexample()
width = 200;
pheightmin = 20;
pheightmax = 100;

% Create the window and main layout
fig = figure( 'Name', 'Collapsable GUI example', ...
              'NumberTitle', 'off', ...
              'Toolbar', 'none', ...
              'MenuBar', 'none' );
box = uix.VBox( 'Parent', fig );

panel{1} = uix.BoxPanel( 'Title', 'Panel 1', 'Parent', box );
panel{2} = uix.BoxPanel( 'Title', 'Panel 2', 'Parent', box );
panel{3} = uix.BoxPanel( 'Title', 'Panel 3', 'Parent', box );
set( box, 'Heights', pheightmax*ones(1,3) );

% Add some contents.
uicontrol( 'Style', 'PushButton', 'String', 'Button 1', 'Parent', panel{1} );
uicontrol( 'Style', 'PushButton', 'String', 'Button 2', 'Parent', panel{2} );
uicontrol( 'Style', 'PushButton', 'String', 'Button 3', 'Parent', panel{3} );

% Resize the window
pos = get( fig, 'Position' );
set( fig, 'Position', [pos(1,1:2),width,sum(box.Heights)] );
```

#### Add the minimize/maximize callback

We set each panel to call the same minimize/maximize function.
This function is nested inside the main function so that it has access
to the main function's variables. A better way to do this is to make the
main function into a class, but this nested-function approach is fine
for simple applications.

Note that as soon as we set the "MinimizeFcn" property the minimize/maximize
icon appears in the top-right of each panel. We use a cell-array to pass an
extra argument, the panel number, to the minimize function. This extra argument appears after the usual
`eventSource` and `eventData` arguments.

```
% Hook up the minimize callback.
set( panel{1}, 'MinimizeFcn', {@nMinimize, 1} );
set( panel{2}, 'MinimizeFcn', {@nMinimize, 2} );
set( panel{3}, 'MinimizeFcn', {@nMinimize, 3} );

%-------------------------------------------------------------------------%  
 
    function nMinimize( eventSource, eventData, whichpanel )
        % A panel has been maximized/minimized
        s = get( box, 'Heights' );
        pos = get( fig, 'Position' );
        panel{whichpanel}.IsMinimized = ~panel{whichpanel}.IsMinimized;
        if panel{whichpanel}.IsMinimized
            s(whichpanel) = pheightmin;
        else
            s(whichpanel) = pheightmax;
        end 
        set( box, 'Heights', s );
        
        % Resize the figure, keeping the top stationary
        delta_height = pos(1,4) - sum( box.Heights );
        set( fig, 'Position', pos(1,:) + [0 delta_height 0 -delta_height] );
    end % Minimize 

end % Main function
```

#### Click the minimize buttons

Minimizing the middle panel causes it to shrink to just its
title-bar and the window shrinks accordingly. The
"Minimize" icon is replaced by a "Maximise" icon.


Re-maximizing the panel would
cause it to re-appear in full and the window to grow again.

  

|  |  |  |  |  |
| --- | --- | --- | --- | --- |
|  | Context help | [Top] | Dock and undock |  |

© 2014 The MathWorks Ltd
• Terms of Use
• Patents
• Trademarks
